# PLA2G2D in tumour-draining lymph nodes regulates anti-tumour immunity

**DOI:** 10.1038/s41586-026-10954-1

**Published:** 2026-09-09

**Authors:** Anneloes van Krimpen, Julie Huang, Mike Eterman, Vivian Gerretsen, Michihisa Umetani, Josephine C. Janssen, Menno van Nimwegen, Nina Rozendaal, Thierry P. P. van den Bosch, Xinguo Jiang, Kathryn Logronio, Angela Z. Liu, Yun-Ru Liu, Yuki Nagasaki, Makoto Murakami, Anne Onrust-Van Schoonhoven, Asabi Leliveld, Disha Vadgama, Hedwig Langeveld, Rogier van Wijck, Eric Bindels, Jan von der Thüsen, Antien Mooyaart, Febe van Maldegem, Claudia M. Brenis, Stijn Verwaerde, Rudi W. Hendriks, Bart N. Lambrecht, Dirk J. Grünhagen, Cornelis Verhoef, Joachim G. J. V. Aerts, Li-Fen Lee, Kan V. Lu, Ralph Stadhouders, Floris Dammeijer

**Affiliations:** 1 Department of Pulmonary Medicine, Erasmus MC University Medical Centre, Rotterdam, The Netherlands; 2 Erasmus MC Cancer Institute, Erasmus MC University Medical Centre, Rotterdam, The Netherlands; 3 Apeximmune Therapeutics, Burlingame, CA USA; 4 Laboratory of Immunoregulation and Mucosal Immunology, VIB-UGent Centre for Inflammation Research, Ghent, Belgium; 5 Department of Pharmacological and Pharmaceutical Sciences, University of Houston, Houston, TX USA; 6 Department of Surgical Oncology, Erasmus MC University Medical Centre, Rotterdam, The Netherlands; 7 Department of Pathology, Erasmus MC University Medical Centre, Rotterdam, The Netherlands; 8 Taipei Medical University, Taipei City, Taiwan; 9 Center for Disease Biology and Integrative Medicine, Graduate School of Medicine, University of Tokyo, Tokyo, Japan; 10 Department of Haematology, Erasmus MC University Medical Centre, Rotterdam, The Netherlands; 11 Department of Molecular Cell Biology and Immunology, Amsterdam UMC, Amsterdam, The Netherlands; 12 Department of Internal Medicine and Pediatrics, Ghent University, Ghent, Belgium

**Keywords:** Tumour immunology, Cancer therapy

## Abstract

Systemic anti-tumour immunity results from T cell priming in tumour-draining lymph nodes (TDLNs)^1–4^. Although the suppression of T cells in tumours is well characterized^5–8^, whether this occurs in TDLNs—and if so, through which mechanisms—remains poorly understood. Here, using imaging mass cytometry of TDLNs from patients with melanoma, we identify a spatial neighbourhood in the TDLN paracortex that is linked to the development of distant metastases. Targeted spatial transcriptomics of cells inside this neighbourhood revealed activated CD8^+^ T cells engaging with myeloid cells that expressed high levels of the immunosuppressive secretory phospholipase PLA2G2D. PLA2G2D^+^ myeloid cells were substantially more abundant in TDLNs than they were in primary tumours or metastases. Genetic loss-of-function or antibody-mediated inhibition of PLA2G2D reduced tumour growth markedly, and single-cell transcriptomics in melanoma-bearing mice revealed that expression of *Pla2g2d* is confined to lymph-node macrophages. Mechanistically, PLA2G2D directly suppressed the early proliferation of T cells in vitro, and inhibiting PLA2G2D resulted in an expansion of tumour-specific T cells in TDLNs, leading to an increase in these T cells in the circulation and subsequently in tumours. Notably, PLA2G2D and PD-1 act as non-redundant immune checkpoints, with combination treatment showing additive or synergistic efficacy in humanized mice treated with human-specific antibodies. Collectively, our in-depth spatial profiling identifies PLA2G2D as a TDLN-centred targetable immune checkpoint for cancer immunotherapy.

## Abstract (web summary)

A specific population of macrophages that express high levels of the enzyme PLA2G2D in tumour-draining lymph nodes is associated with poor prognosis in patients, and inhibiting PLA2G2D improves anti-tumour immunity in mouse models.

## Main

T-cell-driven adaptive immune responses against cancer antigens are a cornerstone of effective tumour control, although immune checkpoint activation limits T cell activity. Studies in preclinical models and early findings in patients indicate that TDLNs are a primary source of tumour-reactive T cells^1–4,9,10^. Immune checkpoint inhibitors (ICIs), such as anti-PD-1 or anti-PD-L1, act in part by enhancing T cell priming and differentiation, thereby inducing a systemic anti-tumour immune response that is crucial for therapy efficacy in multiple tumour types^4,11–14^. Despite the clinical success of ICIs, many patients do not respond, or develop adaptive immune resistance^15,16^. Although the molecular underpinnings of ICI resistance and T cell dysfunction in the tumour microenvironment (TME) have been widely studied, the mechanisms that underlie immune suppression in TDLNs remain mostly unknown. TDLN characteristics are associated with distant disease relapse after melanoma resection, suggesting that TDLNs have a role in generating systemic anti-tumour immunity^11,17^. Although several immune-suppressive pathways known from studies in tumours have been implicated (for example, upregulation of PD-L1), an in-depth understanding of immunoregulatory mechanisms in TDLNs and how these might affect patient outcomes is lacking. We therefore performed comprehensive spatial proteogenomics studies on TDLNs from patients with melanoma, aiming to identify targetable regulators of systemic anti-tumour immunity. We found that TDLN contexture correlates with patient outcomes after surgery. Specifically, we identified lymph node (LN) myeloid cells—predominantly macrophages—that express the secreted phospholipase A2 family member PLA2G2D to be associated with a poor prognosis. PLA2G2D acts as a LN-centred immune checkpoint that curtails anti-tumour T cell proliferation, and genetic or antibody-mediated inhibition of PLA2G2D suppressed tumour growth and synergized with anti-PD-1 ICI in preclinical models. Thus, PLA2G2D acts as a bona fide immune checkpoint in TDLNs and represents a new target for cancer immunotherapy.

## TDLN composition predicts poor prognosis

**Extended Data Fig. 1:**
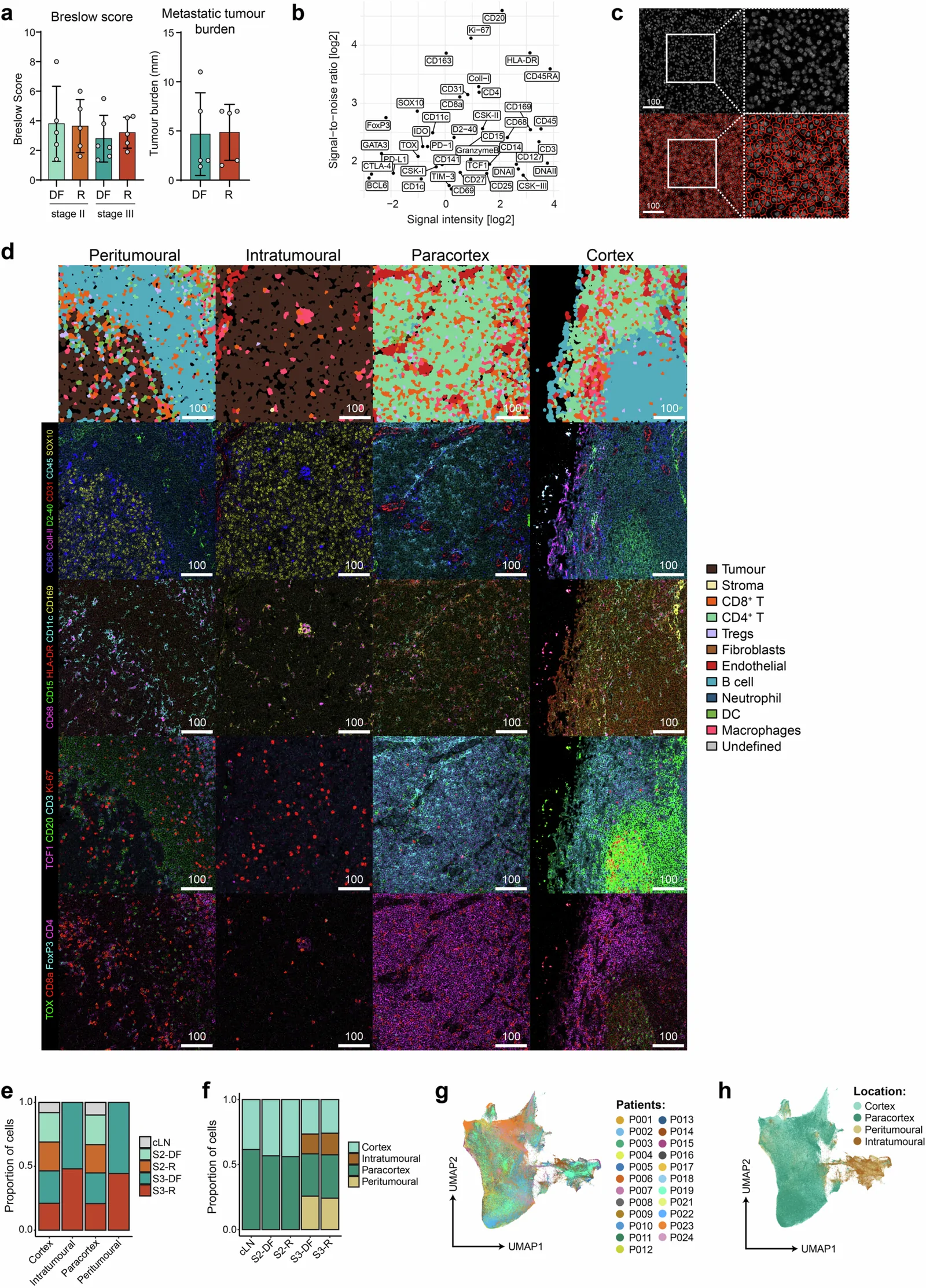
Imaging mass cytometry of lymph nodes in patients with melanoma. (**a**) Breslow score (left) and tumour metastatic tumour burden (right) in the TDLNs of stage II (n = 10) or III (n = 11) patients with melanoma included in the IMC cohort, visualized as mean ± s.d. Patients were divided into those who remained disease free (‘DF’, RFS > 60–96 months) or developed distant disease recurrence (‘R’, RFS < 24–48 months) following surgery (5–6 patients per group). A Kruskal-Wallis test was performed to test for differences (all *P* > 0.05). (**b**) Signal-to-noise ratio (SNR; *y* axis) plotted against signal intensity (*x *axis) for each individual marker in the IMC panel (calculated on a per cell basis). (**c**) CellProfiler cell mask images with nuclear staining (top; white signal) and the corresponding cell mask (bottom, in red). (**d**) Representative images of selected IMC markers for each anatomical location selected in the TDLN. Cell segmentation and annotation is shown on top. (**e**) Proportions of cells derived from the indicated sample groups. Patients with stage II or III melanoma were divided into DF/R groups (see above) and analysed per anatomical location in the TDLN. (**f**) Proportions of cells found per anatomical location across sample groups. (**g**) Patient IDs projected on the batch-corrected UMAP visualization of all cells detected in the IMC experiments. (**h**) Anatomical locations of isolated cells projected on batch-corrected UMAP visualization of all cells detected (IMC).

We collected TDLNs from patients with resected stage II or III melanoma (non-metastatic or metastatic LNs, respectively) who did not receive adjuvant or neoadjuvant therapies and whose clinical outcomes were known. Patients were labelled as ‘recurrence’ (R; stage II: recurrence-free survival (RFS) ≤ 48 months, stage III: RFS ≤ 24 months) or ‘disease-free’ (DF; stage II: RFS > 96 months, stage III: RFS > 60 months). TDLNs were localized using perioperative radiotracer injection at the site of a previous tumour resection in a sentinel node procedure. Patients with melanoma (*n* = 5 or 6 per group) were matched by tumour burden and known prognostic markers such as Breslow depth (Supplementary Table 1 and Extended Data Fig. 1a). Skin-draining (axillary) control LNs (cLNs) from patients who died from acute vascular disease in the absence of cancer, infection or autoimmune disease were also included.

**Fig. 1:**
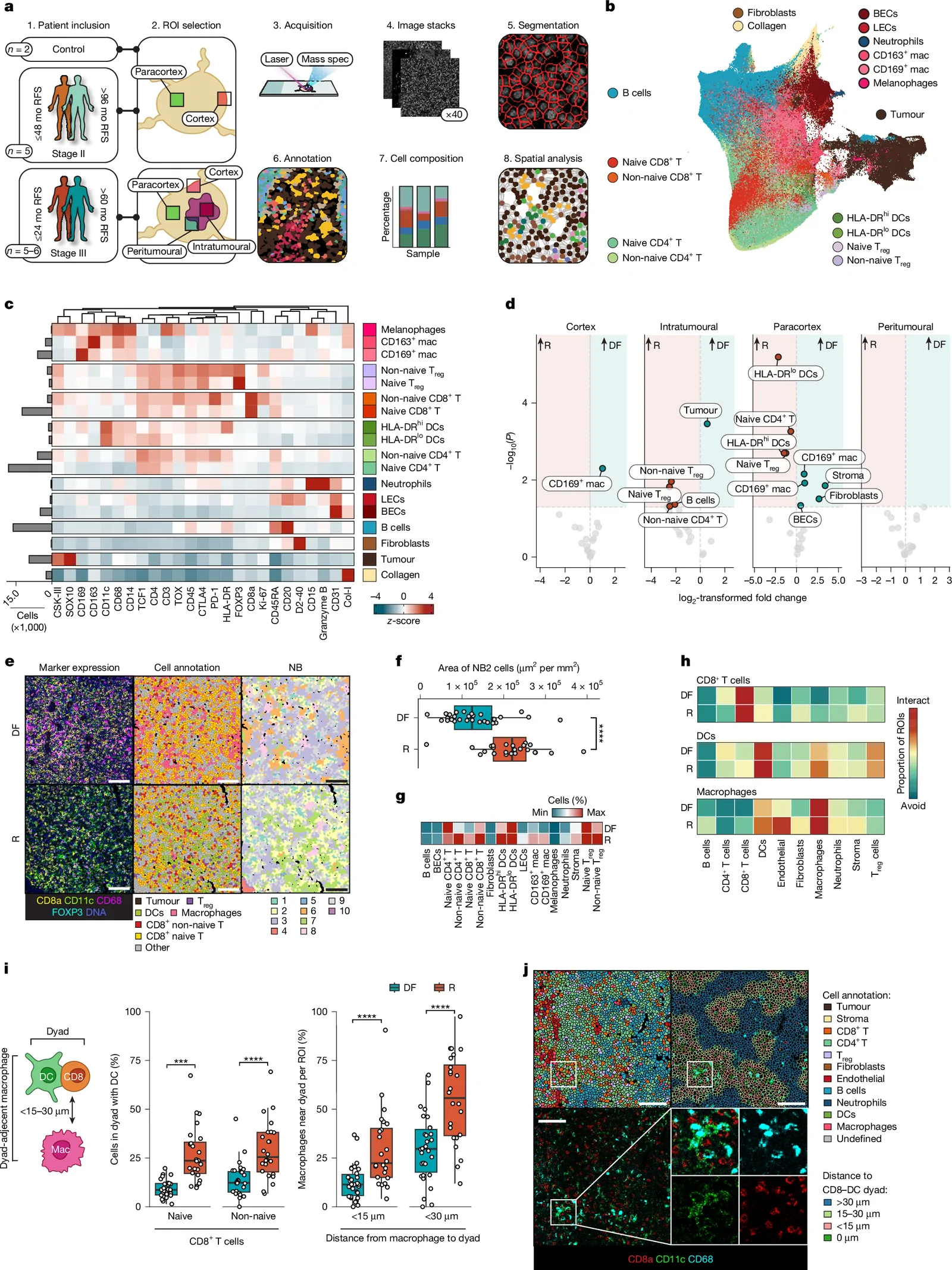
Location-specific changes in TDLN immune contexture are linked with distant disease metastasis. **a**,TDLNs were collected from patients with stage II (non-metastatic) and stage III (metastatic) melanoma who remained disease free (DF; RFS > 60–96 months; mo) or who developed distant disease recurrence (R; RFS < 24–48 months) after surgery. Axillary cLNs from individuals without cancer were also collected. Tissues were stained with an IMC 40-plex antibody panel and imaged across ROIs in cortex, paracortex, peritumoural and intratumoural areas. **b**, Annotated uniform manifold approximation and projection (UMAP) of 870,277 single cells from 372 ROIs. BEC, blood endothelial cell; LEC, lymphatic endothelial cell. **c**, Scaled IMC marker expression for the different cell subsets identified. Bar graphs depict total cell numbers. **d**, Volcano plots showing differences in cell abundance (log_2_-transformed fold change of cells per mm^2^ tissue per ROI) between patients with stage III melanoma in the DF group and in the R group. **e**, Representative IMC images including cell segmentation mask with annotations and cellular NB classifications. Scale bars, 100 μm.** f**, Spatial area occupied by cells associated with NB2 in patients with stage III melanoma in group DF or group R (R = 24 ROIs, DF = 30 ROIs, from 5 or 6 patients, respectively; 4–5 ROIs per patient). **g**, Relative abundance (scaled per cell type across NBs) of the indicated immune-cell subsets in NB2 in patients with stage III melanoma in group DF or group R (paracortex). **h**, Nearest-neighbour interaction analyses of the indicated cells in stage III DF or R paracortex. Colours indicate the proportions of ROIs with statistically significant interaction (+1) or avoidance (−1), corrected for cellular abundance. **i**, Quantification of DC–CD8^+^ T cell co-localization (dyads; left) and macrophages in close proximity (<15 µm or <30 µm) to DC–CD8^+^ T cell dyads (right) in DF or R paracortex (R = 24 ROIs, DF = 30 ROIs, from 5 or 6 patients, respectively). **j**, Visualization of DC–CD8^+^ T cell dyads and adjacent macrophages, showing annotated cells (top left), dyad distance (top right) and marker quantification of CD8a (T cells; red), CD11c (DCs; green) and CD68 (macrophages; cyan) (bottom). Scale bars, 100 μm. *P* values by unpaired two-sided *t*-test (**d**,**f**,**i**). ***P < 0.001, ****P < 0.0001 (exact *P* values in Supplementary Data).

**Extended Data Fig. 2:**
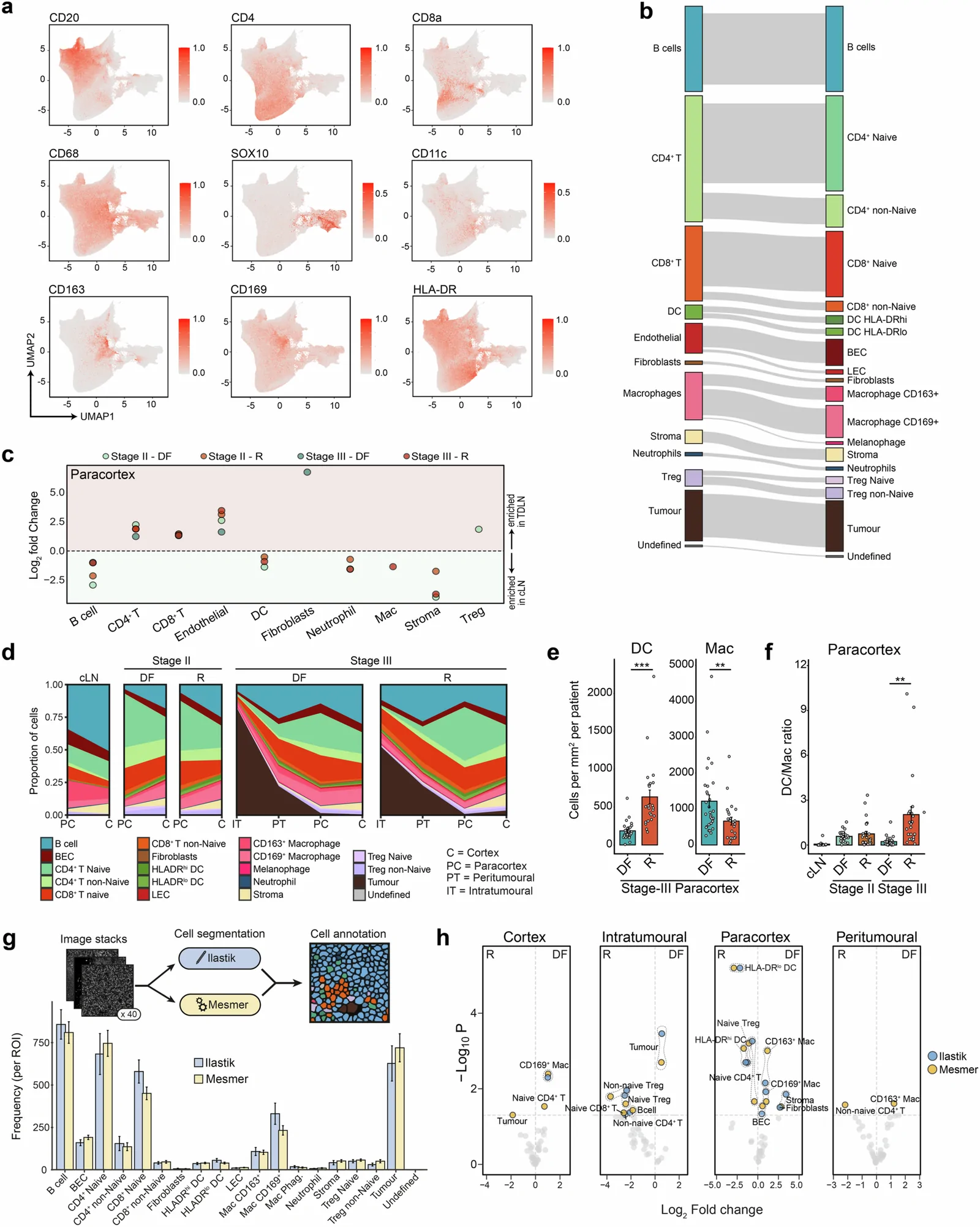
Characterizing the melanoma TDLN microenvironment. (**a**) UMAP projections of scaled expression levels of the indicated marker proteins across all cells identified in the combined IMC experiments. (**b**) Sankey plot showing relationships between two different layers of annotation used in the study: level 1 (left) and level 2 (right). (**c**) Changes in cellular composition depicted as log_2_ fold changes in TDLNs versus cLNs of mean number of cells/mm² tissue. Only those changes with an adjusted *P* < 0.05 are shown. (**d**) Composition of ROIs, separated by location (IT = intratumoral, PT = peritumoral, PC = paracortex, C = cortex) in different disease outcome groups, displayed as proportions of total cells per group. (**e**) Number of macrophages (Mac) and DCs per mm^2^ in the paracortex of the TDLNs of stage III disease-free (DF) and recurrence (R) patients with melanoma, visualized per ROI (R = 24 & DF = 30 ROIs from 5 or 6 patients, respectively; 4–5 ROIs per patient). (**f**) DC/Mac ratio in cLNs and the TDLN paracortex of patients with stage II or III melanoma, visualized per ROI (R = 24 & DF = 30 ROIs from 5 or 6 patients respectively; 4–5 ROIs per patient). (**g**) Comparison of Ilastik and Mesmer cell segmentation pipelines revealed no significant (*P* > 0.05) differences in cell-type abundance across ROI (n = 352 ROIs). (**h**) Volcano plots show similar changes in abundance (cells/mm^2^; *P* < 0.05) of cell types for both segmentation methods when comparing stage III R versus DF patients. Only a small subset of cells showed borderline method-specific significant changes. *P* values were calculated using an unpaired two-sided t-test (**e**–**h**). Data are presented as mean ± s.e.m. (**e**–**g**). *P < 0.05, **P < 0.01, ***P < 0.001, ****P < 0.0001 (Exact *P* values in Supplementary Data).

To map the cellular composition of TDLNs across different anatomical sites, we designed a 40-plex imaging mass cytometry (IMC) antibody panel capable of distinguishing different immune and stromal cell subsets. Regions of interest (ROIs) from cortex, paracortex, peritumoural and intratumoural areas were selected (at least 5 ROIs per location and per patient; 372 ROIs were analysed in total) and protein expression intensities for all markers were acquired at the single-cell level (Fig. 1a and Extended Data Fig. 1b). Cell segmentation identified 870,277 individual cells, which were annotated on the basis of marker expression and included B cells, T cells, dendritic cells (DCs), macrophages and stromal cells (Fig. 1b,c and Extended Data Figs. 1c–h and 2a,b). Immune contexture in melanoma TDLNs differed from that in cLNs, even in the absence of metastasis (stage II) (Extended Data Fig. 2c,d)—in line with previous studies showing TDLN remodelling in patients developing metastases^4,18–20^. Unbiased comparisons of cell abundance revealed compositional changes mainly in the stage III TDLN paracortex, involving a substantial increase in HLA-DR^lo^ and HLA-DR^hi^ DCs in patients in the R group, as opposed to increased levels of CD163^+^ and CD169^+^ macrophages in patients in the DF group (Fig. 1d). Increased DC–macrophage ratios were strongly linked to disease recurrence, but only in metastatic TDLNs (stage III; Extended Data Fig. 2e,f). Differences in cell abundance were independent of the cell segmentation method used (Extended Data Fig. 2g,h).

**Extended Data Fig. 3:**
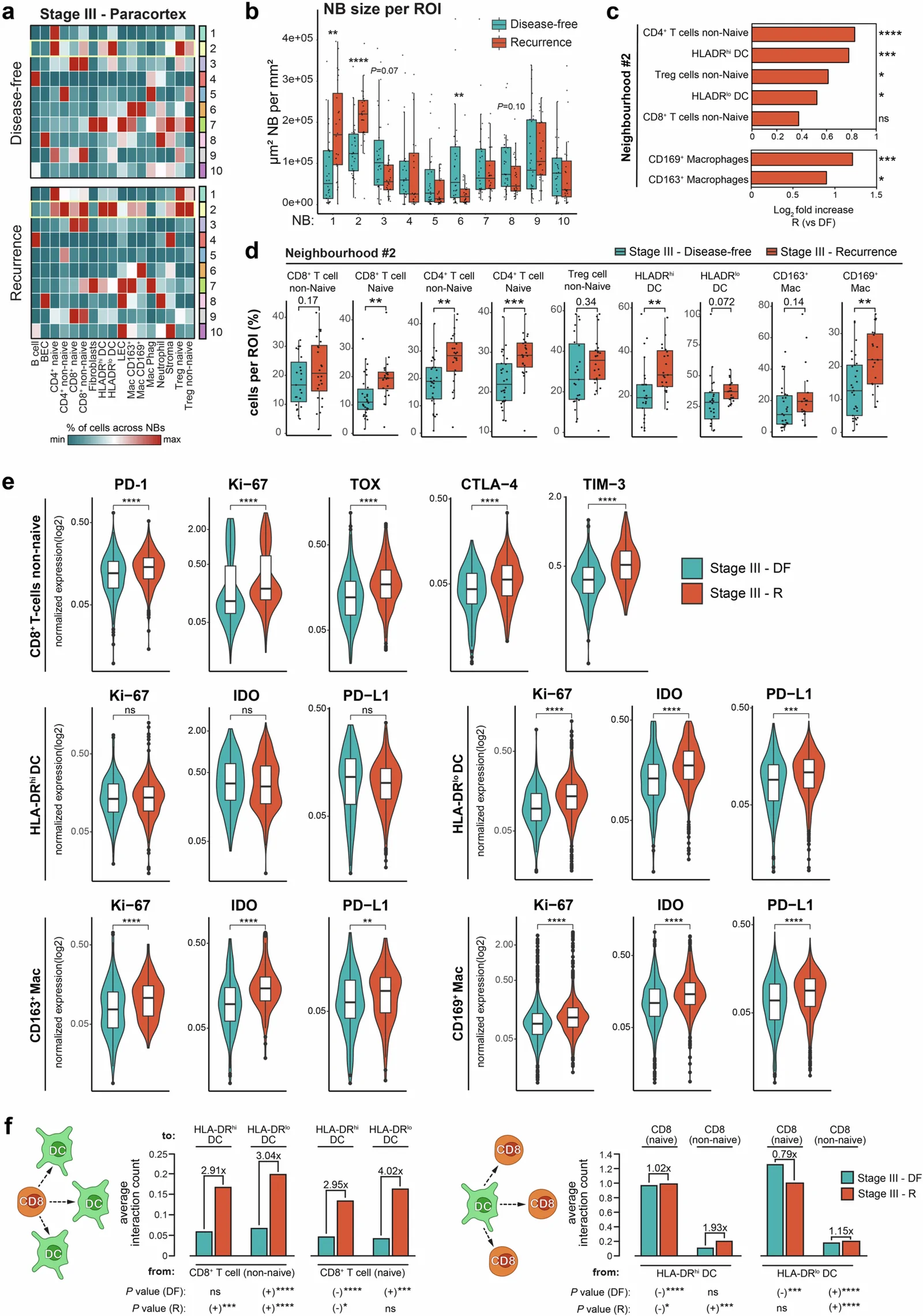
Spatial neighbourhood analysis in melanoma TDLNs. (**a**) Heat map showing relative cellular abundances (min-max scaled per column) for all 10 cellular NBs in disease-free (DF) and recurrence (R) patients with melanoma. NB2 is highlighted by a yellow box. (**b**) Box plots displaying NB size per patient group in stage III melanoma as μm² of NB area per mm² ROI (R = 24 & DF = 30 ROIs from 5 or 6 patients respectively; 4–5 ROIs per patient). (**c**) Log_2_ fold increase of selected cell abundances in NB2 in R versus DF patients. (**d**) Percentage of cells present in NB2 separated by patient group, visualized per ROI (R = 24 & DF = 30 ROIs from 5 or 6 patients respectively; 4–5 ROIs per patient). (**e**) Violin plots displaying marker expression for different cell types, visualized per ROI, within the paracortex. Inverse hyperbolic sine-transformed IMC expression values of indicated protein markers, divided by cell type and patient group. Values are displayed on a log_2_-transformed *y* axis (points represent individual cells). (**f**) Average interaction counts across the entire IMC dataset from CD8^+^ T cells (left side) and from DCs (right side) in the TDLN paracortex. Fold changes between stage III R versus DF are indicated above the bars. Statistical test (based on random cell label permutation) indicates whether the indicated pairwise interaction occurs more than by random chance (i.e. *P* < 0.05) in both patient groups. *P* values were calculated using a two-sided Wilcoxon rank-sum test (**b**,**d**,**e**) or Monte Carlo permutation test (**c**,**f**). DC, dendritic cell; Mac, macrophage; DF, disease free; R, recurrence. *P < 0.05, **P < 0.01, ***P < 0.001, ****P < 0.0001, ns: non-significant (Exact *P* values in Supplementary Data).

To investigate networks of preferential cell–cell interactions, we used nearest-neighbour analysis to define ten cellular neighbourhoods (NBs) in the TDLN paracortex (Fig. 1e and Extended Data Fig. 3a). Several NBs were expanded or reduced in R versus DF patients (Extended Data Fig. 3b), and most of these consisted mainly of a single cell type (for example, T cells in NB1 and NB3; macrophages in NB6) or two cell types (for example, neutrophils interacting with endothelial cells in NB8). NB2, however, was particularly expanded in R compared with DF patients (Fig. 1f), and was characterized by an increased presence of CD4^+^, CD8^+^ and regulatory T (T_reg_) cells as well as HLA-DR^hi^ DCs in R patients (Fig. 1g and Extended Data Fig. 3a,c,d). Notably, the frequencies of CD163^+^ and CD169^+^ macrophages—although generally reduced in the paracortex of R patients—also increased in NB2. Phenotypically, T cells within NB2 in R patients showed increased levels of activation and exhaustion-associated markers such as CTLA4 and TOX, whereas macrophage and DC subsets expressed increased levels of the immunosuppressive molecules PD-L1 and IDO (Extended Data Fig. 3e). Nearest-neighbour and cellular proximity analyses in the paracortex showed that the formation of DC–CD8^+^ T cell dyads increased in R compared with DF patients, and that this was often accompanied by close macrophage proximity (Fig. 1h–j and Extended Data Fig. 3f). Together, these data identify a distinct myeloid–T cell interface in the TDLN paracortex of patients with melanoma who have a poor prognosis.

## Myeloid cells in TDLNs produce PLA2G2D

**Fig. 2:**
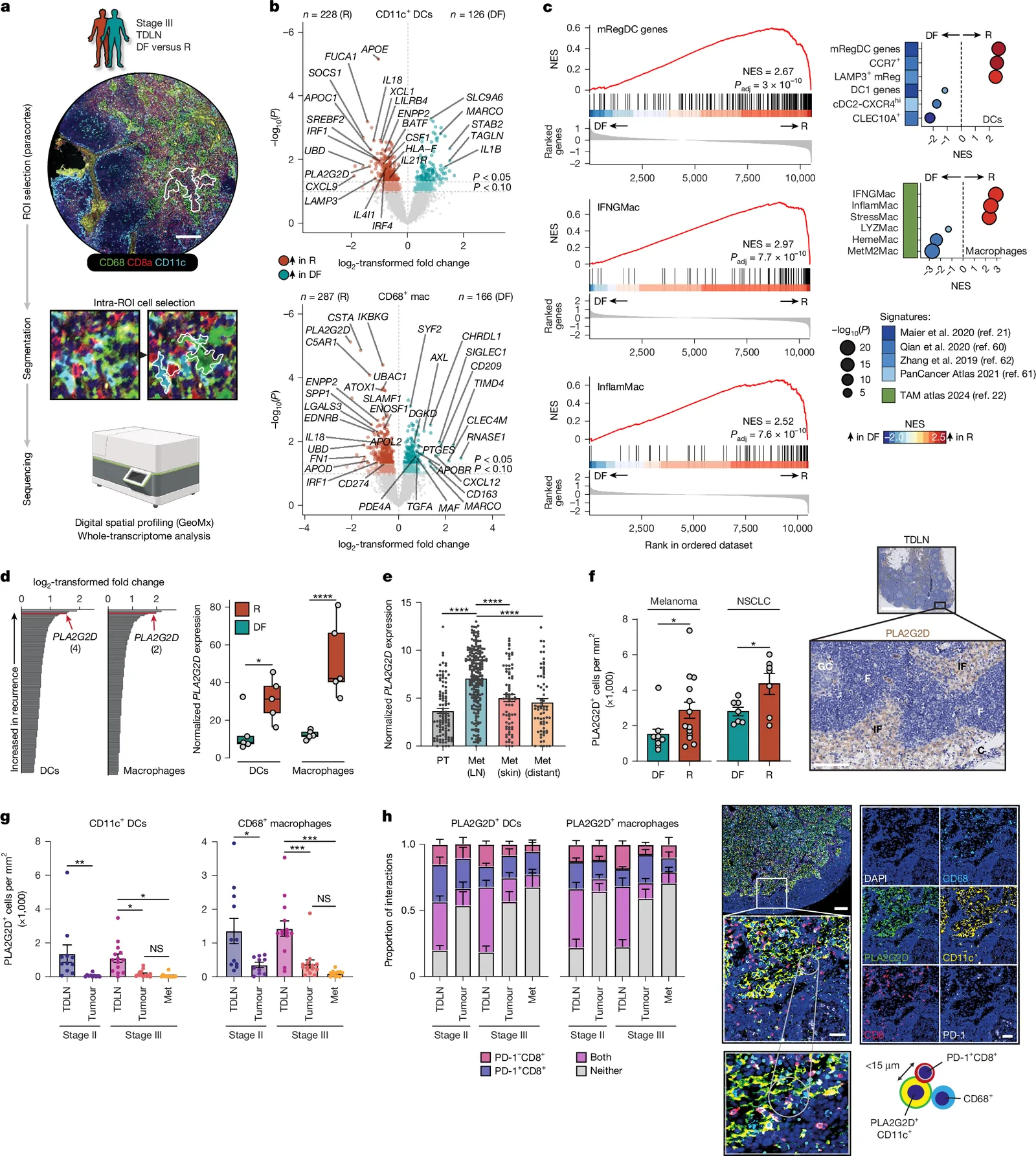
Metastatic recurrence is associated with PLA2G2D expression in TDLN myeloid cells. **a**, Experimental set-up for transcriptomic analysis of co-localizing CD8^+^, CD68^+^ and CD11c^+^ cells in the paracortex of patients with stage III melanoma in the DF and R groups (*n* = 5 per group). Scale bar, 250 μm. Image created partly in BioRender; Van Krimpen, A. https://BioRender.com/8ouveic (2026). **b**, Comparison of DEGs (*P* < 0.05 or *P* < 0.1) in DF and R patients. **c**, GSEA of DEGs using published DC and macrophage transcriptional signatures consisting of specific sets of genes linked to the indicated myeloid cell phenotypes. cDC, conventional dendritic cell; NES, normalized enrichment score. **d**, Ranked lists of DEGs upregulated in the R group versus the DF group. The box plot shows the levels of* PLA2G2D* expression in the indicated cell types in the TDLN paracortex (*n* = 5 patients per group). Numbers in brackets denote rank in DEG list. **e**, Normalized (transcripts per million) levels of *PLA2G2D* RNA in melanoma samples from the indicated origins (TCGA data). PT, primary tumour (*n* = 104); Met, metastasis (LN, *n* = 224; skin, *n* = 74; distant, *n* = 68). **f**, Left, immunohistochemistry quantification of PLA2G2D^+^ cells per mm^2^ in metastasized TDLN tissue samples from independent melanoma (DF, *n* = 11; R, *n* = 13) and non-small cell lung cancer (NSCLC; DF, *n* = 7; R, *n* = 7) cohorts. Right, representative staining of a melanoma TDLN. C, capsule; F, follicle; GC, germinal centre; IF, interfollicular area. Scale bars, 1 mm (top); 100 μm (bottom). **g**, Immunofluorescence quantification of PLA2G2D^+^ DCs and macrophages per mm^2^ TDLN, primary tumour and the TDLN metastasis in stage II (*n* = 11 per group) and stage III (tumour and TDLN, *n* = 14; metastasis, *n* = 12) NSCLC. **h**, Left, relative interaction frequencies (within 15 µm) of PLA2G2D^+^ DCs (left) and macrophages (right) with PD-1^+^ or PD-1^−^ CD8^+^ T cells. Right, representative immunofluorescence staining. Scale bars, 200 μm (top left); 50 μm (middle left and bottom right). All bar graphs denote mean ± s.e.m. *P* values by linear mixed model (**b**,**d**), weighted Kolmogorov–Smirnov-like running-sum statistic (**c**), Wilcoxon rank-sum test (**e**,**f**) or mixed-effects analysis with Geisser–Greenhouse correction and Holm–Šidák multiple-testing correction (**g**,**h**). *P < 0.05, **P < 0.01, ***P < 0.001, ****P < 0.0001; NS, not significant (exact *P* values in Supplementary Data).

**Extended Data Fig. 4:**
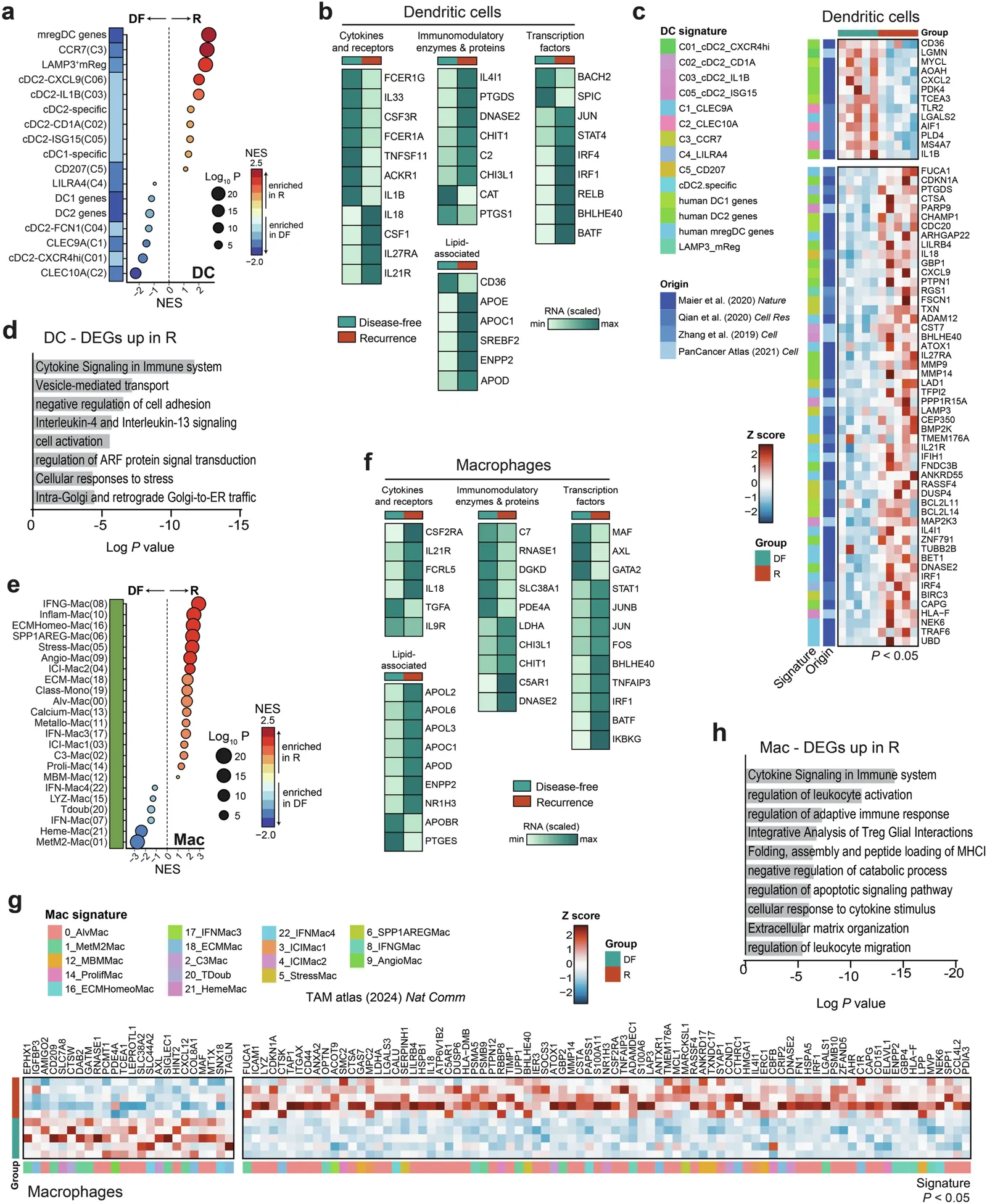
Spatially resolved transcriptome profiling of TDLN myeloid cells. (**a**) GSEA testing the enrichment of indicated transcriptional signatures (as detected by whole-transcriptome GeoMx DSP) for DCs, comparing paracortex stage III cells from recurrence (R) versus disease-free (DF) patients with melanoma. Dot size indicates the adjusted P value; dot colour indicates normalized enrichment scores (NES). (**b**) Scaled average gene expression levels of selected DEGs on the basis of association with the indicated protein groups or functions. (**c**) Heat map with scaled (Z-score) RNA expression levels DEGs (*P* < 0.05) between TDLN paracortex DCs from DF and R patients with stage III melanoma. Columns represent individual patients; associations with published tumour-associated phenotypic signatures are indicated. (**d**) Enrichment of biological pathways among DEGs (*P* < 0.05) upregulated in R versus DF DCs. (**e**) GSEA testing the enrichment of indicated transcriptional signatures (as detected by whole-transcriptome GeoMx DSP) for macrophages (Mac), comparing paracortex stage III cells from recurrence (R) versus disease-free (DF) patients with melanoma. Dot size indicates the adjusted P value; dot colour indicates normalized enrichment scores (NES). (**f**) Scaled average gene expression levels of selected DEGs on the basis of association with the indicated protein groups or functions. (**g**) Heat map with scaled (Z-score) RNA expression levels for DEGs (*P* < 0.05) between TDLN paracortex macrophages from DF and R patients with stage III melanoma. Columns represent individual patients; associations with published tumour-associated phenotypic signatures are indicated. (**h**) Enrichment of biological pathways among all DEGs (*P* < 0.05) upregulated in R versus DF macrophages. *P* values were calculated using a LMM (**c**,**g**), hypergeometric test and Benjamini–Hochberg procedure as implemented by Metascape (**c**,**f**) or weighted Kolmogorov–Smirnov-like running-sum statistic, as implemented by GSEA (**a**,**d**).

**Extended Data Fig. 5:**
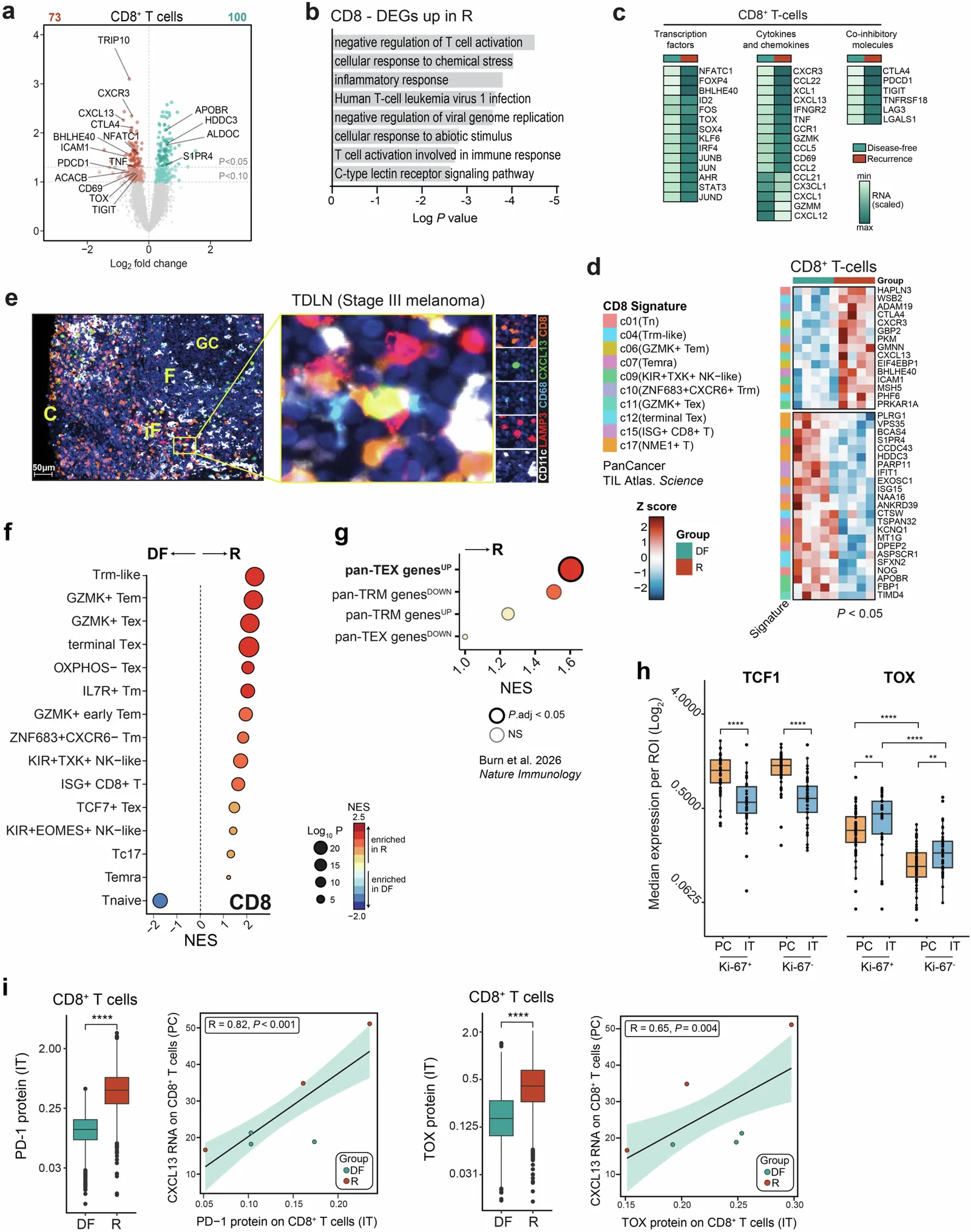
Phenotyping of CD8^+^ T cells in TDLNs. (**a**) Volcano plot of DEGs (P < 0.05 or P < 0.1; detected by whole-transcriptome GeoMx DSP) in CD8^+^ T cells from the TDLN paracortex comparing disease-free (DF) and recurrence (R) patients with stage III melanoma (n = 5 per group). (**b**) Enrichment of biological pathways among all DEGs (*P* < 0.05) upregulated in R versus DF CD8^+^ T cells. (**c**) Scaled average gene expression levels of selected DEGs (from **a**) based on associated with the indicated protein groups or functions. (**d**) Heat map with scaled (Z-score) RNA expression levels for DEGs (*P* < 0.05) between TDLN paracortex CD8^+^ T cells from DF and R patients with stage III melanoma. Columns represent individual patients; associations with published tumour-associated phenotypic signatures are indicated. (**e**) Representative immunofluorescence image of a representative paracortex ROI displaying CD8, CD68 and CD11c-positive cells with LAMP3 and CXCL13 expression marking mRegDCs and activated CD8^+^ T cells, respectively. GC: germinal centre, F: follicle, IF: interfollicular area, C: capsule. (**f**,**g**) GSEA testing the enrichment of indicated transcriptional signatures (as detected by whole-transcriptome GeoMx DSP) for CD8^+^ T cells, comparing paracortex stage III cells from R versus DF patients with melanoma. Dot size indicates the adjusted *P* value; dot colour indicates normalized enrichment scores (NES). In **f**, pathways passing statistical significance (i.e. pan-TEX genes^UP^) are indicated by a black line; all pathways shown in **e** are statistically significant. (**h**) Box plots displaying marker expression for different cell types, visualized per ROI (R = 24 & DF = 30 ROIs from 5 or 6 patients respectively; 4–5 ROIs per patient), within the paracortex. Inverse hyperbolic sine-transformed IMC protein levels for TCF1 and TOX are shown in CD8^+^ T cells, divided by Ki-67 status. Values are displayed for the paracortex (PC) and intratumoral (IT) zone of the TDLN, on a log_2_-transformed *y* axis (points represent individual ROIs). (**i**) Box plots: normalized PD-1 (left) and TOX (right) protein levels from IMC analysis of intratumoral (IT) CD8^+^ T cells (DF = 45,859 cells; R = 41,100 cells), comparing DF (n = 6) and R (n = 5) patients with stage III melanoma. Correlation plots (Pearson) denote associations between *CXCL13* RNA levels (GeoMx) in CD8^+^ T cells from the paracortex (PC) and the IT PD-1 (left) or TOX (right) levels (IMC) on CD8^+^ T cells from the same patients across both outcome groups. *P* values were calculated using a two-sided Wilcoxon rank-sum test (**h**,**i**), a LMM (**a**,**d**), hypergeometric test and the Benjamini–Hochberg (BH) procedure as implemented by Metascape (**b**), or weighted Kolmogorov–Smirnov–like running-sum statistic as implemented by GSEA (**f**,**g**). **P < 0.01, ****P < 0.0001 (Exact *P* values in Supplementary Data).

**Extended Data Fig. 6:**
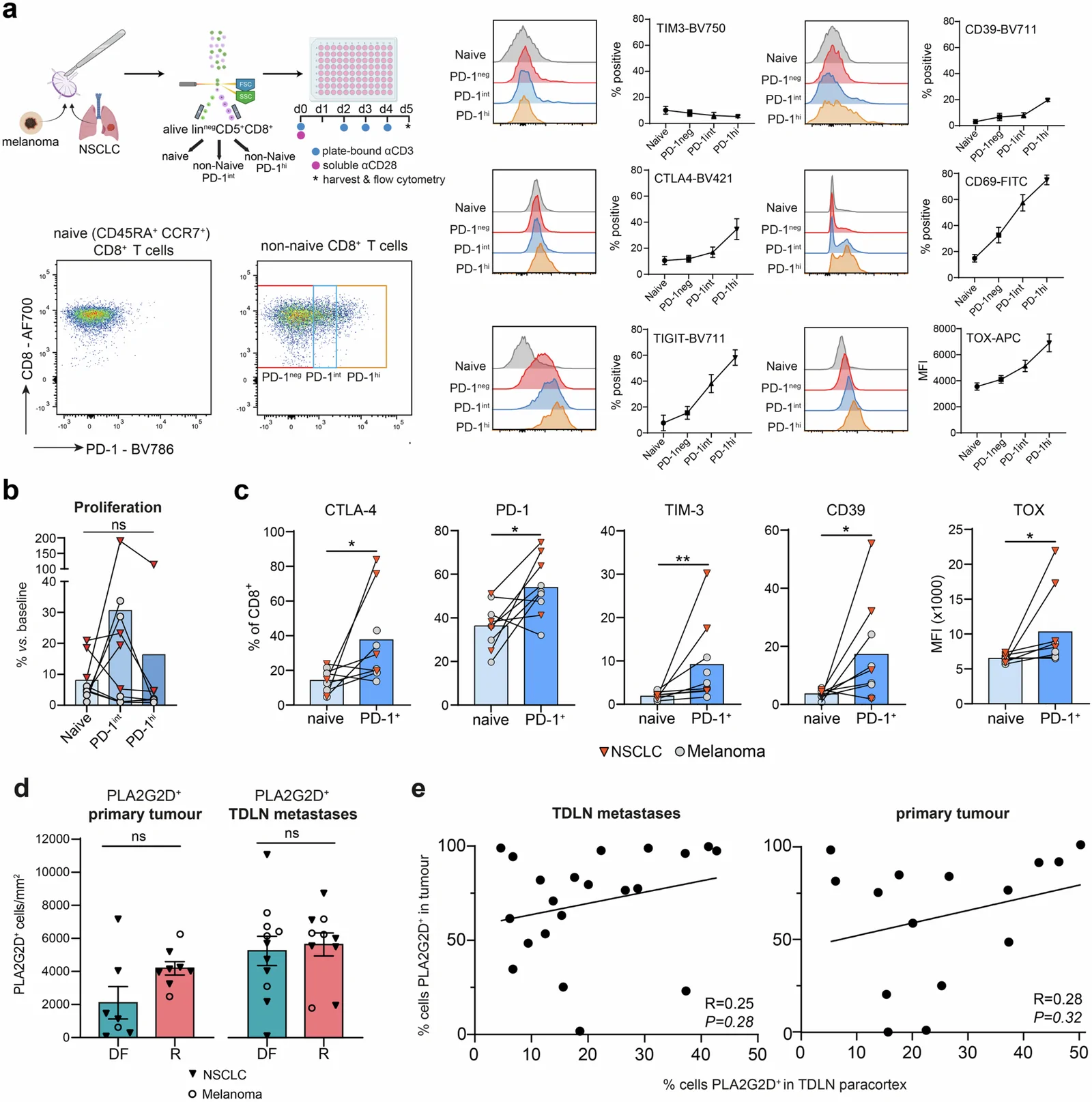
In vitro stimulation of TDLN CD8^+^ T cells and PLA2G2D levels in cancer patient samples. (**a**) Experimental set-up for obtaining FACS-sorted naive, (non-naive) PD-1 intermediate (PD-1^int^) and PD-1 high (PD-1^hi^) CD8^+^ T cells (gating strategy is shown in lower panels) from patient TDLNs, which were cultured using a modified in vitro exhaustion assay consisting of anti-CD3/CD28 stimulation followed by repeated anti-CD3 stimulation and flow-cytometry analysis. On the right side, baseline levels of indicated activation and exhaustion-related markers on the different CD8^+^ T cell subsets in patient TDLNs (n = 4) are shown, including representative flow-cytometry histograms of an individual patient. Created in BioRender; Stadhouders, R. https://BioRender.com/7aouyup (2026). (**b**) Expansion capacity of different T cell subsets following culture (obtained from 4 patients with NSCLC and 6 patients with melanoma), shown as (cell counts after culture/ cell counts at baseline) * 100%. (**c**) Expression of activation/exhaustion-related proteins on sorted and cultured naive CD8^+^ T cells and combined PD−1^int^ and PD-1^hi^ (‘PD-1^+^’) cells from melanoma (n = 6) and NSCLC (n = 4) TDLNs. (**d**) PLA2G2D protein abundance as assessed by IHC plotted as positive cells per mm^2^ of tissue per patient group (DF=disease free, R=recurrence). PLA2G2D was assessed in primary tumour tissue (n = 2 melanoma, n = 5 NSCLC) and TDLN metastases (n = 5 melanoma, n = 6 NSCLC), whenever available from patients with lymph-node metastatic non-small cell lung cancer (NSCLC) or melanoma. (**e**) Correlations (Pearson’s R) were determined between PLA2G2D positivity (shown as the average % of positive cells per ROI) in the TDLN paracortex and the TDLN metastasis (left, n = 21) or primary tumour (right, n = 15) within patients with melanoma or NSCLC. % positive cells were used instead of PLA2G2D^+^ cells per mm^2^ to correct for larger tumour-cell size as compared to immune cells in the TDLN paracortex. *P* values were calculated using a two-sided Wilcoxon rank-sum test (**c**,**d**) or two-way ANOVA with Tukey’s multiple comparisons test (**b**). Data are presented as mean ± s.e.m. (**a**–**d**). Statistical analysis by paired t-test. *P < 0.05, **P < 0.01, ns = non-significant (Exact *P* values in Supplementary Data).

To better characterize the observed myeloid–T cell phenotypes in the TDLN paracortex, we performed a targeted spatial transcriptomics analysis of CD11c^+^ DCs, CD68^+^ macrophages and CD8^+^ T cells co-localizing in paracortex regions from R and DF patients with stage III melanoma (*n* = 5 per group) (Fig. 2a). This approach identified hundreds of differentially expressed genes (DEGs; *P* < 0.05) between R and DF patients for all three cell types (Fig. 2b and Extended Data Figs. 4 and 5 and Supplementary Table 2). Gene set enrichment analysis (GSEA) revealed that DCs in R patients upregulated genes that are strongly linked to mature DCs enriched in immunoregulatory molecules (mRegDCs) marked by the expression of *CCR7*,* LAMP3*,* IL4I1*,* FSCN1* and the IL-4R signalling pathway^21^ (Fig. 2b,c and Extended Data Fig. 4a–d). Macrophages in R patients showed an inflammatory phenotype characterized by a strong induction of interferon-γ (IFNγ)-responsive genes (for example, *CXCL9*,* IRF1* and *STAT1*) and immunosuppressive molecules (for example *IDO1*, *LILRB4* and *CD274* (which encodes PD-L1)) alongside reduced levels of genes related to phagocytosis^22^ (Fig. 2b,c and Extended Data Fig. 4d–g). Paracortex CD8^+^ T cells in R patients showed increased expression of genes encoding co-inhibitory molecules (for example *CTLA4*, *TIGIT* and *PDCD1*), the *CXCL13* chemokine^23^ and its upstream activator *SOX4* (ref. ^24^) and transcription factors such as *TOX*,* IRF4*,* AHR* and *BHLHE40* (refs. ^25–30^ and Extended Data Fig. 5a–d). Immunofluorescence staining confirmed that CXCL13^+^CD8^+^ T cells co-localized with LAMP3^+^CD11c^+^ DCs and CD68^+^ macrophages (Extended Data Fig. 5e). Expression of co-inhibitory receptors, CXCL13 and TOX has been associated with T cell activation but also exhaustion, and TDLNs are known to contain progenitor exhausted T (T_pex_) cells^1,4,31,32^. Consistent with this, CD8^+^ T cells in R patients were enriched for various transcriptional signatures of exhausted and effector memory T cells (Extended Data Fig. 5f,g). IMC analysis showed that proliferating T cells in the paracortex expressed both TCF1 and TOX, indicative of a T_pex_ state^12,14,33–35^ (Extended Data Fig. 5h). Levels of *CXCL13* mRNA in paracortex CD8^+^ T cells correlated with the intratumoural expression of PD-1 and TOX proteins (Extended Data Fig. 5i), suggesting that a T_pex_ state in the TDLN predisposes to T cell exhaustion in the tumour^1,3,14,31^. Indeed, PD-1^+^CD8^+^ T cells (sorted by fluorescence-activated cell sorting; FACS) from patient TDLNs upregulated the exhaustion-associated markers CD39, TIM-3 and TOX more readily than their PD-1^−^ naive counterparts did after prolonged stimulation (Extended Data Fig. 6a–c).

One of the genes that was most strongly upregulated in paracortex myeloid cells from R patients was *PLA2G2D* (Fig. 2d), which encodes a secreted member of the phospholipase A2 family. PLA2G2D has been implicated in the suppression of skin inflammation, chemically induced skin carcinogenesis, lung cancer xenograft growth and coronavirus infection^36–39^. However, its role in anti-tumour immune suppression and TDLN biology remains poorly understood. An analysis of data from The Cancer Genome Atlas (TCGA) revealed robust expression of *PLA2G2D* in melanoma samples, with the highest levels observed in LNs with metastases (Fig. 2e)—in line with previous studies reporting particularly high levels of PLA2G2D in secondary lymphoid organs^36^. Immunohistochemistry (IHC) analysis showed that PLA2G2D protein was expressed in LN metastases, but that it was also prominently expressed in interfollicular regions of the paracortex (Fig. 2f). Notably, an analysis of samples from independent cohorts of patients with metastatic melanoma or non-small cell lung cancer (NSCLC) (Supplementary Tables 3 and 4) confirmed that levels of PLA2G2D were increased in the TDLN paracortex of patients with distant disease recurrence (Fig. 2f). The abundance of PLA2G2D was higher in TDLN metastases than in primary tumour tissue, and intratumoural PLA2G2D levels were not significantly different in R than in DF patients (Extended Data Fig. 6d). Levels of PLA2G2D were not correlated between the TDLN paracortex and tumour tissue within individual patients (Extended Data Fig. 6e). Immunofluorescence staining confirmed the presence of PLA2G2D^+^ macrophages and DCs in both stage II and stage III NSCLC TDLNs, with much lower frequencies observed in primary tumours or LN metastases (Fig. 2g). Of note, most PLA2G2D^+^ myeloid cells in the TDLN paracortex—but not in tumours—resided near PD-1^+^CD8^+^ T cells (Fig. 2h).

Collectively, these results show that the presence of PLA2G2D^+^ myeloid cells in CD8^+^-T-cell-rich paracortex regions of patient TDLNs is associated with poor clinical outcomes.

## PLA2G2D controls tumour growth

**Fig. 3:**
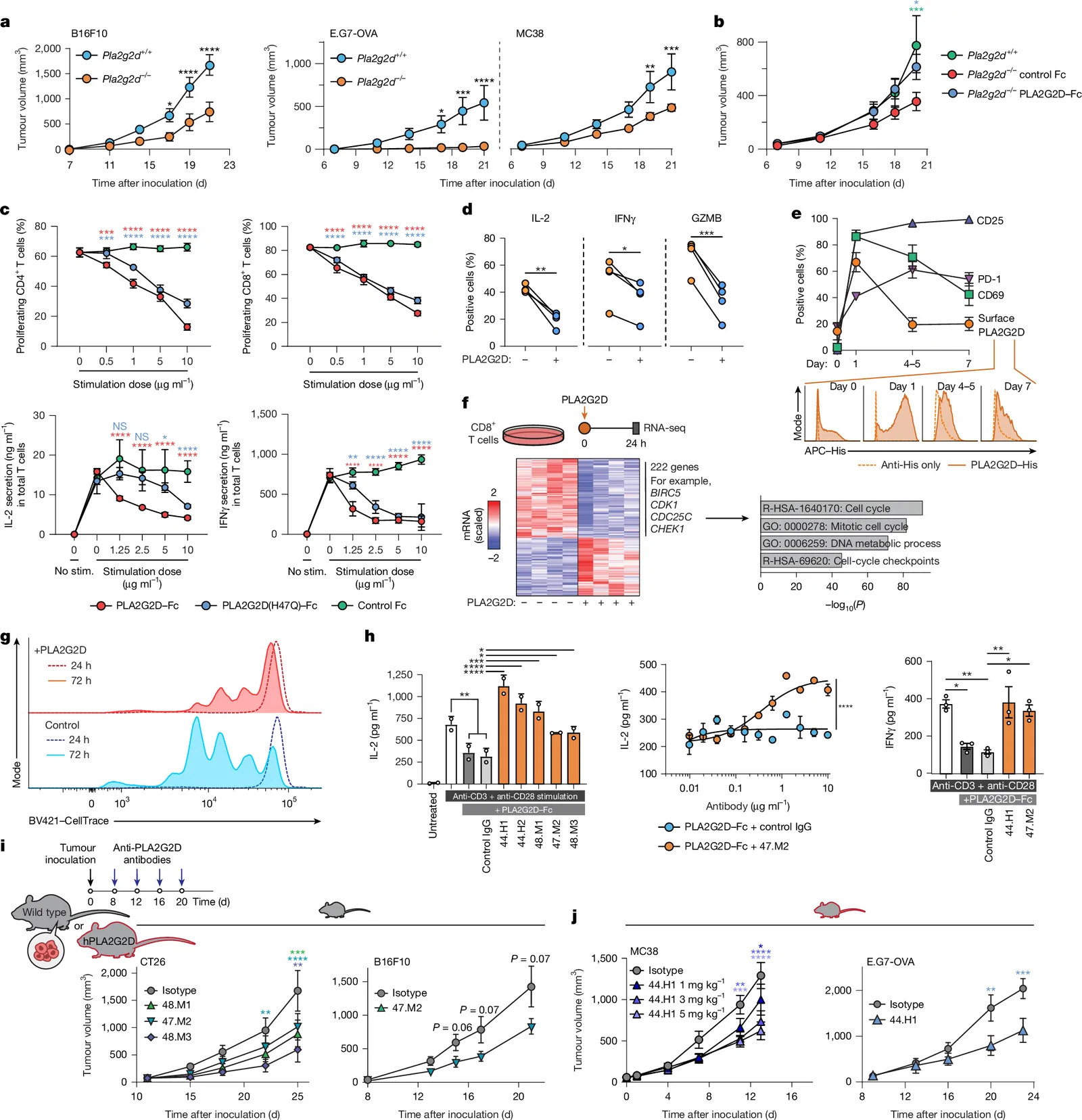
PLA2G2D suppresses T cell activity and controls tumour growth in multiple models of cancer. **a**, Tumour volume measurements in *Pla2g2d*^+/+^ and *Pla2g2d*^*−*/*−*^ mice (*n* = 16 per group) after inoculation with B16F10, E.G7-OVA or MC38 cancer cells. **b**, Tumour volume measured over time in *Pla2g2d*^+/+^ (*n* = 8) and *Pla2g2d*^*−*/*−*^ C57BL/6 mice after inoculation with MC38. *Pla2g2d*^*−*/*−*^ mice received PLA2G2D–Fc (*n* = 8) or control Fc protein (*n* = 7) by intraperitoneal (i.p.) injection. **c**, Proliferation (top; carboxyfluorescein succinimidyl ester (CFSE) dilution assay) and IL-2 or IFNγ secretion (bottom, Meso Scale Discovery immunoassay) were measured in activated (or non-stimulated; no stim.) cultured healthy donor CD4^+^, CD8^+^ or total T cells after exposure to increasing doses of PLA2G2D–Fc, catalytically inactive PLA2G2D(H47Q)–Fc or control Fc proteins (*n* = 3 donors). **d**, Intracellular levels of IL-2, IFNγ and granzyme B (GZMB) in CD8^+^ T cells treated for 72 h with PLA2G2D–Fc (*n* = 4 donors). **e**, Top, surface marker levels of the indicated proteins during CD8^+^ T cell activation in vitro (*n* = 3 donors). Bottom, representative histograms of PLA2G2D surface binding. **f**, DEGs (*P*_adj_ < 0.05) 24 h after exposing human CD8^+^ T cells (*n* = 4 donors) to PLA2G2D protein and TCR (anti-CD3 and CD28) stimulation. The bar graph on the right shows top enriched pathways within upregulated genes (*n* = 222). **g**, Representative CellTrace dilution profiles (measure of proliferation) in activated CD8^+^ T cells that were exposed to PLA2G2D (red) or left untreated (blue) at the indicated time points. **h**, Secretion of IL-2 (*n* = 2 donors) or IFNγ (*n* = 3 donors) in anti-CD3- and anti-CD28-stimulated peripheral blood mononuclear cell (PBMC) cultures exposed to PLA2G2D–Fc in the absence or presence of human-specific (44.H1 or 44.H2) or mouse cross-reactive (48.M1, 47.M2 or 48.M3) anti-PLA2G2D monoclonal antibodies. **i**,**j**, Tumour volume measurements in wild-type (**i**) or humanized *PLA2G2D* (hPLA2G2D; **j**) C57BL/6 mice after inoculation with the indicated cancer cells (isotype, *n* = 9; all others, *n* = 10). Antibodies were administered by i.p. injection. Data are mean ± s.e.m. *P* values by two-way analysis of variance (ANOVA) with Fisher’s least significant difference (LSD) (**a**–**c**,**i**,**j**), two-way ANOVA with Šidák correction for multiple testing (**h**) or two-sided paired *t*-test (**d**). *P < 0.05, **P < 0.01, ***P < 0.001, ****P < 0.0001 (exact *P* values in Supplementary Data). Source data

**Extended Data Fig. 7:**
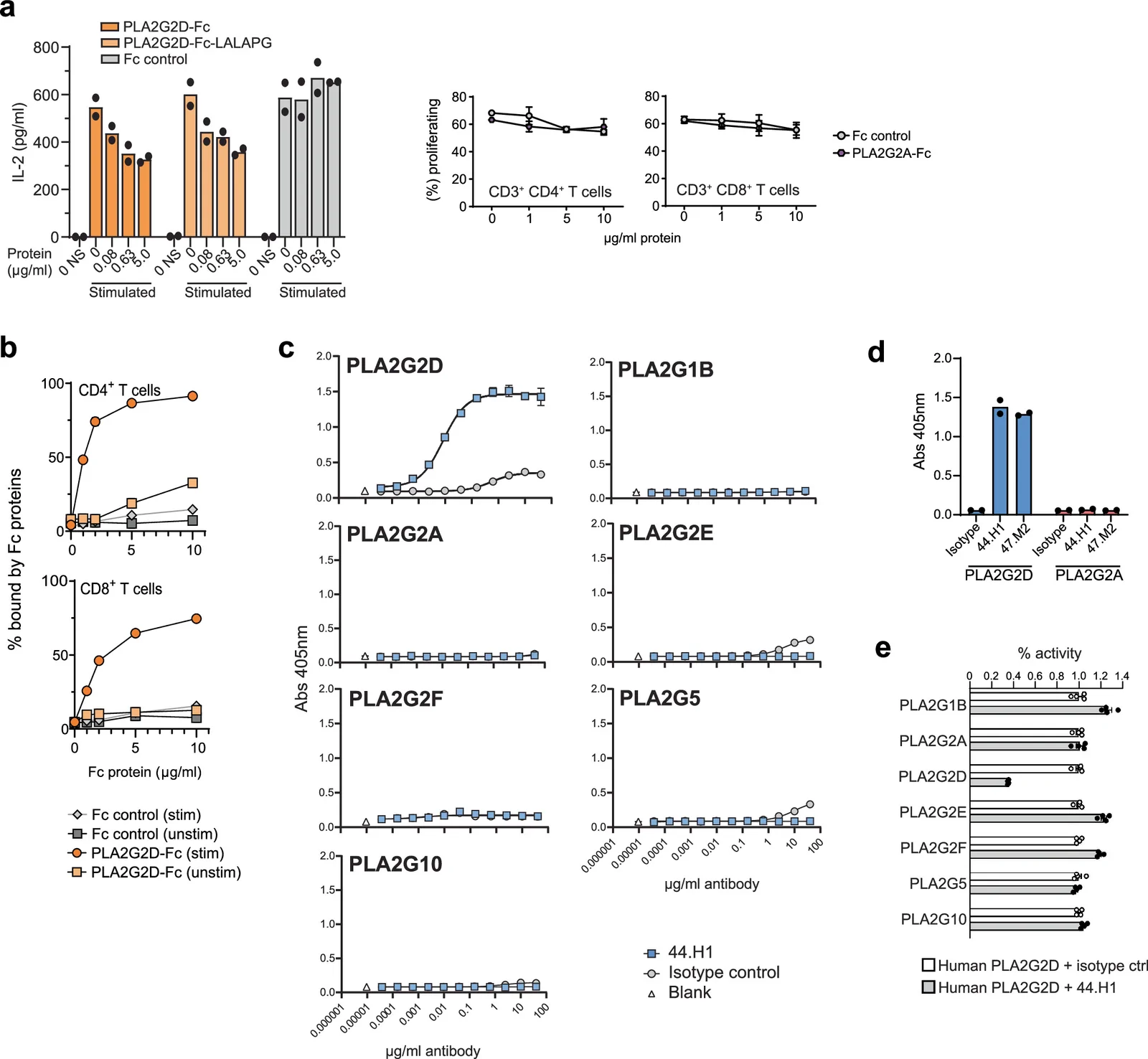
Development of tools to experimentally manipulate PLA2G2D activity. (**a**) Left: IL-2 secretion (MSD immunoassay) was measured over time in cultured T cells isolated from healthy donor PBMCs (n = 2) following treatment with either PLA2G2D–Fc, PLA2G2D–Fc-LALAPG, or control Fc proteins. Right: Proliferation (CFSE dilution assay) were measured over time in cultured CD4^+^ and CD8^+^ T cells isolated from healthy donor PBMCs following treatment with either PLA2G2A-Fc or control Fc proteins. (**b**) Representative flow-cytometry results from >10 independent experiments showing PLA2G2D binding to activated CD3^+^CD4^+^ T cells and CD3^+^CD8^+^ T cells in PBMC cultures 48 h after stimulation with anti-CD3 and anti-CD28 antibodies. (**c**,**d**) ELISA assays (n = 2 independent experiments) measuring binding of indicated sPLA2 family members to increasing amounts of 44.H1 or 47.M2 anti-PLA2G2D antibodies (or an isotype control antibody). (**e**) Enzymatic assay (n = 4 independent experiments) detecting release of the fatty acid docosahexaenoic acid (DHA) to determine the specificity of anti-PLA2G2D antibodies (44.H1 is shown as a representative example) to inhibit the catalytic function of various sPLA2 family members. Activity is normalized against isotype control IgG treatment for each sPLA2. Data are presented as mean ± s.e.m. (**a**–**e**).

To investigate whether PLA2G2D controls cancer progression, we monitored the growth of various tumours, including MC38 colorectal carcinoma and B16F10 melanoma, in wild-type and *Pla2g2d*-deficient (*Pla2g2d*^−/−^) mice. Tumour growth was substantially diminished in *Pla2g2d*^−/−^ mice and could be rescued by treating mice with PLA2G2D–Fc protein (Fig. 3a,b). In line with previous work using cultured mouse T cells^40^, incubating human CD4^+^ or CD8^+^ T cells isolated from healthy donor peripheral blood with PLA2G2D–Fc protein potently suppressed proliferation, effector cytokine production and granzyme B production after T cell receptor (TCR) stimulation in vitro (Fig. 3c,d). This was not due to any potential ectopic Fc-effector function from the PLA2G2D–Fc protein, because a variant containing a LALAPG mutation in the Fc domain (preventing complement and Fc-receptor binding) still maintained suppressive activity (Extended Data Fig. 7a). In addition, PLA2G2D’s closest homologue, PLA2G2A, did not suppress T cell proliferation (Extended Data Fig. 7a). Notably, PLA2G2D’s inhibition of T cells was mostly independent of its enzymatic activity, because catalytically inactive PLA2G2D(H47Q)–Fc also inhibited T cell activation (Fig. 3c). PLA2G2D might therefore signal through an unknown receptor on T cells. Secondary antibody staining revealed that PLA2G2D bound to most T cells after stimulation in a dose-dependent manner (Extended Data Fig. 7b). Compared with the kinetics of known activation-induced T cell surface markers (CD25, CD69 and PD-1), PLA2G2D surface binding decreased back to baseline levels more rapidly (Fig. 3e). RNA sequencing (RNA-seq) at the peak of PLA2G2D binding (that is, after 24 h) revealed a marked downregulation of cell-cycle-related genes and pathways (Fig. 3f), which led to a strong reduction in T cell proliferation (Fig. 3g). These data show that PLA2G2D can directly suppress early T cell expansion and effector function.

To target PLA2G2D activity in vivo and treat established tumours, we generated human-specific (clone 44.H) and mouse-reactive (clones 47M and 48M) antibodies that blocked the function of PLA2G2D. Neutralizing capacity was determined on the basis of the antibody’s ability to reverse the PLA2G2D-mediated suppression of IL-2 and IFNγ secretion in a T cell activation assay (Fig. 3h). Antibodies were specific to PLA2G2D and no other secretory PLA2 isoforms (Extended Data Fig. 7c–e). Administering function-blocking anti-PLA2G2D antibodies to tumour-bearing mice resulted in inhibition of tumour growth in various cancer models, both in wild-type mice treated with mouse PLA2G2D-reactive antibodies (Fig. 3i) and in mice genetically engineered to express the human *PLA2G2D* gene to allow for targeting with human-specific PLA2G2D antibodies (Fig. 3j).

## In vivo source and role of PLA2G2D

**Fig. 4:**
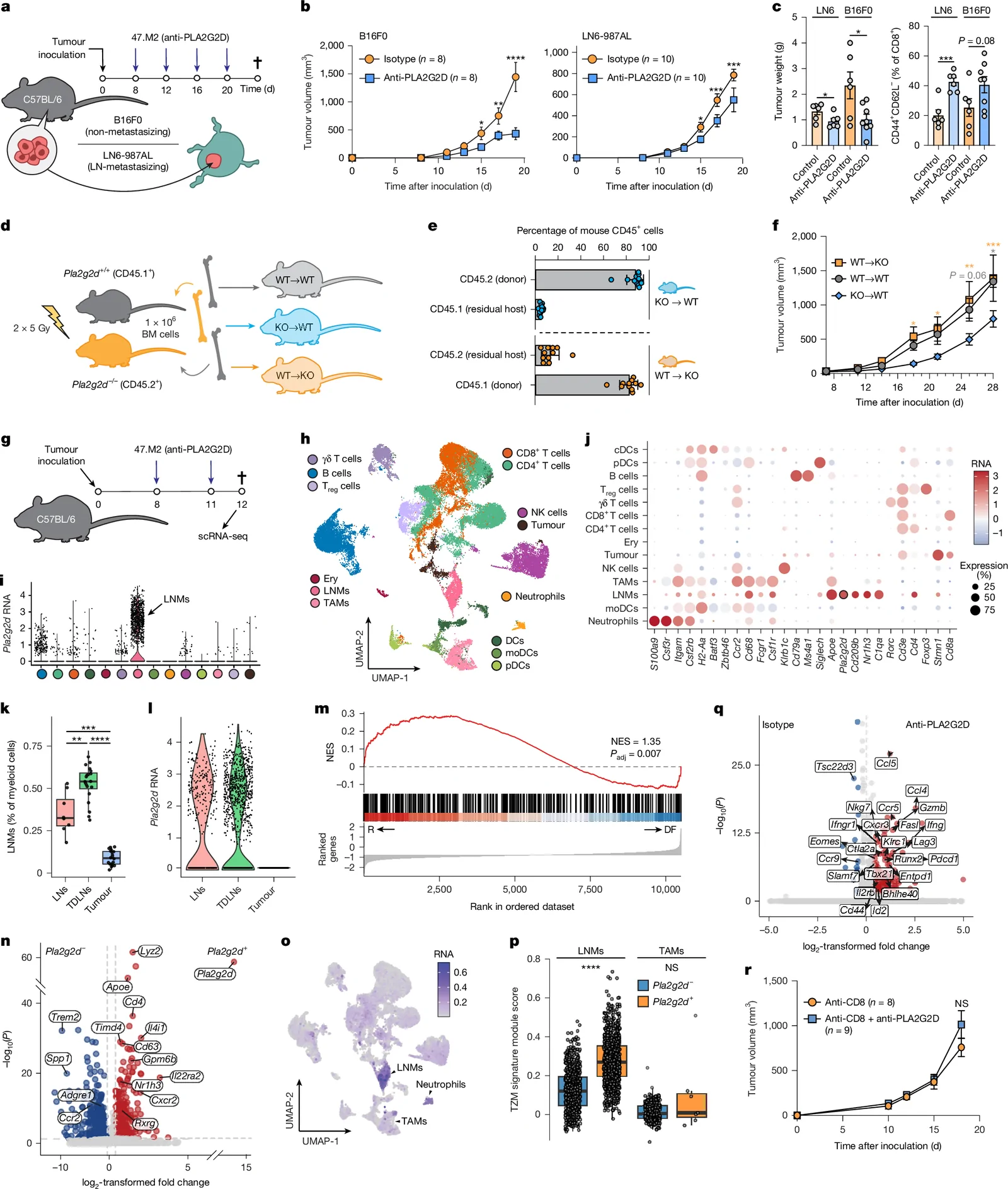
TDLN macrophages are the early source of PLA2G2D in vivo. **a**, Experimental set-up. **b**, Tumour volume measurements in isotype or anti-PLA2G2D antibody-treated mice bearing B16F0 (*n* = 8 per group) or LN6-987AL (*n* = 10 per group) tumours. **c**, Tumour weight (left) and abundance of intratumoural effector memory CD8^+^ T cells (right) (*n* = 6 mice; *n* = 8 for anti-PLA2G2D B16F0). **d**, Generation of bone marrow (BM) chimeras. KO, knockout; WT, wild type. **e**, Donor chimerism analysis (WT→KO, *n* = 11; KO→WT, *n* = 12). **f**, Tumour volumes in WT→KO (*n* = 7), WT→WT (*n* = 4) or KO→WT (*n* = 10) mice inoculated with MC38 cancer cells. **g**, Strategy for single-cell RNA-seq (scRNA-seq) of tumours (*n* = 20), TDLNs (*n* = 20) and non-draining LNs (*n* = 4) 12 days after inoculation of wild-type mice with B16F0 or LN6-987AL melanoma cells. Mice were treated twice with anti-PLA2G2D antibody (47.M2). **h**, Annotated UMAP representation of single-cell transcriptomes. Ery, erythrocytes; moDCs, monocyte-derived dendritic cells; NK cells, natural killer cells; pDCs, plasmacytoid dendritic cells; TAMs, tumour-associated macrophages. **i**, *Pla2g2d* RNA levels (normalized read counts) across cell-type clusters. **j**, Balloon plot depicting expression levels of cell identity markers and *Pla2g2d*. **k**, Abundance of LNMs (% of all myeloid cells). **l**, Normalized read counts of *Pla2g2d* RNA in LNMs in the indicated tissues. **m**, GSEA enrichment of LNM signature genes in DEGs from macrophages from the TDLN paracortex of patients with melanoma (see Fig. 2b). **n**, DEGs (*P* < 0.05) between LNMs with detectable (*Pla2g2d*^+^) or undetectable (*Pla2g2d*^−^) *Pla2g2d* RNA. **o**, TZM gene signature module score plotted onto UMAP. **p**, TZM gene module activity across *Pla2g2d*^+^ or *Pla2g2d*^−^ LNMs and TAMs. **q**, DEGs (*P* < 0.05) between CD8^+^ T cells from isotype- or anti-PLA2G2D antibody-treated mice. **r**, LN6-987AL tumour volume in isotype- or anti-PLA2G2D (47.M2) antibody-treated mice after antibody-mediated CD8^+^ T cell depletion (*n* = 8 or 9). Line and bar graphs denote mean ± s.e.m. *P* values by two-way ANOVA with Fisher’s LSD (**b**,**f**,**r**), unpaired two-sided t-test (**c**,**k**,**l**) or two-sided Wilcoxon rank-sum test (**n**,**p**,**q**). *P < 0.05, **P < 0.01, ***P < 0.001, ****P < 0.0001 (exact *P* values in Supplementary Data). Source data

To investigate whether anti-PLA2G2D antibodies also suppressed metastatic tumours, we injected wild-type mice with B16F0-derived LN6-987AL melanoma cells, which metastasize to LNs^19^—resembling our stage III patient population (Fig. 4a). In both non-metastatic (parental B16F0) and metastatic (LN6-987AL) melanoma models, tumour progression was delayed, and was accompanied by increased proportions of tumour-infiltrating CD62L^−^CD44^+^ effector memory CD8^+^ T cells (Fig. 4b,c). These results suggest that the observed therapeutic efficacy of PLA2G2D inhibition involves boosting CD8^+^ T cell responses.

To determine more precisely which cells produce PLA2G2D in vivo and mediate its immunosuppressive effects, we first generated bone-marrow chimeras. Using CD45.1 and CD45.2 congenic mouse strains, we successfully engrafted lethally irradiated wild-type mice with *Pla2g2d*^−/−^ bone marrow and vice versa (Fig. 4d,e). Assessment of MC38 tumour growth in these mice revealed that cancer progression was reduced only in wild-type mice with a PLA2G2D-deficient haematopoietic system (Fig. 4f). Thus, PLA2G2D-mediated impairment of anti-tumour responses is mediated by adult haematopoietic-stem-cell-derived migratory cells and not by stromal cells or long-term tissue-resident immune cells.

**Extended Data Fig. 8:**
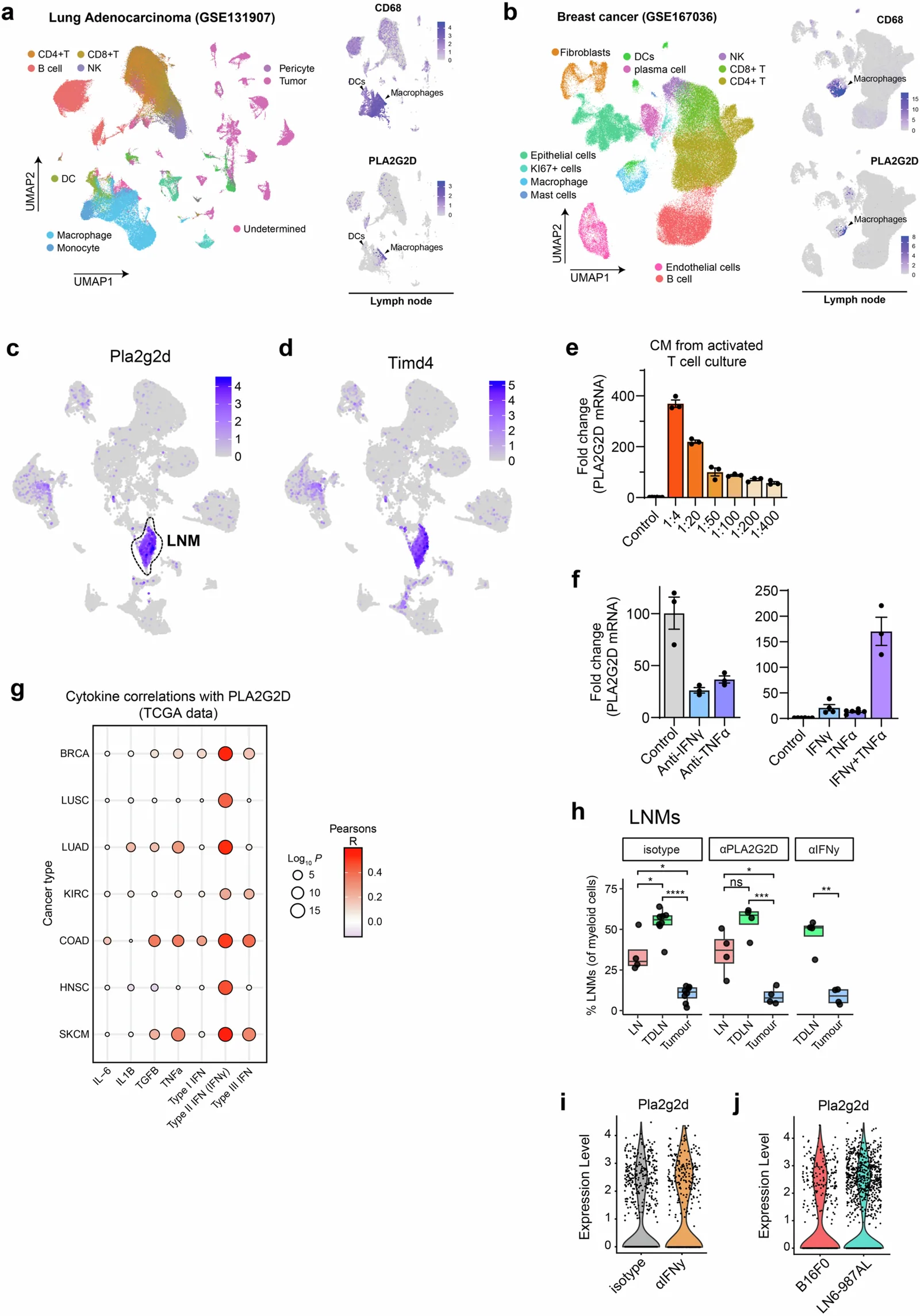
PLA2G2D expression in LN cells and characterization of PLA2G2D-inducing signals. (**a**,**b**) Analysis of published single-cell transcriptome datasets from lung adenocarcinoma (**a**) and breast cancer (**b**) TDLNs, with UMAP showing cellular diversity within the full datasets. CD68 expression is depicted to highlight the LNM population, in which the bulk of the PLA2G2D-expressing cells reside. (**c**,**d**) Scaled *Pla2g2d* (**c**) and *Timd4* (**d**) RNA levels projected on the single-cell RNA-seq UMAP. (**e**) qRT–PCR analysis of *PLA2G2D* mRNA in human monocyte-derived macrophages (MDMs) 2 days after the addition of various dilutions of conditioned medium (CM) from T cell cultures activated with anti-CD3 and anti-CD28 antibodies (n = 3 per group). (**f**) Left: *PLA2G2D* mRNA expression measured by qRT–PCR in MDMs 2 days after exposure to CM from activated T cells in the presence of anti-IFNγ (15 µg/ml) or anti-TNF (15 µg/ml) neutralizing antibodies (n = 3 per group). Right: *PLA2G2D* mRNA expression measured by qRT–PCR in MDMs 2 days following treatment with recombinant IFNγ (60 ng/ml; n = 4), TNF (60 ng/ml; n = 6), or IFNγ (60 ng/ml) plus TNF (60 ng/ml) (n = 3). (**g**) Log_10_ P values and Pearson correlation coefficients (R) between expression levels of genes encoding different cytokines and PLA2G2D in various tumour types (data obtained from TCGA). In case of TGFβ (TGFB), type I and III interferons (IFNs), the isoform with highest R was plotted. IL-6=interleukin 6, IL1B= interleukin 1β, TGFB= transforming growth factor β, TNF=tumour necrosis factor α, BRCA=breast invasive carcinoma, LUSC=lung squamous cell carcinoma, LUAD= lung adenocarcinoma, KIRC: kidney clear cell carcinoma, COAD= colon adenocarcinoma, HNSC= Head and neck squamous cell carcinoma, SKCM= cutaneous melanoma. (**h**) Proportions of lymph-node macrophages (LNMs) of total myeloid cells per treatment group and tissue origin. Group sizes (mice): isotype; LN = 4, TDLN = 8, Tumour=7; anti-PLA2G2D; LN = 4, TDLN = 4, Tumour=4; aIFNγ; TDLN = 4, Tumour=4. (**i**) Violin plot of *Pla2g2d* RNA levels in untreated and anti-IFNγ treated mice. (**j**) Violin plot of *Pla2g2d* RNA levels in B16F0 and LN6-987AL inoculated mice. Statistical analysis was performed using a two-sided unpaired t-test (**h**). *P < 0.05, **P < 0.01, ***P < 0.001, ****P < 0.0001. (Exact *P* values in Supplementary Data).

We next used single-cell transcriptomics to identify *Pla2g2d*-expressing cell populations in vivo. Non-draining LNs, TDLNs and primary tumours from mice bearing LN-metastatic LN6-987AL or non-metastatic parental B16F0 melanoma were analysed 12 days after tumour inoculation (Fig. 4g,h). Notably, robust *Pla2g2d* expression was confined to a single cell population: a subset of macrophages that was highly abundant in TDLNs but undetectable in primary tumours (Fig. 4i,j), with *Pla2g2d* emerging as a top identity-defining gene of this LN macrophage (LNM) subset (Fig. 4j–l). LNMs showed a strong transcriptional resemblance to the paracortex macrophage phenotype detected in patients with melanoma whose disease had recurred (Fig. 4m). Analyses of published single-cell RNA-seq data from patients with lung adenocarcinoma^41^ or breast cancer^42^ confirmed that LNMs are the primary *Pla2g2d*-expressing cells in vivo (Extended Data Fig. 8a,b). Because *Pla2g2d* was heterogeneously expressed across LNMs, we compared *Pla2g2d*^+^ and* Pla2g2d*^−^ LNMs, and observed a strong upregulation in *Pla2g2d*^+^ LNMs of numerous genes that have previously been shown to define T-cell-zone macrophages^43^ (TZMs; Fig. 4n–p and Extended Data Fig. 8c,d), including *Timd4*,* Il22ra2*, *Rxrg* and *Pla2g2d* itself. By contrast, typical markers of other LNM subsets, such as *Adgre1* (encoding F4/80), showed an opposite trend (Fig. 4n and Extended Data Fig. 8c,d), indicating that PLA2G2D-producing LNMs have a TZM phenotype. These observations fit with our identification of *PLA2G2D*-expressing myeloid cells in the TDLN paracortex, which is the anatomical region that corresponds to the T cell zone^43,44^.

To identify factors that induce PLA2G2D, we exposed human monocyte-derived macrophages (MDMs) to activated T cell culture supernatant or inflammatory cytokines (IFNγ and TNF) in vitro, which resulted in a robust induction of *PLA2G2D* expression (Extended Data Fig. 8e,f). Analysis of TCGA data revealed strong correlations between the levels of *PLA2G2D* RNA and of *IFNG* and *TNF* RNA across tumour types (Extended Data Fig. 8g), suggesting that T-cell-derived inflammatory cytokines have a role in driving the production of PLA2G2D by myeloid cells. In the LN6-987AL model, however, antibody-mediated neutralization of IFNγ did not significantly reduce LNM frequencies or *Pla2g2d* expression levels, indicating that additional factors contribute to PLA2G2D induction in vivo (Extended Data Fig. 8h,i). Of note, *Pla2g2d* induction in LNMs was similar in the LN6-987AL and B16F0 models (Extended Data Fig. 8j). Thus, PLA2G2D is produced mainly by macrophages in the LN T cell zone, where it can be targeted therapeutically to inhibit tumour progression.

## Dual targeting of PLA2G2D and PD-1

In melanoma tumour models, treatment with anti-PLA2G2D antibodies induced the expression of genes encoding effector molecules, such as chemokines, granzymes and cytokines, in CD8^+^ T cells, but also induced the expression of genes that encode co-inhibitory receptors (for example, *Pdcd1*,* Lag3* and *Entpd1*; Fig. 4q). This indicates that anti-tumour activity increases in the face of imminent checkpoint activation, which could offer opportunities for combination therapies with existing ICIs.

**Fig. 5:**
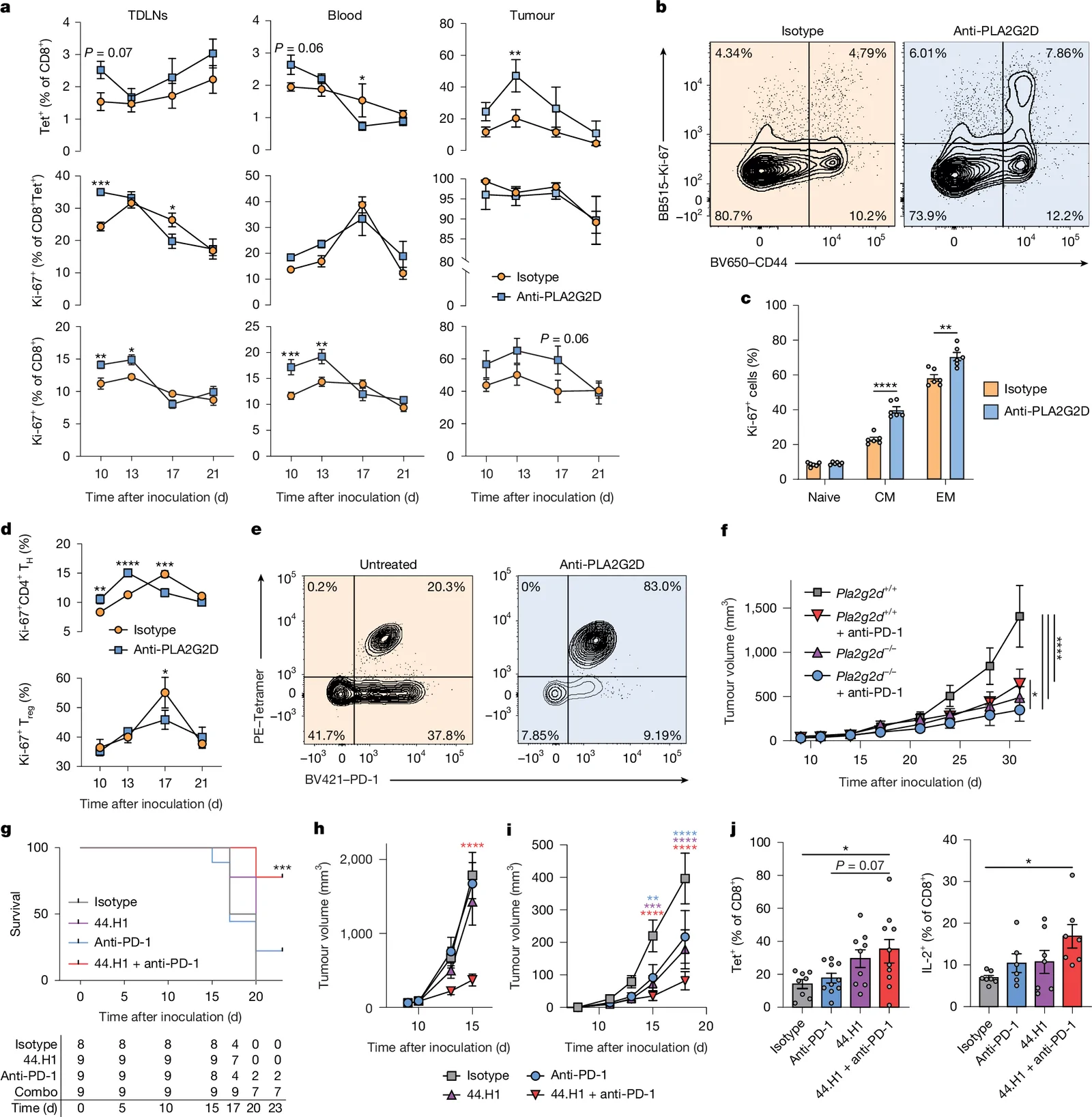
Dual targeting of PLA2G2D and PD-1 checkpoints improves anti-tumour immunity. **a**, Immune monitoring in isotype- or anti-PLA2G2D antibody-treated mice bearing LN6-987AL tumours (see Fig. 4a). Graphs show percentages of TRP2 tetramer-positive (Tet^+^) or Ki-67^+^ (Tet^+^ or total) CD8^+^ T cells across the indicated tissues (*n* = 6 per group). **b**, Representative flow-cytometry analysis showing Ki-67 versus CD44 signals on TDLN CD8^+^ T cells from isotype- or anti-PLA2G2D antibody-treated mice (day 10). **c**, Percentages of Ki-67^+^ naive, central memory (CM) and effector memory (EM) CD8^+^ T cells in blood from isotype- or anti-PLA2G2D antibody-treated mice (day 13; *n* = 6 per group). **d**, Percentages of Ki-67^+^CD4^+^ T helper (T_H_) cells (top) and T_reg_ cells (bottom) in blood from isotype- or anti-PLA2G2D antibody-treated mice (*n* = 6 per group). **e**, Representative flow-cytometry analysis showing TRP2 tetramer versus PD-1 signals on tumour CD8^+^ T cells from isotype- or anti-PLA2G2D antibody-treated mice (day 13). **f**, Tumour volumes in *Pla2g2d*^+/+^ and *Pla2g2d*^−/−^ mice after inoculation with MC38 tumour cells and treatment with isotype control (*n* = 9) or anti-PD-1 (*n* = 10) antibodies. **g**, Kaplan–Meier survival curve of humanized *PLA2G2D* mice inoculated with B16F10 melanoma cells and treated with isotype control (grey; *n* = 8), anti-PLA2G2D (44.H1; purple; *n* = 9), anti-PD-1 (blue; *n* = 9) or anti-PLA2G2D (44.H1) and anti-PD-1 (combo; red; *n* = 9) antibodies. **h**,**i**, Tumour volumes in humanized *PLA2G2D* mice after inoculation with B16F10 (**h**; isotype, *n* = 13; other, *n* = 16) or LN6-987AL (**i**; isotype, *n* = 9; other, *n* = 10) melanoma cells. **j**, Percentages of tumour-specific (Tet^+^) CD8^+^ T cells (left) and IL-2^+^ CD8^+^ T cells (right) in tumours from mice treated with isotype control (*n* = 9), anti-PLA2G2D (44.H1), anti-PD-1 or anti-PLA2G2D (44.H1) and anti-PD-1 antibodies (*n* = 10 per group). Line and bar graphs denote mean ± s.e.m. *P* values by two-way ANOVA with Fisher’s LSD (**a**,**d**,**f**,**i**) or Tukey’s multiple comparisons test (**h**), two-sided unpaired *t*-test (**c**), one-way ANOVA with Tukey’s multiple comparisons test (**j**) or log-rank test (**g**). *P < 0.05, **P < 0.01, ***P < 0.001, ****P < 0.0001 (exact *P* values in Supplementary Data). Source data

**Extended Data Fig. 9:**
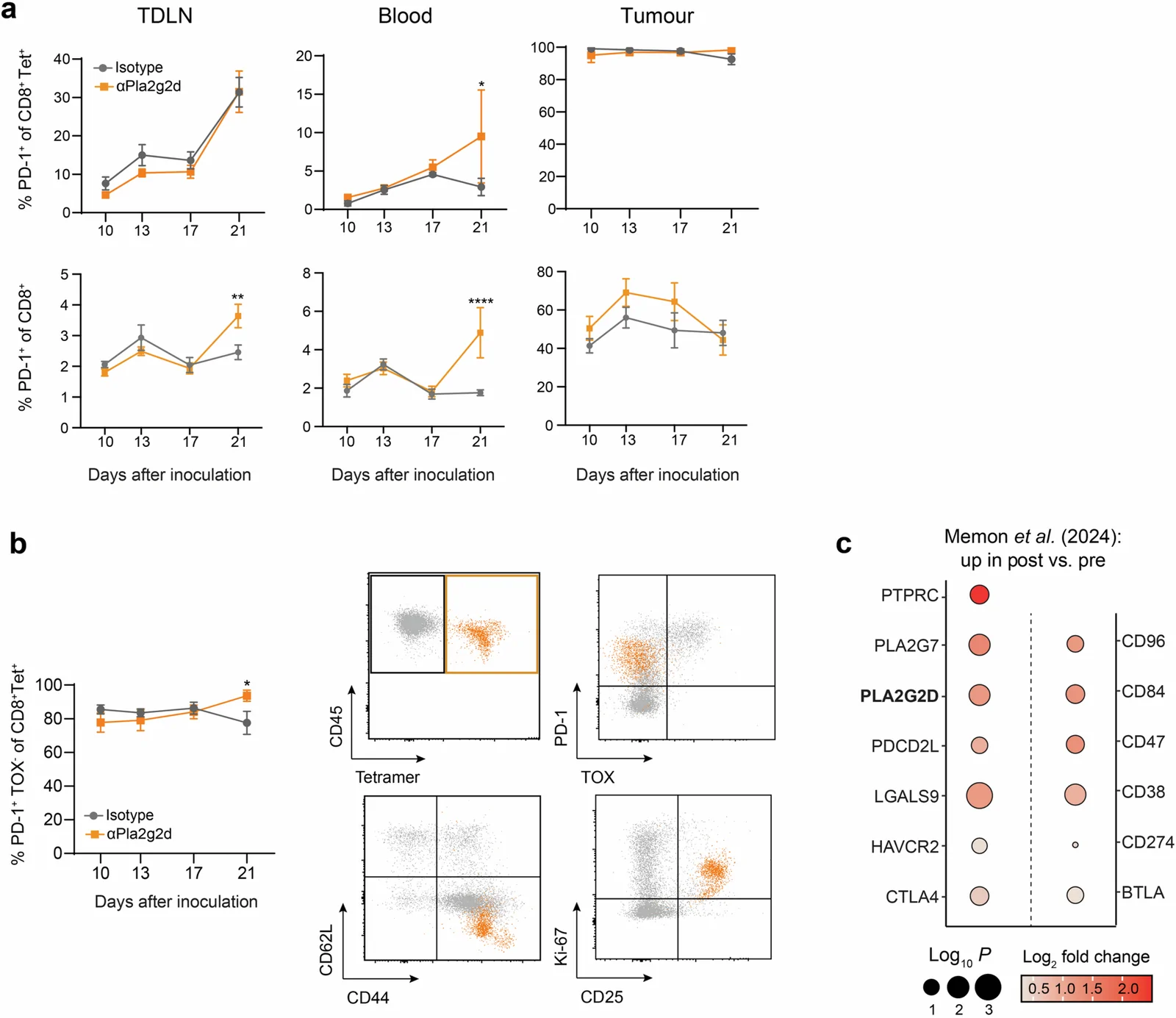
PD-1 levels on T cells following PLA2G2D inhibition. (**a**) Longitudinal immune monitoring in isotype (n = 8) or anti-PLA2G2D (n = 5) antibody-treated mice inoculated with LN6-987AL LN-metastatic melanoma (experimental set-up as in Fig. 4a). Depicted are percentages of PD-1^+^ cells among TRP2 tetramer (‘Tet’, denotes tumour-specific cells) positive CD8^+^ T cells (top) or all CD8^+^ T cells (bottom) – measured across indicated tissues. The *x* axis denotes days after tumour inoculation. (**b**) Same experimental set-up as in **a**. Left: Line graph depicts percentages of PD-1^+^TOX^*−*^ cells among CD8^+^Tet^+^ T cells in the tumour, *x* axis denotes days after tumour inoculation. Right: scatter plots showing protein levels of the indicated markers using flow cytometry. TRP2 tetramers recognize tumour-specific T cells, which are highlighted in orange across the plots. (**c**) Dot plot depicts log_2_ fold changes in RNA expression of the indicated genes comparing NSCLC biopsies before anti-PD-1 therapy (pre) and after (post) onset of acquired therapy resistance as reported by Memon et al.^12^. Data are presented as mean ± s.e.m. (**a**,**b**). *P* values were calculated using a two-way ANOVA with Fisher’s LSD (**a**,**b**) or a two-sided Wilcoxon rank-sum test (**c**). *P < 0.05, **P < 0.01, ****P < 0.0001. (Exact *P* values in Supplementary Data). Source data

**Extended Data Fig. 10:**
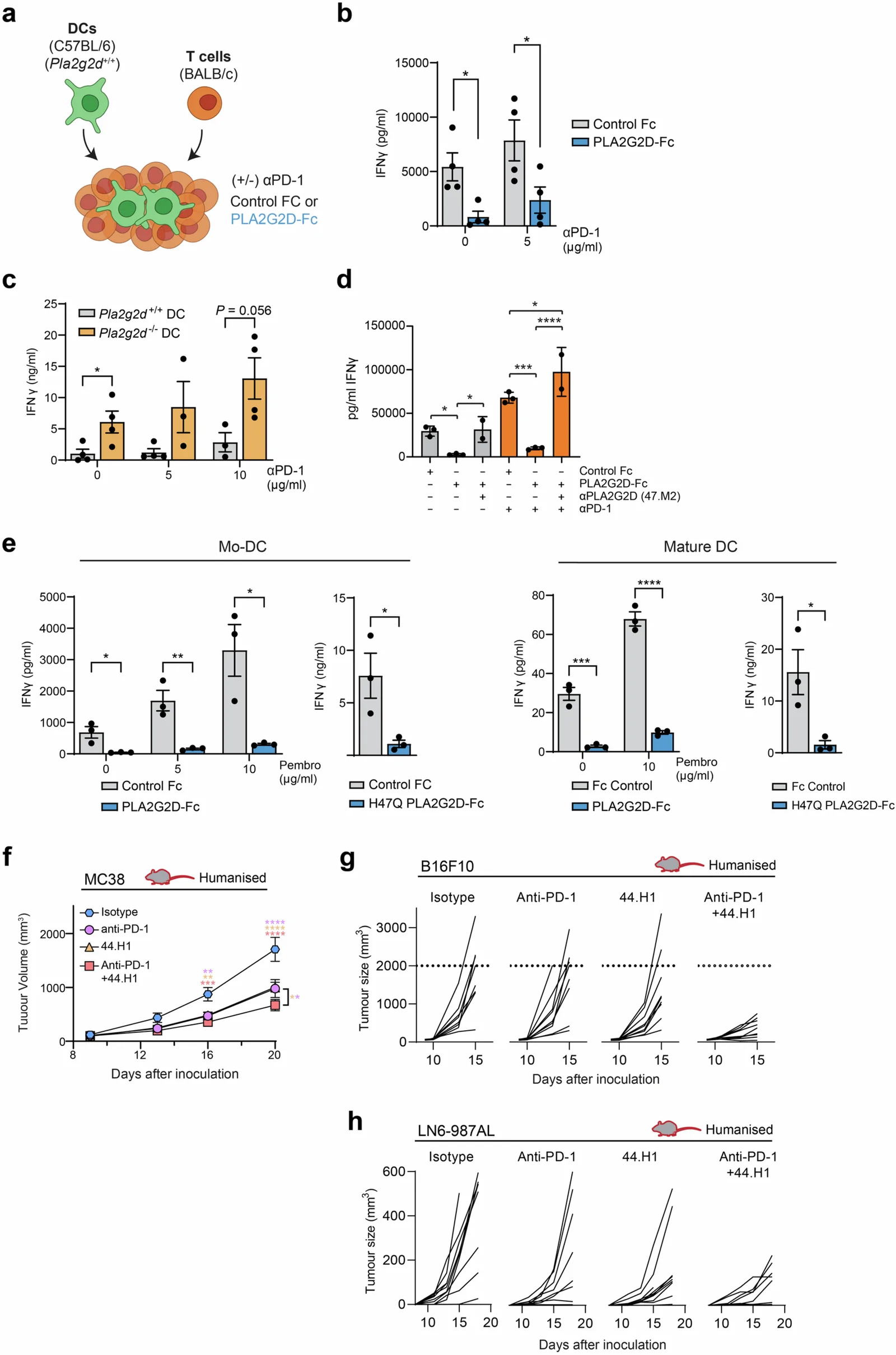
Non-redundant roles for PLA2G2D and PD-1. (**a**) Experimental outline of mouse DC-mediated allogeneic T cell activation to study the effect of PLA2G2D–Fc. DCs and T cells were derived from different mouse strains. (**b**) IFNγ secretion (MSD immunoassay) in culture supernatant (set-up as in **a**) after exposure to control Fc or PLA2G2D–Fc or proteins in the absence or presence of anti-PD-1 antibodies (n = 4 per group). (**c**) IFNγ secretion (MSD immunoassay) after allogeneic DC-mediated T cell activation using DCs derived from *Pla2g2d*^+/+^ or *Pla2g2d*^−/−^ mice (set-up as in **a**, n = 4 per group). (**d**) IFNγ production (MSD immunoassay) from pembrolizumab (anti-PD-1)-treated allogeneic human monocyte-derived DC and T cell co-cultures using normally differentiated DCs exposed to PLA2G2D–Fc or control Fc proteins as well as anti-PLA2G2D antibodies (clone 47.M2) during the allogeneic culture (n = 2–3 per group). (**e**) IFNγ production (MSD immunoassay) from pembrolizumab-treated allogeneic human monocyte-derived DC and T cell co-cultures using mature DCs differentiated in the presence of PLA2G2D, H47Q PLA2G2D, or Fc control proteins (n = 3 per group. (**f**) Tumour growth curves depicting tumour volume (mm^3^) in humanized-*PLA2G2D* C57BL/6 mice (n = 12 per group) following inoculation with MC38 tumour cells and treatment with isotype control, anti-PLA2G2D (44.H1), anti-PD-1, or anti-PLA2G2D and anti-PD-1 antibodies. (**g**,**h**) Individual tumour growth curves for the experiments shown in Fig. 5h,i. Data are presented as mean ± s.e.m. (**b**–**f**). P values were calculated using an unpaired t-test (**b**,**c**,**e**) or a two-way ANOVA with Fisher’s LSD (**d**,**f**). *P < 0.05, **P < 0.01, ***P < 0.001, ****P < 0.0001 (Exact *P* values in Supplementary Data). Source data

Given that PLA2G2D directly affected CD8^+^ T cell phenotypes in vitro and in vivo, we set out to further characterize the effects of PLA2G2D blockade on anti-tumour CD8^+^ T cell responses. Depleting CD8^+^ T cells in the LN6-987AL model abrogated the treatment benefit, indicating that CD8^+^ T cells are indeed necessary for anti-PLA2G2D efficacy in vivo (Fig. 4r). Longitudinal monitoring of CD8^+^ T cell responses across the blood and tissues of melanoma-bearing mice revealed that treatment with anti-PLA2G2D antibodies transiently increased the abundance of Ki-67^+^CD8^+^ T cells—including tumour-specific (TRP2-tetramer positive) and memory T cells—in the TDLNs and blood at early time points (Fig. 5a–c). Notably, whereas Ki-67^+^CD4^+^ T cells were also increased in the blood, inhibition of PLA2G2D did not induce the activation of T_reg_ cells (Fig. 5d). Tumour-specific CD8^+^ T cells subsequently increased substantially in the primary tumour (day 13), coinciding with delayed cancer progression, although these numbers returned to levels observed in untreated mice at later time points (Fig. 5a). This inability to sustain the improved anti-tumour immunity induced by anti-PLA2G2D treatment was accompanied by high levels of PD-1^+^CD8^+^ T cells in the blood, TDLNs and tumour (Fig. 5e and Extended Data Fig. 9a). Of note, tetramer-positive CD8^+^ T cells in tumours remained highly proliferative and did not upregulate TOX, suggesting that these cells have not yet adopted an exhausted phenotype (Extended Data Fig. 9b). Moreover, *PLA2G2D* was prominently upregulated in NSCLC tumours that acquired resistance to anti-PD-1 therapy^16^ (Extended Data Fig. 9c), providing further rationale for combining PLA2G2D blockade with PD-1 ICI. Indeed, mixed lymphocyte reactions using mouse or human T cells and DCs showed that the induction of IFNγ secretion by anti-PD-1 blocking antibodies was suppressed by PLA2G2D, with genetic deletion or antibody-mediated inhibition of PLA2G2D ameliorating the suppression of cytokine release (Extended Data Fig. 10a–e). Combining anti-PD-1 inhibition with genetic or antibody-mediated inactivation of PLA2G2D in the immunogenic MC38 tumour model revealed that combined inhibition suppressed tumour growth beyond the efficacy achieved with monotherapy (Fig. 5f and Extended Data Fig. 10f). Notably, treating *PLA2G2D*-humanized mice bearing ICI-insensitive B16F10 or metastatic LN6-987AL tumours with human-specific anti-PLA2G2D antibodies either sensitized tumours to (B16F10) or further improved the efficacy of (LN6-987AL) anti-PD-1 ICI (Fig. 5g–i and Extended Data Fig. 10g,h). This improved efficacy with combination therapy was accompanied by increased frequencies of tumour-specific and IL-2-producing CD8^+^ T cells in the TME (Fig. 5j). Thus, PLA2G2D and PD-1 represent non-redundant immune checkpoints that could be exploited in combination immunotherapy strategies.

## Discussion

Here we show that the cellular composition of TDLNs differs between patients with melanoma who have a poor prognosis after surgery and those who have a favourable one. Specifically, we found that TDLNs from patients with metastatic recurrence exhibited an accumulation of immunosuppressive myeloid cells expressing PLA2G2D in T-cell-rich cellular neighbourhoods of the paracortex. In mice, we pinpointed *Pla2g2d* expression to a specific population of macrophages, called LNMs, that are enriched in TDLNs and absent from tumours. Inhibiting PLA2G2D boosted tumour-specific CD8^+^ T cell responses and reduced tumour growth, indicating that immune checkpoints that are active mainly in TDLNs—outside the local TME—can control systemic anti-tumour immunity.

PLA2G2D^+^ myeloid cells were highly abundant in TDLNs from patients with stage III melanoma who later developed distant metastases, and inhibiting PLA2G2D suppressed the growth of LN-metastatic tumours in vivo. However, PLA2G2D induction and anti-PLA2G2D therapeutic efficacy were also observed in a non-metastatic context. These observations suggest that activation of the PLA2G2D checkpoint represents an early, LN-intrinsic event, which might facilitate tumour immune escape to allow metastatic or non-metastatic cancer recurrence. Nevertheless, the altered cellular contexture in the paracortex was specific to patients with stage III disease, indicating that major compositional and architectural changes in the TDLN occur later during the disease course—possibly also in response to tumour-cell seeding. Whereas IFNγ was sufficient to strongly induce the transcription of *PLA2G2D* in vitro, blocking IFNγ in vivo did not affect *Pla2g2d* expression levels in LNMs, suggesting that other signals are necessary or can compensate for the loss of IFNγ. Because *Pla2g2d* was expressed by LNMs in both TDLNs and non-draining LNs, and in both metastatic and non-metastatic models, direct tumour-cell engagement is unlikely to be required. Alternatively, circulating factors or cues from the LN microenvironment might be involved, including inflammatory mediators, oxidative stress or apoptotic cells^43,45^.

Our data firmly establish the TDLN as a nexus of PLA2G2D immune checkpoint activity. Macrophages in patients’ TDLN paracortex and T-cell-zone regions expressed the highest levels of PLA2G2D, which after secretion binds to an unidentified target^40^ on the surface of early activated T cells to limit their capacity to proliferate and produce cytokines. Although CD8^+^ T cells near PLA2G2D^+^ myeloid cells in TDLNs upregulated markers that are associated with an exhausted or progenitor-exhausted state, the PLA2G2D checkpoint seems to act mainly by suppressing the early activation and priming of T cells. At the time point at which inhibition of PLA2G2D induced a tumour-specific expansion of CD8^+^ T cells in our melanoma models and started to inhibit tumour growth in vivo (10–13 days after tumour inoculation), the expression of *Pla2g2d* was confined to LNMs, which closely resemble TZMs. Although our antibodies can inhibit TZM-derived PLA2G2D, tools to selectively deplete or delete PLA2G2D in TZMs are currently lacking^43^. Nevertheless, we also detected PLA2G2D^+^ DCs in patient TDLNs, and we could detect PLA2G2D protein in patient tumours, albeit at lower levels than in TDLNs. These observations suggest that PLA2G2D-mediated immune suppression extends beyond LNMs, and this effect is likely to depend on disease progression, tumour-intrinsic features and species differences. Age might be particularly relevant, and induction of PLA2G2D has been previously observed in CD11c^+^ DCs as mice age^45^. Others have implicated mediators downstream of PLA2G2D’s phospholipase activity in age-related immune suppression, in particular prostaglandins^39,45,46^. Notably, we found that PLA2G2D acts mostly independently of its enzymatic activity, and that *Pla2g2d* is strongly induced in the LNs of young tumour-bearing mice (8–10 weeks), indicating that this molecule has a broader and more complex role in vivo that will require further in-depth investigation.

Finally, we found that a combination therapy based on the co-inhibition of PLA2G2D and PD-1 was more effective than either therapy was alone, reflecting non-redundancy between checkpoint molecules. Especially in *PLA2G2D*-humanized mice bearing B16F10 melanoma tumours resistant to anti-PD-1 therapy, we observed a strong therapeutic synergy of anti-PLA2G2D and anti-PD-1 blocking antibodies. In other models that are more responsive to PD-1 blockade, we still detected additive effects of the combination therapy, as well as increased frequencies of tumour-specific and IL-2-producing CD8^+^ T cells within end-stage tumours. Although the mechanisms that underlie these additive or synergistic effects are unclear, the increased numbers of tumour-specific PD-1^+^ T cells—first in the TDLNs and subsequently detected in the TME—that we observed after PLA2G2D inhibition provide a larger pool of T cells amenable to PD-1 blockade, explaining the observed improved efficacy of combination therapy. Vice versa, whereas others have shown that *PLA2G2D* transcripts are upregulated early after anti-PD-1 treatment in patients independent of response^47^, *PLA2G2D* expression was associated with acquired resistance after initial tumour regression^16^. This suggests that chronic anti-PD-1-mediated T cell activation in tumours could evoke negative feedback through the PLA2G2D checkpoint, and that combination therapy has the potential to extend the duration of treatment responses. Testing these hypotheses will require additional studies in suitable mouse models of acquired ICI resistance and in clinical trials.

Altogether, our study identifies PLA2G2D as an early TDLN-centred immune checkpoint that restrains anti-tumour immunity. The myeloid TDLN–PLA2G2D axis can be targeted therapeutically using monoclonal antibodies, offering new strategies for stand-alone or ICI-based combination immunotherapy in cancer.

## Methods

### Ethics approval

This study was approved by the Erasmus MC Ethics Committee (MEC-2017-375, METC 2020-054), allowing for retrospectively collected data and residual material from tissue collected for routine diagnostic purposes to be used, waiving the need for explicit written informed consent. Human tissues and patient data were used according to the Code for Proper Secondary Use of Human Tissue and the Code of Conduct for the Use of Data in Health Research as stated by the Federation of Dutch Medical Scientific Societies. All animal studies were approved by the Institutional Animal Care and Use Committee at Mispro (South San Francisco) or the animal welfare committee (IvD) of the Erasmus MC (Rotterdam), with approval by the CCD (Centrale Commissie Dierproeven) under the permit number AVD101002017867.

### Patient inclusion

From patients with melanoma who underwent a sentinel LN procedure at the Erasmus MC Cancer Institute between 2005 and 2017, individuals with or without nodal metastasis (that is, positive TDLNs) were identified. For comparative purposes, we selected patients with melanoma who either presented with early (stage II, RFS ≤ 48 months; stage III, RFS ≤ 24 months) distant disease recurrence or did not develop disease recurrence (stage II, RFS > 96 months; stage III, RFS > 60 months) after the sentinel LN procedure. To avoid including false-negative TDLNs, we only included stage II patients with a negative TDLN who developed metastatic disease within the regional LNs (similar to the TDLN basin) after 9 months or longer. Patients with melanoma did not receive neoadjuvant or adjuvant treatment (which was not routinely administered during the inclusion period), excluding effects of immunotherapy on TDLN contexture or disease outcome. We also selected patients with resectable stage IB–III NSCLC who underwent surgical resection at the Erasmus MC Cancer Institute between 2018 and 2021, with either distant disease recurrence (>6 months and ≤60 months after surgery) or who did not develop disease recurrence (>60 months after surgery). Adjuvant chemotherapy for NSCLC was allowed but is known to improve overall survival only minimally after surgery^48^. Patients with melanoma or with NSCLC were excluded if they had synchronous malignancies of other origin, chronic infections, active autoimmune disease or other disease aetiologies requiring immunosuppressive treatment (including prednisone or equivalent >5 mg), or if they received neoadjuvant treatment before resection. Most patients with NSCLC in both prognostic groups received adjuvant chemotherapy according to local treatment protocols.

### Tissue sample processing and ROI selection

Formalin-fixed paraffin-embedded (FFPE) LN samples were retrieved from the Pathology department at the Erasmus MC (standard protocols for diagnostic work-up) and tissue sections were prepared at a thickness of 4 µm and 5 µm for IMC and IHC, respectively. ROIs (500 × 500 µm) were selected at four distinct LN anatomical locations: cortex, paracortex, peritumoural and intratumoural. ROI selection was guided by (i) IHC staining for SOX10 (tumour), CD3 (T cells) and CD20 (B cells), to prevent an exclusive focus on the highly prevalent B cell follicles; and (ii) haematoxylin and eosin (H&E), CD3, CD20 and SOX10-stained 5-µm sections from each sample. ROI selection was performed under the supervision of an experienced certified pathologist. Tissue microarrays (TMAs) were created using the fully automated TMA Grand Master (3DHISTECH) system. From the paracortex of TDLNs from patients with stage III melanoma, 2-mm-diameter cores were selected from the above-mentioned ROIs and embedded in paraffin.

### Design of the IMC antibody panel

IMC antibodies were either acquired pre-labelled with metal isotopes (Standard BioTools) or labelled in-house using MaxPar conjugation kits (Standard BioTools) according to the manufacturer’s protocol. Antibodies were titrated on TDLN material containing metastases and were evaluated by an experienced pathologist to determine optimal antibody concentrations. Details of antibodies and dilutions are provided in Supplementary Table 5.

### IMC tissue staining

For IMC, the antibody staining protocol was adapted from Standard BioTools. FFPE LN material was sliced at a thickness of 4 µm. Slides were baked for 2 h at 60 °C before being dewaxed in fresh xylene (Sigma-Aldrich, 534056) for 20 min. Slides were then dehydrated in descending grades of ethanol (ethanol 100%, Boom, 84901622.5000) (100%, 95%, 80%, 70%) for 5 min each and washed in sterile water for 5 min by gentle agitation. Slides were inserted in preheated antigen retrieval solution at 96 °C (Dako, S2367) and incubated for 30 min with loose lids. After incubation, slides were cooled down to 70 °C before being washed in sterile water for 10 min in 50-ml Falcon tubes by gentle agitation. Slides were then inserted in Epredia Shandon plastic cover plates (Epredia) and moved to the Sequenza Immunostaining Center System (Epredia). Slides were washed twice with phosphate-buffered saline (PBS) by filling the Sequenza cover plates and allowing the fluid to flow through the system. Next, the tissue was blocked using 3% bovine serum albumin (BSA) in PBS for 45 min at room temperature. Meanwhile, the antibody cocktail was prepared in 0.5% BSA in PBS. To each slide, 150 µl antibody cocktail was added, after which the Sequenza Immunostaining Center System was covered with a lid and incubated at 4 °C overnight. The next day, slides were washed with 0.2% Triton X-100 (Thermo Fisher Scientific, WD319895) in PBS until the fluid ran through the system. The slides were washed twice with PBS in the same manner before being stained with 150 µl Intercalator-Ir in PBS for 30 min at room temperature. Finally, the slides were washed with sterile water before being dried at room temperature. Slides were stored in a dry place until acquisition. Details of antibodies and dilutions are provide in Supplementary Table 5.

### Image acquisition

For IMC, images were acquired on the Hyperion Imaging System using CyTOF software v.7.1 (Standard BioTools). Laser strength was determined using representative tissue samples. The machine was tuned before every acquisition and reference energy was calibrated once a month. All intra- and peritumoural ROIs from patient P002 were excluded from further analysis owing to abnormal staining patterns.

### Cell segmentation

To generate single-cell data from IMC images, segmentation was performed in accordance with the Bodenmiller IMC segmentation pipeline^49^, including the use of CellProfiler v.4.2.5^50^ and Ilastik v.1.4.0^51^. In brief, MCD and .txt files were converted into individual tiff files using the IMC preprocessing script. Hot pixels were filtered by comparing the intensity of each pixel against the maximum intensity of the 3 × 3 neighbouring pixels; if differences were larger than a threshold of 50 (default settings), the pixel intensity was clipped to the maximum intensity in the 3 × 3 neighbourhood. Next, images were cropped in CellProfiler for use in supervised pixel classification using Ilastik, which was trained to recognize the nucleus, cytoplasm and background using selected markers (DNA, CD45, CD20, CD3, SOX10, and ICSK1–3 from the Cell segmentation kit III). We trained Ilastik on 50 × 50-µm cropped images of multiple ROIs from each patient. In brief, the average intensity across all channels is calculated and multiplied by 100 to help identify background regions. Next, the average expression was clipped to a range between 0 and 1. Ilastik stacks were upscaled by factor 2 and cropped to 50 × 50 pixels. After feature reduction, cell masks were generated, and images were segmented using CellProfiler to extract single-cell staining intensity data. This segmentation strategy was validated using Steinbock (v.0.16.3)—a toolkit for the processing of multiplex tissue images—which implements DeepCell’s Mesmer^52^ (v.0.4.1) for automated cell segmentation based on TissueNet, a dataset for training segmentation models with more than one million manually labelled cells. In brief, Steinbock was run using the Steinbock Docker container in Linux as provided by the authors. Raw .mcd files were preprocessed and filtered for hot pixels with a threshold of 50. Next, cells were segmented using Mesmer based on CD11c, CD14, CD45, CD68, CD20, CD8a, CD169 and CSK-III as cytoplasm markers and SOX10, DNAI and DNAII as nuclear markers. Single-cell data were extracted from segmented images by calculating the mean object intensities per channel, as well as spatial object properties (area, centroid, major axis length, minor axis length and eccentricity).

### Processing and normalization of IMC data

Data were processed using an R-based workflow for multiplex imaging data, as described previously^49^. In brief, data were transformed using an inverse hyperbolic sine function (asinh function from the rrscale v.1.0 package). The dataset then underwent median cell size calculation, with very small cells (diameter less than 10 pixels) excluded from further analysis. Shot noise was filtered mostly by performing all downstream analyses using median signal intensities per cell, with cells consisting, on average, of 80–90 pixels per µm^2^—minimizing the effect of rare random positive pixel artefacts. As a quantitative metric of image quality, we generated signal-to-noise ratio (SNR) values for all markers in our panel (Extended Data Fig. 1b). SNR values were calculated using a two-component Gaussian mixture model for each marker for each cell, calculated as SNR = *I*_s_/*I*_n_, in which *I*_s_ represents the signal intensity (mean intensity of pixels with a true signal) and *I*_n_ represents the noise intensity (mean intensity of pixels containing noise). Using the Otsu thresholding method, we identified pixels of foreground (signal) and background (noise), in which signal intensity is divided by noise intensity for each cell as described previously^49^. Most hallmark markers for cell-type identification (for example, CD4, CD8, CD20, CD68 and CD11c) showed relatively strong signal intensities and SNR values (>2). Transcription factors and specific cell-surface receptors showed lower staining intensities, yet all had positive SNR values (> 1.5). ROI coverage was calculated and any ROIs with a cell coverage lower than 50% were inspected to identify potential selection errors. To ensure data integrity, batch correction was then applied to the data per ablation run, as well as Ki-67 expression, using the fastMNN method^53^ (v.1.29).

### Cell-type annotation

FastMNN-corrected data were clustered using the Rphenograph package (v.0.1.1)^54^, with *k* = 40. The resulting clusters were then annotated using marker proteins to distinguish general lineages (B cells, CD4^+^ T cells, CD8^+^ T cells, T_reg_ cells, tumour, endothelial cells, myeloid cells, fibroblasts and stroma). These lineages were subclustered to remove cells that were wrongly annotated and to further stratify the lineages into more defined cell phenotypes. We refrained from using markers with relatively low signal intensity and SNR values (Extended Data Fig. 1b) for phenotyping (for example, BCL6 and PD-L1). To increase annotation accuracy, we limited definitions of key cell types to naive and non-naive T cells (based on CD3, CD4, CD8A, FOXP3, CD45RA, Ki-67, CTLA4, PD-1 and HLA-DR); DCs (CD11c^hi^CD68^lo^) separated on the basis of high or low HLA-DR levels; and macrophages (CD11c^lo^CD68^hi^) separated on the basis of high levels of CD163 or CD169. The phenotyping of cells was validated by careful inspection of labels projected on cell masks in combination with marker expression on images, often resulting in several rounds of visual inspection and subsequent parameter optimization (for example, marker-based gating of individual cell phenotypes using cytomapper).

### Cell–cell interaction and neighbourhood analysis

Interaction graphs were calculated using ‘buildSpatialGraph’ from the imcRtools package v.0.1.8 (ref. ^49^), with the Delaunay triangulation method, resulting in a definition of the nearest neighbours for each cell in the IMC dataset (maximum distance parameter = 20). Per ROI image, we used ‘testInteractions’ to compute the average cell-type–cell-type interaction counts and compared this count against an empirical null distribution generated by permuting all cell labels, while maintaining the tissue structure. This way, every pairwise interaction was tested for statistically significant interaction (+1) or avoidance (−1) in every ROI. Sums of interact and avoid scores were visualized in a heat map as percentages of ROIs with a particular label. ‘testInteractions’ was also used to compute the average interaction count for each cell phenotype pair across the entire dataset, including label permutation to enable statistical testing. Next, we inferred cellular NBs using ‘aggregateNeighbors’ based on the Delauney interaction graph, in which cells were counted by their phenotypic label and separated by disease stage as well as by location. Data were then *k*-means-clustered (*k* = 10), from which the resulting NBs were visualized using a heat map for each patient group (stage II or III; R or DF) and location (for example, paracortex, intratumoural) separately. Cell distances were calculated using ‘minDistToCells’ from the imcRtools package (v.0.1.8)^49^. Next, dyads were defined as surrounding cells within 15 μm of target cells as measured from the centroid of cells. We then calculated cell distances to cells labelled as DC–CD8 dyads and quantified macrophage abundance near these dyads (within 15 μm or 30 μm), separated by patient group.

### Image visualization

Composite images of selected channels were generated using Fiji ImageJ (v.1.54t)^55^. Images were processed to remove outlier pixels, filtered using a median or Gaussian filter (0.5-pixel radius) and scaled to enhance contrast as described previously^56^, or visualized using the plotPixels function in the cytomapper^57^ package.

### GeoMx digital spatial profiling

Digital spatial profiling (DSP) using the GeoMx Whole Transcriptome Atlas (WTA) (NanoString Technologies) was performed following the manufacturer’s instructions. In brief, 5-µm-thick slices were cut from TMAs and stained for SYTO 13 (NanoString Technologies, 1:20), CD11c–Alexa Fluor 647 (Abcam, EP1347Y, 1:40), CD8a–Alexa Fluor 594 (BioLegend, C8/144B, 1:40) and CD68–Alexa Fluor 532 (Novus Biologicals, C68/684, 1:40), and incubated overnight with GeoMx WTA probes. Within the TDLN paracortex, regions in which CD11c^+^, CD68^+^ and CD8^+^ cells co-localized were selected. From these regions, WTA probes from CD11c^+^, CD8^+^ and CD68^+^ cells were collected separately and sequenced using an Illumina NextSeq2000. Paired-end clusters were generated, producing reads that were 26 bases in length. We chose to remove segments with less than 15% of all genes detected. The resulting counts were normalized using Q3 normalization and DEGs (*P* < 0.05) were calculated using a linear mixed model (LMM) as implemented in the GeomxTools v.3.10.0 R package (https://doi.org/10.18129/B9.bioc.GeomxTools). Pathway enrichment analyses on the DEGs were done using Metascape^58^.

### GSEA

We ran GSEA as implemented by the clusterProfiler^59^ (v.4.3.3) R package against custom gene signatures for DCs^21,60–62^, macrophages^22^ and CD8^+^ T cells^33,34^. The GSEA function was implemented with default settings, using a Benjamini–Hochberg correction for multiple testing of the resulting *P* values.

### Analyses of publicly available transcriptome data

TCGA transcriptome data for skin cutaneous melanoma (SKCM) were obtained from the UCSC Xena portal (https://tcga.xenahubs.net). To calculate the levels of *PLA2GD2* in various types of melanoma sample, we used log_2_(*x* + 1)-transformed RNA-Seq by Expectation Maximization (RSEM)-normalized counts and curated phenotype data as directly available from the UCSC Xena portal. To investigate which cytokines in the TME correlate with *PLA2G2D* expression, Pearson’s *R* coefficients and *P* values were determined for a range of cytokines and *PLA2G2D* across various tumour types included in TCGA. In the case of type I (α and β subtypes were included) and type III (λ) interferons, and in the case of TGF-β isoforms, the gene with the highest *R* coefficient per cytokine was plotted. Analyses were performed using the GEPIA2 TCGA data browser^63^. Log_2_-transformed fold changes in the RNA expression of genes in NSCLC biopsies before anti-PD-1 therapy (pre) and after (post) onset of acquired therapy resistance were obtained from a previous study^16^.

### IHC and multiplex immunofluorescence

PLA2G2D was stained by automated IHC using the Ventana Benchmark ULTRA v.12.1 (Ventana Medical Systems). FFPE sections of 4-µm thickness were stained for PLA2G2D (Supplementary Table 6) using OptiView (760-700, Ventana). In brief, after deparaffinization and heat-induced antigen retrieval with CC1 (950-500, Ventana) for 32 min, the tissue samples were incubated with PLA2G2D antibody for 32 min at 37 °C. Incubation was followed by Optiview detection and haematoxylin II counterstaining for 8 min followed by a blue colouring reagent for 8 min according to the manufacturer’s instructions (Ventana, Roche). Multiplex immunofluorescence analysis was performed by automated immunofluorescence using the Ventana Benchmark Discovery ULTRA (Ventana Medical Systems). Slides of 4-µm-thick FFPE sections were stained for CD11c, CD8, CD68, LAMP3 and CXCL13 (Supplementary Table 6). In brief, after deparaffinization and heat-induced antigen retrieval with CC1 (950-224, Ventana) for 32 min, incubation with anti-CD8 was performed for 32 min at 37 °C, followed by Omnimap anti-mouse HRP (760-4310, Ventana) and detection with R6G (950-240, Ventana). An antibody denaturation step was performed with CC2 (950-123, Ventana) at 100 °C for 20 min. Second, incubation with anti-CD68 was performed for 12 min at 37 °C, followed by Omnimap anti-mouse (760-3210 Ventana) and detection with DCC (760-244, Ventana). An antibody denaturation step was performed with CC2 (950-123, Ventana) at 100 °C for 20 min. Third, incubation with anti-LAMP3 was performed for 60 min at 37 °C, followed by Omnimap anti-rabbit (760-3211 Ventana) and detection with Red610 (760-245, Ventana) for 8 min. An antibody denaturation step was performed with CC2 at 100 °C for 20 min. Fourth, incubation with anti-CD11c was performed for 12 min at 37 °C, followed by Omnimap anti-rabbit HRP (760-4311, Ventana) and detection with Cy5 (760-238, Ventana) for 8 min. An antibody denaturation step was performed with CC2 at 100 °C for 20 min. Finally, anti-CXCL13 was incubated for 20 min at 37 °C, followed by Omnimap anti-rabbit HRP (760-4311, Ventana) and detection with FAM (760-243, Ventana) for 8 min. Finally, slides were washed in PBS and mounted with Vectashield containing 4′,6-diamidino-2-phenylindole (Vector Laboratories). Slides were imaged with an Axioscan 7 (ZEISS).

### Automated quantification of PLA2G2D^+^ cells

PLA2G2D-stained whole slides were scanned using the NanoZoomer (20×, Hamamatsu, HT2.0) and analysed using the open-source digital pathology software QuPath (v.0.5.1)^64^. All slides were set as ‘Brightfield (H-DAB)’, and for each slide, stain vectors were set for haematoxylin and DAB. ROIs of 200 µm by 200 µm in TDLNs and primary tumour tissues were selected for positive cell detection. Interfollicular regions and the centre of the primary tumour or the LN metastasis were selected for ROIs, and germinal centres, tissue borders and anthracotic areas were excluded from ROI selection. The number of ROIs per tissue depended on the tissue size. Cell segmentation and positive cell detection was performed on the basis of nuclear detection and mean DAB stain intensity using the built-in QuPath positive cell detection command, with the following settings: detection image, haematoxylin OD; requested pixel size, 0.5 µm; background radius, 8.0 µm, performing background by reconstruction; median radius, 0.0 µm; sigma, 1.5 µm; minimum area, 10.0 µm, maximum area, 200.0 µm; threshold, 0.1; maximum background, 2.0; cell expansion, 4.0 µm, including nuclei and smooth boundaries. Three staining intensity thresholds were set using the following settings: threshold compartment: cytoplasm: DAB OD mean, threshold positive 1+, 0.08; threshold positive 2+, 0.16; threshold positive 3+, 0.24. For each slide, ROI measurements were exported and used for analysis. Per TDLN or tumour, as well as per patient, the average of all ROIs per tissue was used to calculate the percentage of positive cells, positive cells per mm^2^ and the number of different positive cell intensities (1+, 2+ or 3+).

### Collection of TDLN tissue from patients with melanoma or NSCLC for cell culture

We collected fresh TDLN tissue from patients with NSCLC or melanoma who underwent surgery at the Erasmus MC. Viable cells were obtained using the scrape cytology method, as described previously^65,66^. In brief, excised LNs were bisected during the standard pathology workflow, and a scalpel blade was used to shave along the cut surface and rinsed in 3 ml RPMI culture medium to obtain cells. This way, the LN samples could still be used for further processing without interfering with diagnostic workflows. The cell suspensions were filtered through a 35-μm filter cap (Falcon) and centrifuged at 400*g* for 7 min. The medium was discarded and the cells were cryopreserved at −196 °C in RPMI containing 40% fetal bovine serum (FBS) and 10% dimethyl sulfoxide. All TDLN tissue samples were stored after resection at 4 °C and processed within 18 h.

### Flow-cytometry analysis

TDLN samples were thawed in RPMI + 10% FBS, centrifuged for 7 min at 400*g*, resuspended in RPMI with 10% FBS and incubated for 60 min at 37 °C. Then, cells were washed and resuspended in FACS buffer (0.5% BSA, 10% NaN_3_ sodium azide and 2 mM EDTA in PBS) and stained for cell-surface markers (Supplementary Table 7) in Brilliant Stain Buffer (BD) for 30 min at 4 °C. Cells were washed with PBS and labelled with Fixable Viability Stain 575V (BD) for 15 min at 4 °C. For intracellular staining, the FoxP3 fixation/Permeabilization Kit (eBioscience) guidelines were followed. All cells were then washed and resuspended in FACS buffer before analysis on a BD FACSymphony A5 flow cytometer (FACS Diva software v.9; BD).

### TDLN T cell in vitro stimulation assay

TDLN samples were thawed and stained for cell-surface molecules as described above. Cells were washed and resuspended in MACS buffer (0.5% BSA and 2 mM EDTA in PBS). Before cell sorting, Helix NP Blue was added according to the manufacturer’s guidelines, and sorting was performed using a BD FACSAria III. We sorted three CD8^+^ T cell populations: CD8^+^ T naive (alive lineage^*−*^CD5^+^CD8^+^CCR7^+^CD45^+^), CD8^+^ T non-naive PD-1^int^ (alive lineage^*−*^CD5^+^CD8^+^non(CCR7^+^CD45RA^+^)PD-1^int^) and CD8^+^ T non-naive PD-1^hi^ (alive lineage^*−*^CD5^+^CD8^+^non(CCR7^+^CD45RA^+^)PD-1^hi^). The lineage cocktail contained antibodies against CD19, CD20, CD11c, CD68, CD56, CD14, CD16 and CD123. CD5 was used instead of CD3 to allow stimulation with anti-CD3 after sorting. In the TDLN samples, the lineage-negative CD5^+^ cell population overlapped with the lineage-negative CD3^+^ cell population by more than 98%, as verified by flow cytometry. Sorted cells were collected in 100% FBS. Then, cells were resuspended in culture medium consisting of RPMI with 10% FBS, 1% penicillin–streptomycin and primocin (100 µg ml^−1^). Per patient, concentrations were determined on the basis of the condition with the lowest cell yield, so that the other conditions contained cells *x* times the lowest cell count. The antibodies and culture materials used are listed in Supplementary Table 7. To mimic chronic T cell activation, stimulation was performed using plates coated with anti-CD3 at 5 µg ml^−1^ 24 h before culture. Cells were seeded in 100 µl culture medium with the addition of 2 µg ml^−1^ soluble anti-CD28 at day 0. Cells were centrifuged, resuspended in culture medium and passaged onto a fresh anti-CD3-coated plate on days 2, 3 and 4, and analysed on day 5. This assay was adapted from a previous study^67^. On day 5, cells were washed and stained for cell-surface and intracellular molecules as described above. Cells were analysed using a BD FACSymphony A5. FlowJo v.10.0 was used for data analysis.

### Production of recombinant PLA2G2D protein

Recombinant human or mouse PLA2G2D proteins were generated by fusing the cDNA of human (NM_012400.4) or mouse (NM_011109.3) PLA2G2D with the Fc region of human IgG1 on the C terminus in the pcDNA3.4 expression vector (Thermo Fisher Scientific). An enzymatic-dead mutant of PLA2G2D–Fc was generated by introducing an H47Q mutation into the human PLA2G2D cDNA. An effector-less Fc-tag variant was generated by introducing L234A, L235A and P329G mutations into the human IgG1 fusion tag. PLA2G2D–Fc proteins were expressed by transiently transfecting Expi293 cells using the Expi293 Expression System Kit (Thermo Fisher Scientific) according to the manufacturer’s instructions. After 5 days of culture, supernatants were collected and recombinant proteins were purified by protein A affinity chromatography using a HiTrap PrismA column (Cytiva) on a Bio-Rad NGC FPLC system.

### In vivo tumour models

C57BL/6 and BALB/c mice were purchased from the Jackson Laboratory or Envigo, housed in specific pathogen-free conditions and fed ad libitum. *Pla2g2d*^−/−^ mice were generated by CRISPR–Cas9-mediated gene editing in which deletion of exon 2 of the *Pla2g2d* gene results in loss of function through a frameshift from exon 1 to exon 3. Proximal (5′-CGAAAACCTAAGAGCCTGCG-3′) and distal (5′-TAGGTGGCTGGAACAACCAT-3′) *Pla2g2d* guide RNAs and Cas9 protein were injected into C57BL/6NTac zygotes using established methods. *PLA2G2D*-humanized mice on a C57BL/6 background were generated by Ozgene using gene targeting by homologous recombination. In brief, the mouse *Pla2g2d* genomic locus from residues L21 within exon 2 to the stop codon in exon 4 was replaced with human cDNA encoding residues I22 through to the stop codon. Gene targeting was performed on C57BL/6 ES cells. LN6-987AL and B16F0 cells^19^ were provided by N. Reticker-Flynn and cultured in Dulbecco’s modified Eagle’s medium (DMEM). MC38 and B16F10 (ATCC) tumour-cell lines were cultured in DMEM, and E.G7-OVA and CT26 (ATCC) tumour-cell lines were cultured in RPMI medium. All media were supplemented with Glutamax, gentamycin (50 µg ml^−1^) and 10% FBS. Cell lines were not subjected to further authentication but were routinely tested for mycoplasma contamination. To prepare mice for tumour inoculation, they were shaved on the right flank. MC38, B16F10, CT26 or E.G7-OVA (10^6^ cells) and LN6-987AL or B16F0 (2 × 10^5^ cells) were collected and washed twice with PBS before subcutaneous inoculation (in 100 µl PBS) into mice. Mice were monitored daily, and tumours were measured by calliper every 3–4 days. When tumours reached 50–150 mm^3^ and were palpable, mice were randomized into groups and antibodies or proteins were administered i.p. every 3–4 days for a total of four doses. Mice with palpable tumours were injected with anti-PLA2G2D, anti-IFNγ (BioXCell) or isotype control antibodies (all 10 mg kg^−1^) in 200 µl PBS. Anti-mouse PD-1 antibody (clone RMP1-14, Leinco) was dosed at 5 mg kg^−1^ using the same schedule. Recombinant PLA2G2D–Fc protein was administered i.p. at 50 µg in 100 µl PBS per dose every 4 days for a total of four doses. For CD8^+^ T cell depletion experiments, anti-CD8 depleting antibodies (200 µg) were administered i.p. one day before the initiation of anti-PLA2G2D treatment. Antibody administration (mouse IgG2a isotype control; anti-mouse CD8a, clone YTS169, BioXCell) was repeated every 3–4 days and continued throughout the duration of the experiment. In some experiments, blood, LNs, TDLNs and tumours were collected at the indicated time points after tumour inoculation. Single-cell suspensions of LNs and TDLNs were prepared by mechanically dispersing the LNs through a 100-μm nylon-mesh cell strainer. For tumours, single cells were prepared using the Tumour Dissociation Kit (Miltenyi). After staining with antibodies as described above, cell suspensions were analysed by flow cytometry. Mice were killed when tumours greater than 2,000 mm^3^ developed or when tumour ulceration was observed. Mice (only females) were 8–12 weeks old when used in experiments. Sample sizes were determined on the basis of previous experience with the tumour models (for example, variability in growth kinetics in vivo), and on the basis of the practical availability of specific mice. Blinding was not applied in this study. Source data for all in vivo experiments are provided.

### Single-cell RNA-seq and data analysis

C57BL/6 mice were inoculated with B16F0-tdTomato or LN6-987AL-tdTomato cells as described above and treated with isotype or anti-PLA2G2D antibodies at days 8 and 11. At day 12, tumours, TDLNs and non-draining LNs were collected and single-cell suspensions were prepared and stained for extracellular antibodies as described above. Using a FACSAria III (BD), cells were pooled from four sorted LN (or TDLN) populations that were mixed in equal ratios: (1) alive cells; (2) CD45^*−*^ cells; (3) CD45^+^CD3^+^CD44^+^ cells; and (4) combined CD45^+^CD3^*−*^CD11b^+^ + CD45^+^CD3^*−*^CD11c^+^ cells. From processed tumours, cells were pooled and mixed from three sorted fractions: (1) alive cells; (2) CD45^+^CD3^+^CD44^+^ cells; and (3) CD45^+^CD3^*−*^CD11b^+^ + CD45^+^CD3^*−*^CD11c^+^ cells. Next, pooled cell fractions were encapsulated for cDNA synthesis and barcoded with multiplexed runs on a Chromium X using the GEM-X 3′ OCM Chip (on-chip multiplexing). Final sequencing libraries were generated following the instructions of the GEM-X Universal 3′ Gene Expression v4 4-plex user guide (CG000768). Sequencing was performed on a Novaseq 6000 platform (Illumina) using an S2 (100 cycle) flow cell with a 28-10-10-90 cycle setting. The target sequencing depth of each library was >25,000 reads per cell. Sequencing reads were aligned to the mouse mm39 (GRCm39-2024-A) reference genome and quantified using CellRanger (10X Genomics, v.9.01). CellRanger output was loaded into R using Seurat (v.5.3.0) and filtered (200–6,000 features per cell, less than 15% mitochondrial DNA). Next, counts were log-normalized, variable features were called (nfeatures = 3000) and the data were scaled. Next, a principal component analysis was run and a sample batch correction was performed using Harmony (v.1.2.3). Clusters were identified on the basis of shared-nearest-neighbour clustering using the first 30 principal components (PCs), with a resolution of 0.3 and *k* = 30. The first 30 PCs were then used to generate UMAP projections with a minimum distance of 0.3. Clusters were annotated using known gene markers: T cells (*Cd3e*,* Cd3g*,* Cd3d*,* Cd4*,* Cd8a* and* Cd8b*), B cells (*Ms4a1* and* Cd19*), DCs (*Cst3* and* Flt3*), macrophages (*Cd68* and* Csf1r*), neutrophils (*S100a9*), NK cells (*Klrb1c*) and tumour (*Stmn1*). In addition, FindMarkers with a Wilcoxon rank-sum test and an absolute log_2_-transformed fold change cut-off of 1 was used to identify cluster marker genes and further stratify cell annotation. Previously published gene signatures of DCs and macrophage populations^68,69^ were used to validate the identity of myeloid subsets. Published breast cancer (GSE167036) and lung adenocarcinoma (GSE131907) datasets containing both tumour and LN (or TDLN)-derived cells were processed, clustered and annotated as described in the original papers, with some simplification of complex annotations to improve comparisons between datasets. PLA2G2D expression was projected onto a UMAP of cells from the LNs (or TDLNs) using Seurat.

### Bone-marrow chimera experiments

*Pla2g2d*^+/+^ (CD45.1^+^) and *Pla2g2d*^−/−^ (CD45.2^+^) mice (8–12 weeks old) were lethally irradiated with two doses of 500 cGy administered 4–6 h apart (total 1,000 cGy) to induce myeloablation. After irradiation, mice received antibiotics in their drinking water for 2–3 weeks. Bone marrow was collected from the tibiae and femora of congenic *Pla2g2d*^+/+^ C57BL/6 (CD45.1^+^) and *Pla2g2d*^−/−^ (CD45.2^+^) donor mice (6–8 weeks old). Cells were flushed with RPMI supplemented with 10% FBS, filtered through a 70-μm strainer and subjected to ACK lysis to remove erythrocytes. Cells were washed, counted and resuspended in PBS at 1 × 10^6^ cells per 100 μl. Recipient mice received bone-marrow cells by retro-orbital injection. Donor chimerism was assessed 6 weeks later by flow cytometry. After chimerism was confirmed, mice were subcutaneously implanted with MC38 tumour cells for downstream analysis of tumour growth.

### Generation of anti-PLA2G2D antibodies

Antibodies against PLA2G2D were generated by immunizing BALB/c mice with recombinant human PLA2G2D–Fc-tagged proteins. Mice were immunized at multiple sites using complete Freund’s adjuvant for multiple boosts. Test bleeds were performed by saphenous vein lancing seven days after the last boost. When the antibody titre was high enough, mice were given a final intravenous boost in the lateral tail vein, after which immunized mice were killed and spleens isolated. Hybridomas were generated by electrofusion of splenocytes with Sp2/0 myeloma cells. Fused cells were plated into 96-well plates in HAT selective medium and grown for 10–14 days to generate hybridoma clones, which were assayed for binding to human PLA2G2D proteins by ELISA. Positive binders were expanded and antibodies in the supernatants were purified by protein G (HiTrap Protein G HP, GE Healthcare). The specificity of anti-PLA2G2D antibodies was determined by verifying their lack of binding to other related sPLA2 family members by ELISA. In brief, recombinant PLA2G1B, PLA2G2A, PLA2G2E, PLA2G2F, PLA2GV and PLA2G2X Fc-tagged proteins were generated as described above for PLA2G2D–Fc. Binding to sPLA2 family members was tested by ELISA. Purified proteins were coated at 0.5 µg ml^−1^ on 96-well plates and titrations of anti-PLA2G2D antibodies or isotype control antibodies were applied, followed by HRP-conjugated secondary detection and visualization with TMB-Ultra reagent (Thermo Fisher Scientific). The specificity of the antibodies was further tested by evaluating their ability to inhibit the enzymatic activity of PLA2G2D–Fc or the various sPLA2 family members in a phospholipase enzyme assay as previously described^37^.

### Isolation and culture of human peripheral blood cells

Human white blood cell concentrate was obtained from Stanford Blood Center or the Sanquin Blood Bank, and peripheral blood mononuclear cells (PBMCs) were prepared by Ficoll density gradient. Pan T cells, CD8^+^ T cells and monocytes were isolated from human PBMCs using Pan T, CD8^+^ T Cell and Pan Monocyte isolation kits (Miltenyi) according to the manufacturer’s instructions, respectively.

### Stimulation of T cells in PBMC-based assays

PBMCs were labelled with CellTrace CFSE (Invitrogen) and T cells were stimulated with 1 µg ml^−1^ anti-CD3 and 0.2 µg ml^−1^ anti-CD28 antibodies in the presence of recombinant PLA2G2D–Fc, PLA2G2D(H47Q)–Fc or Fc control in RPMI. After 48 h of incubation, cell-culture supernatants and cells were collected for cytokine production and flow-cytometry analysis, respectively. IL-2 and IFN-γ levels were measured using Meso Scale Discovery Human or Mouse V-Plex/U-Plex kits. T cell proliferation was measured by multicolour flow cytometry on a BD LSRFortessa X-20 flow cytometer using CD3, CD4, CD8 and live–dead markers for live T cell gating, followed by CFSE dilution analysis for proliferation analysis according to the manufacturer’s instructions. The binding of PLA2G2D to T cells was determined by incubating various concentrations of PLA2G2D–Fc or Fc control proteins to resting or activated T cells for 30 min, followed by washing and secondary detection with fluorophore-labelled anti-human IgG antibodies and subsequent analysis on a flow cytometer.

### Stimulation of isolated T cells for surface–PLA2G2D binding kinetics

CD8^+^ cells were isolated and cultured as described above and stimulated with T cell TransAct (Miltenyi). At the indicated time points, cells were collected, washed and stained with PLA2G2D–His for 30 min at 4 °C in PBS. Next, the cells were stained with anti-His-APC together with other extracellular antibodies and live–dead markers as described above. All cells were then washed and resuspended in FACS buffer before analysis on a BD FACSymphony A5 flow cytometer.

### Stimulation of isolated T cells for intracellular cytokine staining and proliferation assays

CD8^+^ cells were isolated and cultured as described above and stimulated with T cell TransAct (Miltenyi) in the presence or absence of 10 µg ml^−1^ PLA2G2D protein, which was added again after 48 h. For proliferation assays, isolated CD8^+^ cells were labelled with the CellTrace Violet Cell Proliferation Kit (0.05 µM, Thermo Fisher Scientific) before starting the culture. After 72 h of incubation, cells were collected, washed and stained with antibodies and live–dead markers as described above. For intracellular cytokine staining, cells were then fixed with 2% paraformaldehyde for 20 min at room temperature and permeabilized with 0.5% saponin for 10 min at 4 °C, after which the cells were stained for intracellular cytokines in 0.5% saponin for 60 min at 4 °C. All cells were then washed and resuspended in FACS buffer before analysis on a BD FACSymphony A5 flow cytometer.

### Bulk RNA-seq of stimulated T cells

CD8^+^ cells were isolated and cultured as described above and stimulated with T cell TransAct (Miltenyi) in the presence or absence of 10 µg ml^−1^ PLA2G2D protein. After 24 h of incubation, cells were collected and washed, and RNA was isolated using the RNeasy Micro kit (QIAGEN) according to the manufacturer’s instructions. Samples for bulk RNA-seq were sequenced and analysed as described before^70^. In brief, raw counts and reads per kilobase million (RPKM) were determined on the exons of RefSeq-annotated genes using HOMER’s (v.5.1)^71^ analyzeRepeats.pl command. The R package DESeq2 v.1.48.2 was used for the identification of DEGs, sample scaling and statistical analysis. Genes with an RPKM < 1 in 50% of replicates in one condition were excluded. DEGs were determined by an absolute log_2_-transformed fold change value > 0.5 and an adjusted *P* value < 0.05. Pathway enrichment analysis on DEGs was performed using Metascape^58^ (v.3.5).

### Mixed lymphocyte reactions

For mouse allogeneic mixed lymphocyte reactions, human monocyte-derived DCs were differentiated from monocytes using the DC differentiation toolbox (Miltenyi) according to the manufacturer’s instructions. Monocyte-derived DCs were matured in the presence of TNF where indicated. DCs were then treated with mitomycin-C for 1 h at 37 °C, followed by three washes with RPMI, then added to a 96-well round-bottomed plate. T cells were added to DCs at a DC-to-T cell ratio of 1:10 in RPMI, and were incubated for 5 days before analysis of cytokine levels by MSD or collection of conditioned medium. Where indicated, 0.5 µg ml^−1^ recombinant PLA2G2D–Fc, PLA2G2D(H47Q)–Fc, Fc control or anti-PD-1 (pembrolizumab; 5 mg ml^−1^ or 10 mg ml^−1^) were added during the allogeneic coculture.

### Quantitative PCR

Total RNA was isolated from in vitro differentiated macrophages, MDMs and DCs using the RNeasy Mini Kit (QIAGEN) according to the manufacturer’s instructions. Total RNA was synthesized into cDNA using the High-Capacity cDNA Reverse Transcription Kits (Applied Biosystems). Quantitative PCR with reverse transcription (qRT–PCR) was performed using Power SYBR Green PCR Master Mix (Applied Biosystems) per the manufacturer’s instructions. The amplification condition was set at 40 cycles at 95 °C for 15 s and 60 °C for 60 s using an ABI Prism 7900HT Sequence Detection System (Applied Biosystems). β-actin (*ACTB*) and hypoxanthine phosphoribosyl transferase (*HPRT1*) were used for normalization. Cq values for the two reference genes were determined by ABI SDS software (v.1.5.1) and used to calculate the geometric mean of *ACTB* and *HPRT1* as the normalization factor using GeNorm. The normalization factor was applied to *PLA2G2D* data, and values were converted into conventional fold changes by setting the value of a control sample to 1 as indicated, scaling all other values proportionally. Primer sequences used:

*PLA2G2D* forward: 5′-CAACCCAAAGATGCCACG-3′ and reverse: 5′-CCAGCTTCCCTTGTCAGAG-3′.

*ACTB* forward: 5′-ATTGCCGACAGGATGCAGAA-3′ and reverse: 5′-GCTGATCCACATCTGCTGGAA-3′. *HPRT1* forward: 5′-GACCAGTCAACAGGGGACAT-3′ and reverse: 5′-AACACTTCGTGGGGTCCTTTTC-3′.

### Statistical analysis

For IMC data analyses, differential cell abundances were calculated using a two-sided *t*-test (Rstatix v.0.7.2; https://rpkgs.datanovia.com/rstatix/). Wilcoxon signed-rank tests and unpaired *t*-tests were implemented for non-normally and normally distributed data, respectively, using the stat_compare_means() function included in ggpubr v.0.6.0. Correlations were calculated as Pearson’s correlation coefficient (*r*) as implemented by ggpubr v0.6.0 (https://rpkgs.datanovia.com/ggpubr/) using the stat_cor function. For DSP whole-transcriptome analysis, DEGs were calculated using a LMM as implemented in the GeomxTools v.3.10.0 R package. Statistical analyses of the flow cytometry, tumour growth curves, survival analysis and IHC and immunofluorescence results were performed in GraphPad Prism v.10. Error bars reported for bar graphs in the figures denote s.e.m. unless indicated otherwise. For box plots, boxes indicate the median and interquartile range, with whiskers representing the data distribution excluding outliers. Paired or unpaired *t*-tests were used for comparisons of paired data with Gaussian distributions, and paired or unpaired Wilcoxon tests were performed for non-normally distributed paired data. *P* values (and adjusted *P* values) smaller than 0.05 were considered statistically significant; exact *P* values are provided in the Supplementary Data. Data points used in the figures were obtained from independent biological samples. When representative data are shown, similar results were obtained from one or more independent replicate experiments.

### Reporting summary

Further information on research design is available in the Nature Portfolio Reporting Summary linked to this article.

## Online content

Any methods, additional references, Nature Portfolio reporting summaries, source data, extended data, supplementary information, acknowledgements, peer review information; details of author contributions and competing interests; and statements of data and code availability are available at https://doi.org/10.1038/s41586-026-10954-1.

## Supplementary information

Supplementary Tables 1 and 3–7 Supplementary Table 1. Characteristics of patients with melanoma selected for IMC. Supplementary Table 3. Characteristics of patients with melanoma selected as a validation cohort. Supplementary Table 4. Characteristics of patients with non-small cell lung cancer. Supplementary Table 5. IMC antibody panel. Supplementary Table 6. Antibodies used for IHC and multiplex immunofluorescence. Supplementary Table 7. Antibodies used for flow-cytometry analysis and cell-culture reagents.

Reporting Summary

Supplementary Table 2 Differentially expressed genes from GeoMx DSP analysis

Supplementary Data Additional information on statistics, including exact *P* values

Peer Review File

## Source data

Source Data Figs. 3–5 and Source Data Extended Data Figs. 9 and 10

## Data Availability

The PLA2G2D blocking antibodies developed for this study are made available to the scientific community and can be requested from L.-F.L. and K.V.L. Raw flow-cytometry, IHC and immunofluorescence data files generated and analysed for the current study are available upon reasonable request. IMC, GeoMx and single-cell transcriptomic data have been deposited in publicly accessible databases (the Gene Expression Omnibus or Zenodo): IMC data: https://doi.org/10.5281/zenodo.20038486 (ref. ^72^); GeoMx data: GSE282437; bulk RNA-seq data: https://doi.org/10.5281/zenodo.20314670 (ref. ^73^); and single-cell RNA-seq data: GSE333464. Source data are provided with this paper.

## Code availability

Computer code used for data analysis and figure plotting is available through GitHub: https://github.com/pulmed/Spatial-proteogenomics-of-tumour-draining-lymph-nodes-.git.
