# Supplementary material for: PLA2G2D in tumour-draining lymph nodes regulates anti-tumour immunity
Source: Nature. 2026 Sep 9;658(8135):485–95. doi: 10.1038/s41586-026-10954-1 (PMC13645638; doi:10.1038/s41586-026-10954-1)
Supplement: Supplementary file 1 [file 41586_2026_10954_MOESM1_ESM.pdf]

nature portfolio

https://doi.org/10.1038/s41586-026-10954-1

Supplementary information

PLA2G2D in tumour-draining lymph nodes regulates anti-tumour immunity

In the format provided by the authors and unedited

PLA2G2D in tumour-draining lymph nodes regulates anti-tumour immunity

Anneloes van Krimpen[1,2,*], Julie Huang[3*], Mike Eterman[1,2#], Vivian Gerretsen[1,2,12#], Michihisa Umetani[3,4#], Josephine C. Janssen[2,7#], Menno van Nimwegen[1], Nina Rozendaal[1], Thierry P.P. van den Bosch[8], Xinguo Jiang[3], Kathryn Logronio[3], Angela Z. Liu[3], Yun-Ru Liu[5], Yuki Nagasaki[6], Makoto Murakami[6], Anne Onrust-Van Schoonhoven[1], Asabi Leliveld[1], Disha Vadgama[1], Hedwig Langeveld[1], Rogier van Wijck[8], Eric Bindels[9], Jan von der Thüsen[8], Antien Mooyaart[8], Febe van Maldegem[10], Claudia M. Brenis[11,12], Stijn Verwaerde[11,12], Rudi W. Hendriks[1], Bart N. Lambrecht[1,11,12], Dirk J. Grünhagen[2,7], Cornelis Verhoef[2,7], Joachim G.J.V. Aerts[1,2], Li-Fen Lee[3§], Kan V. Lu[3§], Ralph Stadhouders[1,2§] & Floris Dammeijer[1,2§]

[1]Department of Pulmonary Medicine, Erasmus MC University Medical Centre, Rotterdam, the Netherlands
[2] Erasmus MC Cancer Institute, Erasmus MC University Medical Centre, Rotterdam, the Netherlands
[3]Apeximmune Therapeutics, Burlingame, CA, USA
[4]Department of Pharmacological & Pharmaceutical Sciences, University of Houston, Houston, TX, USA
[5]Taipei Medical University, Taipei City, Taiwan
[6]Center for Disease Biology and Integrative Medicine, Graduate School of Medicine, The University of Tokyo, Tokyo, Japan
[7]Department of Surgical Oncology, Erasmus MC University Medical Centre, Rotterdam, the Netherlands
[8]Department of Pathology, Erasmus MC University Medical Centre, Rotterdam, the Netherlands.
[9]Department of Haematology, Erasmus MC University Medical Centre, Rotterdam, the Netherlands.
[10]Department of Molecular Cell Biology and Immunology, Amsterdam UMC, Amsterdam, the Netherlands.
[11]Department of Internal Medicine and Pediatrics, Ghent University, Ghent, Belgium
[12]Laboratory of Immunoregulation and Mucosal Immunology, VIB-UGent Centre for Inflammation Research, Ghent, Belgium

**Equal contribution (first author)*
[#]*Equal contribution (second author)*
[§]*Equal contribution (senior & corresponding author)*

SUPPLEMENTARY DATA

Supplementary Tables 1 & 3-7 (Supplementary Table 2: separate .xlsx file)

Supplementary Table 1 – Melanoma IMC patient characteristics

Supplementary Table 1 - Characteristics of melanoma patients selected for Imaging Mass Cytometry. P-values for categorical variables were calculated using a Chi-squared test. For continuous variables, P-values were calculated using an independent sample T test.

		**ALL PATIENTS N=21**	**STAGE 2**		**STAGE 3**		**P VALUE**
			**Recurrence >96 months**	**Recurrence <48 months**	**Recurrence > 60 months**	**Recurrence < 24 months**	
PATIENT CHARACTERISTICS							
**AGE (MEAN, 95%CI)**		52.9 (48.2-57.6)	54 (44.9-63.1)	52.6 (40.0-65.3)	50.5 (37.5-63.6)	54.8 (38.8-70.8)	0.920
**SEX**							0.613
	Female	9	2	1	3	3	
	Male	12	3	4	3	2	
TUMOR CHARACTERISTICS							
**BRESLOW (MM) MEAN (95%CI)**		3.3 (2.5-4.1)	3.8 (0.6-7.0)	3.6 (1.4-5.9)	2.8 (1.1-4.4)	3.2 (1.9-4.5)	0.791
**ULCERATION**							
	Absent	10	5	1	3	1	0.036
	Present	11	0	4	3	4	
**HISTOLOGY**							
	SSM	12	3	3	2	4	
	NM	6	0	2	3	1	
	Unknown	3	2	0	1	0	
**LOCATION**							0.637
	Arm	5	2	2	0	1	
	Leg	4	0	1	2	1	
	Trunk	10	2	2	4	2	
	Head & neck	2	1	0	0	1	
SLN SURGICAL REMOVAL							
**SN REGION**							0.682
	Axillary	13	3	4	3	3	
	Iguinal-iliac	6	1	1	3	1	
	cervical	2	1	0	0	1	
OUTCOME							
**TIME TO RECURRENCE (MONTHS)**				14.6 ± 11.3		11.8 ± 5.3	

Supplementary Table 3 - Melanoma validation cohort

Supplementary Table 3 - Characteristics of melanoma patients selected as a validation cohort. P-values for categorical variables were calculated using a Chi-squared test. For continuous variables, P-values were calculated using an independent sample T test.

		NO RECURRENCE > 60 MONTHS N=10	RECURRENCE < 24 MONTHS N=14	P-VALUE
PATIENT CHARACTERISTICS				
**AGE (MEAN, 95%CI)**		57.5 (51.2-63.8)	55.9 (50.9-60.8)	0.652
**SEX**				0.408
	Female	6	5	
	Male	4	9	
PRIMARY TUMOR CHARACTERISTICS				
**BRESLOW (MM) MEAN (95%CI)**		3.9 (2.7-5.1)	5.2 (3.4-7.0)	0.195
**ULCERATION**				0.134
	Absent	6	3	
	Present	4	10	
	unknown	0	1	
**HISTOLOGY**				0.257
	SSM	6	3	
	NM	3	5	
	SSM/NM	0	1	
	ALM	1	2	
	Unknown	0	3	
**LOCATION**				0.804
	Upper limb	2	2	
	Lower limb	5	6	
	Trunk	3	6	
OUTCOME				
**TIME TO RECURRENCE (MONTHS) MEAN ± SD**			11.8 ± 6.3	
**RELAPSE SITE**				
	Lymphnodes		2	
	Lung		5	
	Liver		2	
	Bone		1	
	intransit		3	
	Multiple organs		1	

Supplementary Table 4 – NSCLC patient characteristics

Supplementary Table 4 - Characteristics of non-small cell lung cancer patients. P-values for categorical variables were calculated using a Chi-squared test.For continuous variables, P-values was calculated using an independent sample T test.

		NO RECURRENCE > 60 MONTHS N= 9	RECURRENCE < 60 MONTHS N= 9	P-VALUE
PATIENT CHARACTERISTICS				
**AGE (MEAN, SD)**		62.2 years (6.0)	69.3 years (12.8)	0.152
**SEX**				0.165
	Female	7	4	
	Male	2	5	
PRIMARY TUMOR CHARACTERISTICS				
**T-STAGE**				0.228
	T1	2	1	
	T2	4	3	
	T3	2	3	
	T4	1	2	
**N-STAGE**				1.000
	N1	6	6	
	N2	3	3	
**HISTOLOGY**				0.332
	Squamous cell carcinoma	4	2	
	Adenocarcinoma	4	6	
	NSCLC NOS	1	1	
**TDLN LOCATION**				0.999
	N1 station (hilar, 9,10,11,12)	8	8	
	N2 station (4)	-	1	
**PD-L1**				0.089
	NEG	3	3	
	POS	2	5	
**LOCATION**				0.564
	Right upper lobe	5	5	
	Middle lobe	-	1	
	Right lower lobe	1	2	
	Left upper lobe	1	-	
	Left lower lobe	2	1	
OUTCOME				
**TIME TO RECURRENCE (MONTHS) MEAN ± SD**			23 ± 14	
**RELAPSE SITE**				
	Lymph nodes		2	
	Lung		2	
	Pleura		1	
	Bone		1	
	Cerebral		2	
	Multiple organs		1	

Supplementary Table 5 – IMC Antibodies

Supplementary Table 5 - Imaging Mass Cytometry panel.

Antibody	Dilution	Label	Company	Clone	#Cat
**BCL6**	300	172Yb	CST	E5I8I	56263
**CD11c**	200	142Nd	CST	D3V1E	#45581
**CD127**	400	168Er	SBT	EPR2955(2)	3168026D
**CD14**	1000	144Nd	SBT	EPR3653	3144025D
**CD141**	200	141Pr	CST	E7Y9P	#43514
**CD15**	100	149Sm	CST	MC480	74180
**CD163**	500	147Sm	SBT	EDHu1	3147021D
**CD169**	200	163Dy	Abcam	SP213	ab245735
**CD1c**	1000	148Nd	Abcam	EPR23189-196	ab270797
**CD20**	400	161Dy	SBT	H1	3161029D
**CD25**	100	175Lu	Abcam	SP176	ab238272
**CD27**	100	171Yb	Abcam	EPR8569	ab256583
**CD3**	200	170Er	Abcam	SP162	ab245731
**CD31**	800	151Eu	CST	89C2	85873
**CD4**	500	156Gd	SBT	EPR6855	3156033D
**CD45**	1000	152Sm	SBT	D9M8I	3152018D
**CD45RA**	400	166Er	SBT	HI100	3166031D
**CD68**	400	159Tb	SBT	KP1	3159035D
**CD69**	1000	164Dy	Abcam	EPR21814	ab234512
**CD8a**	400	162Dy	CST	D8A8Y	81575
**Collagen**	600	169Tm	SBT	Polyclonal	3169023D
**CSK-I**	1000	195Pt	SBT	-	TIS-00001
**CSK-II**	1000	196Pt	SBT	-	TIS-00001
**CSK-III**	1000	198Pt	SBT	-	TIS-00001
**CTLA-4**	400	160Gd	Abcam	CAL49	ab251599
**D2-40**	200	153Eu	CST	LpMab-12	#26981
**DNAI**	800	191Ir	SBT	-	201192A
**DNAII**	800	193Ir	SBT	-	201192A
**FoxP3**	1600	155Gd	Abcam	221D	ab255942
**GATA3**	1600	176Yb	CST	D13C9	#5852
**Granzyme B**	400	167Er	SBT	EPR20129-217	3167021D
**HLA-DR**	1000	174Yb	SBT	LN3	3174025D
**IDO**	100	145Nd	CST	D5J4E	#86630
**Ki-67**	200	146Nd	CST	8D5	#9449
**PD-1**	200	165Ho	CST	EH33	60847
**PD-L1**	300	150Nd	CST	E1L3N	85164
**SOX10**	200	158Gd	CST	E6B6I	69661
**TCF1**	200	173Yb	CST	C63D9	#2203
**TIM-3**	800	154Sm	CST	D5D5R	81229
**TOX**	400	143Nd	CST	E6I3Q	#73758

Supplementary Table 6 – Histology antibodies

Supplementary Table 6 - Antibodies used for IHC and multiplex immunofluorescence

ANTIBODY	DILUTION	SPECIES	COMPANY	CLONE	AB INCUBATION **time** at 37˚C
**CD11C**	2.15 ug/ml	rabbit	Cell Marque	SP157	12 minutes
**CD8**	1.04 ug/ml	mouse	DAKO	C8/144B	32 minutes
**CD68**	3.49 ug/ml	mouse	Ventana	KP1	12 minutes
**LAMP3**	1/100	rabbit	Atlas Antibodies	Polyclonal	60 minutes
**CXCL13**	1/1200	rabbit	Invitrogen	polyclonal	20 minutes
**PLA2G2D**	1/800	rabbit	Invitrogen	polyclonal	32 minutes

Supplementary Table 7 – Flowcytometry & culture reagents

Supplementary Table 7 – Flowcytometry Human panel A

PRODUCT	FLUOCHROME	CLONE	SUPPLIER	CATALOGUE #	CONCENTRATION
**CD19**	FITC	HIB-19	BD	555412	1:20
**CD20**	FITC	2H7	Invitrogen / eBioscience	11-0209-42	1:40
**CD11C**	FITC	B-ly6	BD	564490	1:20
**CD68**	FITC	Y1/82A	Biolegend	333806	1:10
**CD56**	FITC	HCD56	Biolegend	318304	1:10
**CD14**	FITC	61D3	Invitrogen / eBioscience	11-0149-42	1:10
**CD16**	FITC	3G8	BD	555406	1:10
**CD123**	FITC	6H6	Biolegend	306014	1:10
**HELIX NP BLUE**	BV421		Biolegend	425305	

Supplementary Table 7 - Flowcytometry Human panel B

PRODUCT	FLUOCHROME	CLONE	SUPPLIER	CATALOGUE #	CONCENTRATION
**CD4**	APC-H7	RPA-T4	BD	560158	1:20
**CD223 (LAG-3)**	AF700	11C3C65	Biolegend	369344	1:20
**CD197 (CCR7)**	BV510	G043H7	Biolegend	353232	1:20
**CD366 (TIM-3)**	BV750	F38-2E2	Biolegend	345055	1:20
**CD152 (CTLA-4)**	BV421	BNI3	BD	562743	1:20
**CD39**	BV711	TU66	BD	563680	1:20
**CD279 (PD-1)**	PE	MIH4	Invitrogen / eBioscience	12-9969-42	1:10
**CD8A**	PerCp-Cy5.5	RPA-T8	Invitrogen / eBioscience	45-0088-42	1:20
**CD45RA**	PE-TxR	MEM-56	Invitrogen / eBioscience	MHCD45RA17	1:20
**CD5**	PE-Cy7	UCHT2	Biolegend	300622	1:30
**FIXABLE VIABILITY STAIN 575V**	BV575		BD	565694	1:500
**TOX**	APC	REA473	Miltenyi Biotec	130-118-335	1:20
**KI67**	BV605	Ki67	Biolegend	350522	1:100
**TIGIT**	BV711	741182	BD	747839	1:20
**CD69**	FITC	FN50	BD	555530	1:20
**CD279 (PD-1)**	BV786	EH12.2H7	Biolegend	329939	1:20

Supplementary Table 7 – Flowcytometry Human panel C

PRODUCT	FLUOCHROME	CLONE	SUPPLIER	CATALOGUE #	CONCENTRATION
**CD4**	APC-Cy7	RPA-T4	BD	560158	1:25
**CD3**	AF700	UCHT1	eBioscience	56-0038-42	1:25
**CD8**	PE-TXR	RPA-T8	BD	562282	1:50
**IL-2**	BV650	Mq1-17H12	BD	564166	1:25
**IFNY**	BV711	B27	BD	564039	1:25

Supplementary Table 7 – Flowcytometry Mouse antibodies

PRODUCT	FLUOCHROME	CLONE	SUPPLIER	CATALOGUE #	CONCENTRATION
**CD62L**	PE-Dazzle	MEL-14	Biolegend	104448	1:200
**CD8**	PE	53-6.7	Invitrogen / eBioscience	12-0081-83	1:800
**CD44**	BV650	IM7	BD	740455	1:400
**CD4**	APC-H7	GK1.5	BD	560181	1:50
**CD45**	BV786	30-F11	BD	564225	1:1600
**CD3**	Biotin	145-2C11	BD	553060	1:100
**FIXABLE VIABILITY DYE**	eFluor506		Invitrogen / eBioscience	65-0866-14	1:200
**CD45**	PerCP-Cy5.5	1600	30-F11	eBioscience	45-0451-82
**CD11B**	PE-Cy7	1000	M1/70	BD	552850
**CD44**	APC-Cy7	800	IMF7	BD	560568
**CD3**	FITC	100	145-2C11	BD	553062
**CD11C**	APC	200	N418	Invitrogen / eBioscience	17-0114-81
**CD44**	BV650	IM7	Biolegend	103049	1:400
**PD1**	BV421	J43	BD	562584	1:100
**CD8**	BV605	53-6.7	Biolegend	100744	1:800
**CD4**	APC-H7	GK1.5	BD	560181	1:50
**CD45**	BV786	30-F11	BD	564225	1:1600
**CD3**	Biotin	145-2C11	BD	553060	1:100
**FOXP3**	AF700	FJK-165	Invitrogen / eBioscience	56-5773-82	1:100
**KI67**	FITC	SolA15	Invitrogen / eBioscience	11-5698-82	1:400
**CD8**	APC	53-6.7	Invitrogen / eBioscience	17-0081-82	1:400
**CD3**	PE-Cy7	145-2C11	Biolegend	100320	1:100
**CD44**	PerCP-Cy5.5	IM7	Invitrogen / eBioscience	45-0441-82	1:800
**CD45**	AF700	30-F11	Biolegend	103128	1:100
**CD8**	BV786	53-6.7	Biolegend	100750	1:400
**IL-2**	APC	JES6-5H4	BD	554429	1:100
**H-2KB TRP-2 TETRAMER-SVYDFFVWL**	PE		Dr. Thorbald van Hall (LUMC)		1:200

Supplementary Table 7 – Flow cytometry materials

PRODUCT	CLONE	SUPPLIER	CATALOGUE #	CONCENTRATION
Human TruStain FcX		Biolegend	422302	
Brilliant Stain Buffer		BD	563794	
Permeabilization Buffer (10X)		Invitrogen / eBioscience	00-8333-56	
Fixation/Permeabilization Concentrate		Invitrogen / eBioscience	00-5123-43	

Fixation/Permeabilization Diluent	Invitrogen / eBioscience	00-5223-56
Saponin from Quillaja Bark	Merck	S7900-100G
Paraformaldehyde	Merck	16005

Supplementary Table 7 – Cell culture materials

**PRODUCT**	**CLONE**	**SUPPLIER**	**CATALOGUE #**	**CONCENTRATION**
RPMI 1640 Medium, GlutaMAX Supplement		ThermoFisher	61870010	
Fetal Bovine Serum		BODINCO BV	BDC-S00O12	1:10
Penicillin-Streptomycin (10,000 U/mL)		ThermoFisher	15140122	1:100
Primocin		InvivoGen	ant-pm-1	100 µg/mL
Purified anti-human CD3	OKT-3	Biolegend	317302	0,5 µg/mL
Purified anti-human CD28	CD28.2	Biolegend	302902	0,2 µg/mL
DMEM glutamax		Gibco	10566016	
Gentamicin		Gibco	15710-049	50 µg/mL
<u>FACS and MACS buffer</u>				
Dulbecco's Phosphate Buffered Saline		Gibco	14190-094	
BSA		Sigma-Aldrich	A-8327	
EDTA		Sigma-Aldrich	03690	
