# Supplementary material for: PLA2G2D in tumour-draining lymph nodes regulates anti-tumour immunity
Source: Nature. 2026 Sep 9;658(8135):485–95. doi: 10.1038/s41586-026-10954-1 (PMC13645638; doi:10.1038/s41586-026-10954-1)
Supplement: Supplementary file 2 [file 41586_2026_10954_MOESM2_ESM.pdf]

Corresponding author(s): Ralph Stadhouders PhD, Floris Dammeijer MD PhD, Kan V. Lu PhD, Li-Fen Lee PhD

Last updated by author(s): Jul 6, 2026

Reporting Summary

Nature Portfolio wishes to improve the reproducibility of the work that we publish. This form provides structure for consistency and transparency in reporting. For further information on Nature Portfolio policies, see our Editorial Policies and the Editorial Policy Checklist.

Statistics

For all statistical analyses, confirm that the following items are present in the figure legend, table legend, main text, or Methods section.

n/a	Confirmed	
☐	☒	The exact sample size (*n*) for each experimental group/condition, given as a discrete number and unit of measurement
☐	☒	A statement on whether measurements were taken from distinct samples or whether the same sample was measured repeatedly
☐	☒	The statistical test(s) used AND whether they are one- or two-sided<br>*Only common tests should be described solely by name; describe more complex techniques in the Methods section.*
☒	☐	A description of all covariates tested
☐	☒	A description of any assumptions or corrections, such as tests of normality and adjustment for multiple comparisons
☐	☒	A full description of the statistical parameters including central tendency (e.g. means) or other basic estimates (e.g. regression coefficient) AND variation (e.g. standard deviation) or associated estimates of uncertainty (e.g. confidence intervals)
☐	☒	For null hypothesis testing, the test statistic (e.g. *F*, *t*, *r*) with confidence intervals, effect sizes, degrees of freedom and *P* value noted<br>*Give P values as exact values whenever suitable.*
☒	☐	For Bayesian analysis, information on the choice of priors and Markov chain Monte Carlo settings
☒	☐	For hierarchical and complex designs, identification of the appropriate level for tests and full reporting of outcomes
☐	☒	Estimates of effect sizes (e.g. Cohen's *d*, Pearson's *r*), indicating how they were calculated

Our web collection on statistics for biologists contains articles on many of the points above.

Software and code

Policy information about availability of computer code

Data collection	MCD™ Viewer v1.1.0 (Standard BioTools), CyTOF Software v7.1 (Standard BioTools), Ventana Benchmark Discovery ULTRA v12.1 (Ventana Medical Systems Inc.), FACS Diva software v9 (BD Biosciences), ABI SDS software v1.5.1 (ABI)
Data analysis	R v4.4.2, CellProfiler v4.2.5, Ilastik v1.4.0, Rphenograph v0.1.1, rrscale v.1.0, imcRtools v0.1.8, Fiji ImageJ v1.54t, Steinbock v0.16.4, Mesmer 0.4.1, GeomxTools v3.10.0, clusterProfiler v4.3.3, QuPath v0.5.1, Rstatix v0.7.2, ggpubr v0.6.0, GraphPad Prism v10, Cytomapper v3.2.0, Flowjo v10.0, Cellranger v9.01, Harmony v1.2.3, Seurat v5.3.0, fastMNN v1.29, HOMER v5.1, DESeq2 v1.48.2, Metascape 3.5. Details on application of this software is described in the Methods section.<br><br>Computer code used for data analysis and figure plotting has been made available through GitHub: https://github.com/pulmed/Spatial-proteogenomics-of-tumour-draining-lymph-nodes-.git.

For manuscripts utilizing custom algorithms or software that are central to the research but not yet described in published literature, software must be made available to editors and reviewers. We strongly encourage code deposition in a community repository (e.g. GitHub). See the Nature Portfolio guidelines for submitting code & software for further information.

Data

Policy information about availability of data

All manuscripts must include a data availability statement. This statement should provide the following information, where applicable:

- Accession codes, unique identifiers, or web links for publicly available datasets
- A description of any restrictions on data availability
- For clinical datasets or third party data, please ensure that the statement adheres to our policy

IMC data and GeoMx/single-cell transcriptome data have been deposited into publicly accessible databases. IMC data, doi:10.5281/zenodo.20038486; GeoMx data, GSE282437; bulk RNA-Seq data, doi:10.5281/zenodo.20314670; scRNA-Seq, GSE333464. Published breast cancer (GSE167036) and lung adenocarcinoma (GSE131907) datasets containing both tumour and (tumour-draining) lymph node-derived cells were obtained from GEO.

Research involving human participants, their data, or biological material

Policy information about studies with human participants or human data. See also policy information about sex, gender (identity/presentation), and sexual orientation and race, ethnicity and racism.

Reporting on sex and gender	Findings apply to both male and female. Sex was not considered in study design. Data on sex differences among patient samples have been reported in Supplementary Tables.
Reporting on race, ethnicity, or other socially relevant groupings	Race and ethnicity were not reporting on nor where they taken into consideration during study design.
Population characteristics	These are described in Supplementary Tables 1, 3 & 4.
Recruitment	Patient sample selection is described in the Methods section.
Ethics oversight	The Erasmus MC Ethics Committee.

Note that full information on the approval of the study protocol must also be provided in the manuscript.

Field-specific reporting

Please select the one below that is the best fit for your research. If you are not sure, read the appropriate sections before making your selection.

☒ Life sciences ☐ Behavioural & social sciences ☐ Ecological, evolutionary & environmental sciences

For a reference copy of the document with all sections, see nature.com/documents/nr-reporting-summary-flat.pdf

Life sciences study design

All studies must disclose on these points even when the disclosure is negative.

Sample size	Sample size was not determined prior to the study. IMC and DSP sample size was determined by the number of available matched patients matching our inclusion criteria, resulting in similarly-sized groups.<br>Sample size for flow cytometry was determined on the available TDLN samples with sufficient cell yield after processing.<br>Sample size for IHC staining was determined on available FFPE samples from the selected patients with either DF or R disease status.<br>For other experiments, sample sizes were chosen based on prior experience (i.e. sufficient to obtain robust results) or based on successful examples from literature.
Data exclusions	For IMC data analysis, intra- and peritumoral regions from P002 were excluded due to abnormal nuclear staining limiting accurate cell segmentation.<br>For flow cytometry analysis, samples were excluded from analysis if not enough cells were left for proper subgroup analysis.<br>Concerning the histology samples, samples were excluded if no tissue was left in the FFPE block, or if the tissue did not meet quality standards for image analysis.
Replication	For all experiments in the paper, we at least performed two independent experiments. Attempts at replication were all successful. A validation cohort of melanoma or NSCLC TDLNs was selected to confirm our findings in the DSP and IMC TDLN data.
Randomization	Patient samples were chosen based on the disease recurrence status. Since this allocation was not random, sample selection in both groups was tested for differences in clinical characteristics other than disease-free or recurrence. No significant differences were found. For animal expriments, we randomized animals into groups prior to treatment.
Blinding	Blinding was not applied in this study. Given the objective nature of the outcome measures and the limited opportunity for observer bias, the added complexity and logistical burden of blinding were not considered to provide a meaningful reduction in bias.

Reporting for specific materials, systems and methods

We require information from authors about some types of materials, experimental systems and methods used in many studies. Here, indicate whether each material, system or method listed is relevant to your study. If you are not sure if a list item applies to your research, read the appropriate section before selecting a response.

Materials & experimental systems

n/a	Involved in the study
☐	☒ Antibodies
☐	☒ Eukaryotic cell lines
☒	☐ Palaeontology and archaeology
☐	☒ Animals and other organisms
☒	☐ Clinical data
☒	☐ Dual use research of concern
☒	☐ Plants

Methods

n/a	Involved in the study
☒	☐ ChIP-seq
☐	☒ Flow cytometry
☒	☐ MRI-based neuroimaging

Antibodies

Antibodies used	All antibodies are described in Supplementary Tables 5-7 (including dilutions).
Validation	All antibodies were primarily validated by the manufacturers using multiple assays, including Western Blot, IHC/IF and Flow cytometry. Detailed information for individual antibodies used can be found online at the manufacturer's websites. All antibodies were used according to manufacturer’s guidelines, and tested and optimized on tissues or cells.

Eukaryotic cell lines

Policy information about cell lines and Sex and Gender in Research

Cell line source(s)	Murine B16F10, MC38, E.G7-OVA and CT26 tumor cells were obtained from ATCC. LN6-987AL and B16F0 cells were kindly provided by dr. Reticker-Flynn (Stanford University, USA). Expi293 cells were purchased from ThermoFisher.
Authentication	Cell lines were not authenticated after purchase.
Mycoplasma contamination	All cell lines tested negative for mycoplasma.
Commonly misidentified lines (See ICLAC register)	no commonly misidentified cell lines were used.

Animals and other research organisms

Policy information about studies involving animals; ARRIVE guidelines recommended for reporting animal research, and Sex and Gender in Research

Laboratory animals	C57BL/6 and BALB/c mice were purchased from Jackson Laboratory. All mice were housed (21 degrees Celsius, 55% humidity) and bred under SPF conditions and analyzed at 8-12 weeks of age. Lighting conditions mimic natural conditions (i.e. biodynamic light); 6.30h sunrise / 18.30h sunset.
Wild animals	no wild animals were used.
Reporting on sex	Only female mice were used.
Field-collected samples	no field collected samples were used.
Ethics oversight	All animal studies were approved by the Institutional Animal Care and Use Committee at Mispro (South San Francisco) or the animal welfare committee (IvD) of the Erasmus MC (Rotterdam), with approval by the CCD under the permit number AVD101002017867.

Note that full information on the approval of the study protocol must also be provided in the manuscript.

Plants

Seed stocks

Report on the source of all seed stocks or other plant material used. If applicable, state the seed stock centre and catalogue number. If plant specimens were collected from the field, describe the collection location, date and sampling procedures.

Novel plant genotypes

Describe the methods by which all novel plant genotypes were produced. This includes those generated by transgenic approaches, gene editing, chemical/radiation-based mutagenesis and hybridization. For transgenic lines, describe the transformation method, the number of independent lines analyzed and the generation upon which experiments were performed. For gene-edited lines, describe the editor used, the endogenous sequence targeted for editing, the targeting guide RNA sequence (if applicable) and how the editor was applied.

Authentication

Describe any authentication procedures for each seed stock used or novel genotype generated. Describe any experiments used to assess the effect of a mutation and, where applicable, how potential secondary effects (e.g. second site T-DNA insertions, mosiacism, off-target gene editing) were examined.

Flow Cytometry

Plots

Confirm that:

☒ The axis labels state the marker and fluorochrome used (e.g. CD4-FITC).

☒ The axis scales are clearly visible. Include numbers along axes only for bottom left plot of group (a 'group' is an analysis of identical markers).

☒ All plots are contour plots with outliers or pseudocolor plots.

☒ A numerical value for number of cells or percentage (with statistics) is provided.

Methodology

Sample preparation

Sample preparation and tissue processing have been described in the methods.

Instrument

BD Symphony A5 and BD Aria III

Software

Flowjo v10.0 was used for data analysis

Cell population abundance

We sorted 100% CD8+ T cell populations, of which the purity was >80% after 5 days of sorting. Due to the heterogeneic character of the samples, the three populations sorted varied in abundance among the patient samples.

Gating strategy

Baseline characterization of melanoma TDLN samples: FSC/SSC; single cells (FSC-A/FSC-H) ; alive cells (Live/Dead negative) ; SSC-A/CD3-positive population ; CD4-neg CD8-positive population ; all single markers.
Sort gating strategy: FSC/SSC ; single cells (FSC-H/FSC-W) ; single cells (SSC-A/SSC-W) ; alive cells (NP Helix negative) ; lineage-neg/CD5-positive population ; CD5-pos/CD8-pos population ; Tnaive (CCR7+ CD45RA+) and Tnon-naïve PD1-intermediate and Tnon-naïve PD-1hi population (as illustrated in figure S8B).
Day 5 exhaustion assay gating strategy: FSC/SSC ; single cells (FSC-A/FSC-H) ; alive cells (Live/Dead negative) ; SSC-A/CD5-positive population ; CD4-neg CD8-positive population ; all single markers.
Mouse single-cell RNA-sequencing gating strategy: cells were pooled from 4 sorted (TD)LN populations that were mixed in equal ratios: i) alive ii) CD45- iii) CD45+CD3+CD44+ iv) combined CD45+CD3-CD11b+ and CD45+CD3-CD11c+ cells. From processed tumours, cells were pooled and mixed from 3 sorted fractions: i) alive ii) CD45+CD3+CD44+ iii) CD45+CD3-CD11b+ or CD45+CD3-CD11c+ cells.

☒ Tick this box to confirm that a figure exemplifying the gating strategy is provided in the Supplementary Information.
