# Supplementary material for: PLA2G2D in tumour-draining lymph nodes regulates anti-tumour immunity
Source: Nature. 2026 Sep 9;658(8135):485–95. doi: 10.1038/s41586-026-10954-1 (PMC13645638; doi:10.1038/s41586-026-10954-1)
Supplement: Supplementary file 3 [file 41586_2026_10954_MOESM5_ESM.pdf]

nature portfolio

Peer Review File

PLA2G2D in tumour-draining lymph nodes regulates anti-tumour immunity

Corresponding Author: Dr Ralph Stadhouders

Any redactions in this file are there to maintain patient confidentiality, the confidentiality of unpublished data, or to remove third-party material.

This file contains all reviewer reports in order by version, followed by all author rebuttals in order by version.

Version 1:

Reviewer comments:

Referee #1

(Remarks to the Author)
Authors from this article studied Tumor Draining Lymph Nodes from stage II (non metatstatic) and III (metastatic) melanoma patients using spatial analysis. They concluded that patients who undergo recurrence within 2 years display specific cellular interactions in the paracortex region of the DLN and involving Tregs, DC, macrophages and CD8 T cells. They state that these CD8 T cells are poised for exhaustion and that this state is associated with chronic IFN signaling. Furthermore, they established that DC and macrophages from these areas express high levels of PLA2G2D. Study of tumor growth in PLA2G2D full KO mice or by systemic blocking using an Ab, showed a negative effect on tumor growth. Altogether, there are some interesting data in this study, especially for the effect of the PLA2G2D blocking on B16 melanoma, but also some overstatements, a lack of mechanistic studies to explain the effect of PLA2G2D blocking and the role of the cell clusters observed in TDLN from recurrent patients. This lack of mechanisms weakens the link between the 2 stories from this study with the spatial analysis on one side and the part focusing on PLA2G2D on the other.

Major concerns:
Although the initial study covered the analysis of stage 2 and 3 melanoma patients, it appears that only stage 3 patients display differences between their DC/Mac ratio between DF and R patients. After figure 2e, all the study focuses only on stage 3/metastatic patients but not stage 2 patients. This should be emphasized in the abstract as it restricts the potential interest of this discovery.
Furthermore, this suggests that the presence of tumor cells in the TDLN is required to induce the phenotype observed, i.e. the presence of complexes of cells composed of macrophages, DCs, Tregs and CD8 T cells. Even though the authors studied the paracortical area away from the tumor, tumor cells are still residing in the same LN than the T cells studied, and it is quite likely that these T cells are in the end quite similar to the ones found in the primary tumor or in the vicinity of the primary tumor. A direct comparison with primary tumor samples would be of major importance to prove that this cluster of cells and their state is specific to the TDLNs from recurrent patients and not just a common feature to cells found in the tumor.
The author states that the CXCL13+ CD8 T cells found in these clusters are poised for T cell exhaustion. This is not proven in the paper. First, the authors used tumor free TDLN for the study of PD1+ or naïve CD8 T cells, although they showed that the immunosuppressive myeloid cells that discriminate DF from R are found in stage 3 but are not discriminant in stage 2 patients. The term poised is related to an epigenetic imprinting of the cells, which is not studied here. Furthermore, their conclusions are drawn from 5 days "chronic" stimulation from naïve or PD1+ sorted CD8 T cells. The initial protocol cited by the author used a stimulation other 10 days (changing CD3 coated plates every other day). A 5 days stimulation, even when changing plates every day, is not that different than a classical stimulation. In that case, it is surprising to see that barely any naive stimulated T cell express PD-1 or TIM-3, that are also activation markers. Are the cells activated? Any activation markers are expressed? The slight increase of some markers is not sufficient to conclude to a poised exhausted state.
Effect of the KO of pla2g2d on tumor development was already described in (Miki et al JBC 2016) and pan cancer TCGA analysis also pointed out to the potential use of pla2g2d as marker for anti-PD1 resistance (Yang et al, Nat Comm 2021). These 2 articles should be cited in the article.
In figure 4, the authors validated that PLA2G2D protein is more expressed in TDLN from recurrent patients compared to disease free patients from stage 3 patients. The authors also mentioned that intratumor PLA2G2D was not significantly

different between DF and R patients. One unanswered question is whether the blocking of PLA2G2D is important in the TDLN or in the tumor.
Figure 4 C: A co-staining with CD11b/CD11c would help to pinpoint which cell types expresses PLA2G2D.
Figure 4 D-F: In mouse, the authors showed that the KO or the blocking of PLA2G2D favor tumor growth but do not describe further any effect on the immune system. Many questions should be answered concerning the mechanisms in place. Is blocking important within the tumor itself or in the TDLN? Are macrophages (or DC) really the source of PLA2G2D? A targeted KO should be done on both subtypes to demonstrate this point. Are the cellular interactions found in patients' TDLN also found in mice? Are they disrupted when PLA2G2D is blocked? Are tumor specific CD8 T cells more active/numerous within the tumor upon treatment? What is the general effect on immune cells? Answering these questions would help to render the study less descriptive and more impactful.
Minor concerns:
After figure 2g, the authors focused on the neighborhood cluster 2. However, in supplementary figure 3, neighborhoods 1, 6 and 8 are also significantly different. Could the author also discuss these clusters briefly?

Referee #2

(Remarks to the Author)
The authors present a technical tour-de-force profiling the organisation of tumour draining lymph nodes from patients with stage II or stage III melanoma who had either progressed or shown regression free survival following treatment. This led to them identifying changes in organisation within the paracortex which correlated with patient outcome. Interestingly this seemed to relate to the presence of interactions between CD8 T cells and specific myeloid subsets. They then identify PLA2G2D as being expressed highly by the myeloid cells present in these neighbourhoods and investigate this showing that high numbers of PLA2G2D+ cells correlate with recurrence. Finally they show that inhibition of PLA2G2D could lead to reduced tumour growth and sensitisation to PD1 blockade in the B16F10 model of melanoma. Given the relatively recent increased appreciation for the importance of the tumour draining lymph node in driving immunotherapeutic responses in vivo this is an important further development and highlights the potential that lies within studying the organisation of the node in defining therapeutic directions. While PLA2G2D has been previously implicated in control of immune responses in other settings, including cancer, this was the first time looking in a tumour draining lymph node and, given the findings, is highly significant. The paper is written clearly and concisely, with appropriate references to previous work and the conclusions appear robust. There are some concerns, however, needing addressing:
Issues:
1. Image preprocessing: The authors state "To ensure data integrity, batch correction was then applied to the data per ablation-run, as well as Ki-67 expression, using the fastMNN method". However, one can still observe some batch effects on the UMAP shown in Supplementary Figure 2.d (e.g. patients 19 & 21). On the other hand, raw images suffer from several sources of technical noise that affect the counts of each channel (protein, DNA), which mask the biological signal and hinder following analysis steps, such as hot pixels and shot noise. Did the authors consider such sources of noise? It will make the analysis more robust if the authors present some quantitative metrics to assess image quality, such the SNR (signal to noise ratio).
2. Cell segmentation: The authors performed the segmentation using Ilastik and CellProfiler, "in accordance with the Bodenmiller IMC segmentation pipeline". However, Ilastik+CellProfiler rely on manual annotations of nuclei, cell membrane and background to generate a probability mask, which is segmented by CellProfiler. This method can be challenging in dense tissues where the cells are packed close together, such as the lymph nodes. Bodenmiller also implements Mesmer, a pretrained deep learning model that achieves superior performance than ilastik (https://pmc.ncbi.nlm.nih.gov/articles/PMC9010346/). In fact, it was previously shown that Ilastik+CellProfiler segmentation will result in more "unassigned cells" than Mesmer (https://www.nature.com/articles/s41467-023-40068-5, supplementary figure 18), as the authors found in page 2 line 10 where they say they annotated almost all TDLN cells. Why did the authors decided to use Ilastik+CellProfiler over Mesmer? Segmentation is key to perform cell type annotation with Phenograph, so it would be very useful for the reader if the authors could provide some quality control of the segmentation, and/or overlay raw images with the segmentation masks.
3. Cell type annotation: Some annotations of the cell clusters obtained with Phenograph are not entirely clear. For instance, cluster CD8 act1has high expression of CD1c and SOX-10, which are not typical CD8 T cells markers. This is also true of the DC1 and DC2 cells which seem to have a mixture of markers usually associated with cDC1 and cDC2 e.g. CD141 and CD1c – making it hard to know what these assignments correspond to exactly. It will help the reader if the authors provide clarification of those clusters and a table with the markers expected per cell type, similar to the Sankey plot in Supplementary figure 2.f, but with the markers expected. It would also be helpful to see exemplar cells once they have been assigned.
4. In Supplementary figure 2.c, it seems like CD68 is expressed in most of the cells, which is not biologically accurate. It looks like a staining artifact. The annotation relies on the intensity of the markers, therefore, it will make the analysis more robust if the authors apply a method to normalise the contrast of the channels, such as contrast limited adaptive histogram equalization method (https://siril.readthedocs.io/en/latest/processing/clahe.html), or justify why there is no need to apply such adjustment.

5. The findings of dyads of DC and CD8 are important and interesting. They appear to have been determined by looking at cells within 15um of one another. Is this from the centroid of the cells or from their membrane as determined by the segmentation? Could approaches more commonly used in GIS be used to determine actual contact rather than distance between? This would more robustly show dyads. It would also be good to have representative images of the closer and

further spaced dyads to determine whether these represent actual contacts or proximity. This is relevant as the macrophages are <15-30um meaning many of those are as close as the cells within dyads which changes the interpretation of what these neigbourhoods may look like.

6. Could the authors apply some clustering approaches when looking at dyads to determine whether there is increased clustering of DC and CD8 T cells or whether there is an increase simply due to increased numbers of DC within these neighbourhoods? This also applies to the % of macrophages near a dyad. Is this simply that with the increased number of dyads most of the ROI lies within 30um of a dyad? It would, again, be good to see some representative images of this to clarify. It would also be useful to have more clarity about the y-axis as it appears to say % of cells in a dyad with a DC, if this refers to the percentage of CD8 T cells contacting DC then it seems unusual that there are many cells in contact with DC given the usual rarity of DC within a node.

7. The mouse experiments could do with some additional controls. The Pla2g2d-/- mice have slower growth but it is unclear if this is due to impacts of development etc. in these mice, given that the Pla2g2d is mainly expressed by myeloid cells would it be possible to repeat this in bone marrow chimeras so that the Pla2g2d-/- or wt bone marrow is placed into wildtype mice which will be less influenced by this and could be more robust? This section would also be improved by using a more relevant animal model for the study. Given the focus on metastatic recurrence it would be better to use the B16F10 resection model where the primary tumour is removed and metastasis is monitored. This could also be done using HCMEL or other metastatic lines. This would make this more relevant to the rest of the paper rather than switching to primary tumour growth.

8. In Fig 4f, could the authors test whether PD-1 blockade is significantly adding to the growth reduction of the 47.M2 addition? It is unclear whether this test was performed and it would be useful to interpret the conclusion that the 47.M2 with PD1 delayed growth considering that the 47.M2 is borderline significant alone? This would also be helped by displaying in the supplemental the individual growth curves to give an idea of spread and different growth trajectories.

9. Fig 3c could be discussed more clearly as the assertion that macrophages are more inflammatory seems likely, however, there are both IFN-Mac4(22) and IFN-Mac(07) which are downregulated. What leads to this discrepancy? Is there some more nuance to the interpretation needed?

10. Is there a reason for the lack of PD-1 expression within the PD-1 sorted CD8 T cells in Fig 3f? It seems unusual that sorted PD-1+ T cells would only be 20% PD1+ by this analysis.

11. For Fig 4a it would be helpful to do an influential point analysis for the correlation looking at the extreme outlier. Given the low number of points it seems possible that this is influencing the outcome of the analysis.

12. The description of the methods is vague. The methods, especially concerning image preprocessing e.g. denoising, could do with expansion.

13. For Supplementary Table 1 it would be good to clarify what the values for the age correspond to i.e. mean(range) or mean(some other interval), since the range wouldn't cover the subgroups (similar for the Breslow Mean).

14. In Figure 1 d, the heatmap label under the neighbours column is cut.

15. In Supplementary Fig 2 d, there are duplicated labels for patients 21 and 22.

16. Individual datapoints should be shown in 2e, 2i, 2h, 2j and 4a (it appears from 4b that this should be feasible given the n, however, it may be that this makes the graphs uninterpretable but this is a suggestion to aid in seeing raw data more clearly).

17. Some statistical analysis of Sup Figure 8d would be welcome as while it does seem to show that there is reduced proliferation of PD1hi cells there are several individuals where the PD1int seem to show similarly low proliferation. As such some unbiased statistical analysis would help demonstrate the point that these PD1hi persist less well.

Referee #3

(Remarks to the Author)
Krimpen et al claim that spatial analysis of tumor-draining lymph nodes from melanoma patients with and without early recurrence identified PLA2G2D as novel cancer immunotherapy target. In the first three figures they highlight the cell type annotations, their composition differences, cell interactions and transcriptional differences associated with recurrence and disease free status. They identify an immune-suppressive myeloid hub in the TDLN i.e., immunoregulatory DCs, macrophages and pre-exhausted CD8 T cells that is associated with disease recurrence. Further they show that PLA2G2D expressed by myeloid cells is likely to play an immunosuppressive role and claim that PLA2G2D loss-of-function or therapeutic inhibition markedly improved survival in tumor-bearing mice and increased the efficacy of anti-PD-1 immunotherapy.

Overall, this manuscript is largely descriptive with many hypothesis and requires extensive functional studies to support the central conclusions. My major concerns are outlined below:

Major concerns.

1. Lacks mechanistic and functional data: The major goal of their spatial analysis of tumor-draining lymph nodes (TDLN) is to understand the mechanisms that lead to recurrence of melanoma after surgical resection. Although spatial analysis of the TDNL has yielded interesting associations with recurrence, including a potential role of PLA2G2D, these are simply hypothesis generating experiments. These hypotheses need to be tested in relevant models to determine causal mechanisms underlying recurrence. Importantly, the authors fail to show that PLA2G2D expression in TDLN plays an important role in disease recurrence? The same holds true for the role of the immune-suppressive myeloid hub in the TDLN i.e., immunoregulatory DCs, macrophages and pre-exhausted CD8 T cells in disease recurrence. The functional experiments using Pla2g2d-deficienct mice or anti-PLA2G2D blocking antibody neither highlights a role of PLA2G2D in recurrence or assesses its function in modulating immune responses in the TDLNs. The sister paper (Umetani et al) also does not explore the role of PLA2G2D in TDLN or recurrence models. The authors should consider experiments in relevant tumor recurrence models to assess the role of PLA2G2D in TDLNs. In addition, selective depletion of specific immune subsets in these models is likely to clarify how recurrence is mediated by the interactions between immunoregulatory DCs, macrophages and pre-exhausted CD8 T cells in the TDNL.

2. Lack of clarity between disease recurrence and anti-PD1 resistance. In the method section on patient inclusion, the authors state that no patients received neo-adjuvant treatment. Also there is no mention of anti-PD1 therapy, although majority received adjuvant chemotherapy. Thus, the observations in their patients are limited to disease recurrence, presumably in the setting where immunotherapy is not given. If this is not the case the authors should provide details of immunotherapy post-surgery. It is puzzling as to why the authors have taken their results on association of PLA2G2D with disease recurrence to testing whether "PLA2G2D mediated resistance to immune checkpoint inhibition". Nevertheless, the results of their experiments does not clearly show that combined treatment (anti-PD1 or anti-PLA2GD2 antibody) when compared to monotherapy results in significant difference in tumor control; comparisons are made with isotype control (Fig. 4f). Furthermore, there appears to be no significant difference between anti-PD1 versus anti-PLA2G2D antibody treatment (Fig. 4f). In conclusion, the authors have not clearly demonstrated a mechanistic link between PLA2G2D and anti-PD1 resistance.

3. Failure to cross-compare findings in the tumor. If the authors want to highlight the importance of TDLN when compared to tumor per se, it is important to compare their finding in the tumor. Are there features in the tumor that predict recurrence? Are these feature absent in the TDLN or what is the correlation? Are mechanisms that are associated with recurrence different in the tumor and TDLN? I understand that TDLN are poorly studied, and that their analysis yielded interesting insights. Would they have captured these findings by analyzing the tumor by single-cell analysis or spatial analysis? In summary, the rationale to study TDLN without analysis of tumor is not clear.

4. Interpretation of gene expression findings in immune cells is incomplete.
(i) T cells in recurrence patients are called as pre-exhausted based on markers. However, based on enrichment of gene signatures Figure 3C (right panel) it is clear that tissue-resident memory (TRM) (ZNF683+ (Hobit)+) is the most enriched in T cells from recurrence patients. Given the established importance of TRM cells in anti-tumor immunity and responses to anti-PD1 therapy, it is a glaring omission that the authors have failed to assess this population. Recent work from David Masopust's group has shown that TRM cells can recirculate between tissues and draining lymph nodes. TRM cells and exhausted cells share several marker genes including PD1. If the cells increased in recurrence are mainly TRM? Then why are these cells present in the lymph node and interacting with macrophages and DCs in the TDLN? Are they dealing with silent metastasis? Are they tumor-specific or just bystander cells? Many open question are unanswered.
(ii) The macrophage population linked to recurrence is labelled as immunosuppressive? What is the evidence to support this? Several single-cell studies of myeloid populations in the tumor have described the presence of CXCL9 expression macrophages (i.e., interferon response signatures). These macrophages with interferon response signatures are linked to good anti-tumor outcomes as in PMID: 37535729. Thus, it is important to not overstate the functional properties based on a few molecules.

Gene expression studies only generate hypotheses, these need to be rigorously tested in functional models before making firm conclusions about mechanisms.

5. Tumor-specific T cell analysis is lacking. A major downside of the spatial analysis as opposed to single-cell transcriptomic and TCR-seq is the ability to track potential tumor-specific T cells and test their TCR specificities. However, expression of certain marker genes like CD39 have been linked to tumor-antigen-specific T cells. Do the authors find CD39 expression in CD8 T cells? if so these may represent tumor-specific T cells and likely to be functional important. In the absence of information on the tumor-specificity of T cells in the TDLN, it is difficult to draw mechanistic insights as these cells may represent bystander cells and not likely to play roles in anti-tumor immunity.

6. Annotations of immune cell types is incomplete. In figure 1, the annotations of many immune cell type is not complete. Labeling DCs as DRhigh and low, macrophages as CD163.1, CD163.2, CD8 as act1,act2,act3 is not adequate, there are several large-scale single-cell datasets, especially those from Miriam Merad, that have detailed accurate classifications of myeloid and DC subsets across many tumor types.

7. Sample size. Another weakness is the relatively small sample size 5-6 patients per group. Inter-donor heterogeneity is a problem. Generally hypothesis seeking studies need to have a larger sample size and donor specific data should be shown throughout as opposed to aggregate data/ group for gene expression comparisons etc.

Overall, this study is largely descriptive, preliminary and requires more work.

Version 3:

Reviewer comments:

Referee #1

(Remarks to the Author)
The combination of the 2 articles greatly improved the quality of the manuscript. It established that PLA2G2D is an immunosuppressive mechanism, quite likely dampening the immune response when inflammation is increased, as previously described in elderly people. During cancer, it clearly constitutes a break to the immune response, that can be released after genetic inactivation of blocking with an Ab. Some points from the first revision were answered but there are still some points that need to be addressed.
I am still not convinced by the statement that the CD8 T cells found in the TDLN are pre-exhausted. They are primed and express a panel of activation markers, including TOX that can be expressed in polyfunctional T cells in human (10.1126/sciimmunol.aba7918) or PD1, that is also expressed in a significant portion of Tem for instance. The in vitro assay just compares primed vs non primed T cells and does not inform whether these cells are pre-exhausted or not. Indeed, the transcriptomic analysis shows that the profile can be compared to the one of Tem or exhausted cells, not just to exhausted T cells. Do the authors find similar conclusions in the mouse models they used? It would be easier to study and to formally prove this point there. The authors established in Figure 3 that PLA2G2D has a direct immunosuppressive effect on T cells, by dampening T cell proliferation and activation in vitro. Does it increase TOX expression or features associated with exhaustion? In mouse model in vivo, the authors studied the effect of blocking Ab in various tumor models, with a focus on tumor specific T cells. The scRNAseq data on T cells (Fig 4O) shows that indeed the blocking of PLA2G2D leads to higher activation, and so by higher levels of exhaustion associated genes that are also expressed transiently upon activation (entpd1, pdcd1, Bhlhe40, Runx2,…). It is not clear to me whether this analysis was performed only on TDLN or combined both TDLN and tumor CD8 T cells. A focus on TDLN would clarify whether in absence of blocking, PLA2G2D promotes “pre-exhaustion” or not. Flow cytometry study on tumor specific CD8 T cells could also be performed.
The gating for PD-1 in Reviewer figure 1 is not appropriate. The gate should be slightly higher.
The authors used anti-CSF1R to deplete “tissue resident macrophages”. However, they did not show whether it indeed depletes these TAM from the tumors. Could they please confirm the efficiency of the treatment and the lack of effect on LNM? Most of the analysis was performed by checking RNA levels. It sometimes does not correlate with the protein expression.
I agree with the authors that in mice, the LMN population is quite likely the source of PLA2G2D, even if the authors did not perform a specific genetic inactivation in this population. However, in patients, it seems that the pattern of expression is a bit different with this expression in DCs. Additionally, one aspect that is not covered by the authors is the possible role of PLA2G2D in tertiary lymphoid structures. Could they find some expression of PLA2G2D in these structures, together with the formation of triads as described in TDLN in patients or is it really specific to TDLN.
The author used pla2gd2 -/- mice injected with various tumor cell lines (as previously done by another group). They do not discuss how this KO could affect the immune system in untreated mice. Does this KO have a general effect or does not display any phenotype?
If I understood well, PLA2G2D is also expressed in LN at steady states. Can the author make this point clearer. It is higher in elderly related to increased inflammation and was shown to be involved in tumor progression. Do they know whether it is also more expressed after infection? Is it a natural mechanism to dampen the immune system such as PD-1 expression?

Referee #2

(Remarks to the Author)
The study as presented represents a huge corpus of work. The key results have not changed, however the additional work has strengthened the conclusions. The spatial analysis of patient tumour draining lymph nodes is technically impressive and the analysis is enlightening. The data is analysed appropriately throughout. Concerns re. statistics have been addressed and the conclusions are robust and valid. I would not require any further revision to view this as a complete body of work ready for publication. The authors have done an impressive amount of work to address the concerns which were raised in the first round of review. As before, the paper is well written and clear.

Referee #3

(Remarks to the Author)
Krimpen et al have conducted a wide-range of experiments and analysis, including merging data from Umetani et al manuscript. All my major concerns have been satisfactorily addressed, and the new combined manuscript brings much needed mechanistic insights into the immunomodulatory role of myeloid-derived PLA2G2D in the tumor-draining lymph nodes (TDLN).

To summarize the authors have:
• Clarified why the recurrence models were not used.
• Included data from larger validation cohorts and a different cancer type (NSCLC).
• Compared findings in the TDLNs and tumor (Fig. 2), and showed that expression of PLA2G2D was highest in the CD11c+ DC and CD68+ Macrophages present in the TDLN, thus supporting a TDLN-specific checkpoint.
• Highlight interactions between PLA2G2D+CD11c+ DCs and CD8 T cells in TDLN from melanoma patients (Fig. 2).

• Included extensive mouse model data and functional studies from the Umetani et al manuscript to strength the mechanistic aspects (Fig.3-4).
• Added supporting data from a LN-metastatic melanoma model - highlighting concordant features with stage III melanoma.
• Identified a specific myeloid cell type with low CSF1R expression as the key cellular source of PLA2G2D.
• Defined the cell types mediating the immunosuppressive effects using bone marrow chimer and cell-depletions.
• Demonstrated effects of PLA2G2D-deficiency on tumor-specific T cells in vivo.
• Confirmed synergistic effects with anti-PD1 treatment.
• Included humanized mouse model data and human-specific antibodies to support translational relevance.

Although the mechanisms of PLA2G2D induction in myeloid cells, the nature of its cognate receptor, and down-stream signaling on T cells are not fully characterized, there is sufficient novelty and significance in the current body work presented in the revised manuscript.

Few minor points to consider:
1. Do the authors find the Pla2g2d-enriched macrophage population identified in mice TDLN (Fig. 4i,j) in human TDLN or tumors? Are they transcriptionally similar or different. A brief statement about this will be sufficient.
2. Most data in humans is derived from melanoma and NSCLC, do the authors speculate that this mechanism is generic to many human cancer types? if they have any data or even re-analysis of published data, it will be helpful to highlight this in the manuscript.

Recommend publication.

Version 4:

Reviewer comments:

Referee #1

(Remarks to the Author)
The authors answered all the points raised during the revision. I congratulate them for their work. I am satisfied with their answers.

Open Access This Peer Review File is licensed under a Creative Commons Attribution 4.0 International License, which permits use, sharing, adaptation, distribution and reproduction in any medium or format, as long as you give appropriate credit to the original author(s) and the source, provide a link to the Creative Commons license, and indicate if changes were made.
In cases where reviewers are anonymous, credit should be given to 'Anonymous Referee' and the source.
The images or other third party material in this Peer Review File are included in the article's Creative Commons license, unless indicated otherwise in a credit line to the material. If material is not included in the article's Creative Commons license and your intended use is not permitted by statutory regulation or exceeds the permitted use, you will need to obtain

permission directly from the copyright holder.
To view a copy of this license, visit https://creativecommons.org/licenses/by/4.0/

Point-by-point response letter – 2024-10-22118C (Van Krimpen *et al.*)

General Statement

We want to thank all reviewers for taking an interest in our work and for providing constructive feedback, which we believe has greatly improved the quality of our study. After consulting with the editor, we were advised to merge both manuscripts (original companion paper: Umetani *et al.*) and provide a single compact yet coherent story, including addressing all reviewer comments. **Major revisions include**:

- Re-analysis of the imaging mass cytometry data to validate cell segmentation and improve cell annotation in our tumor-draining lymph node (TDLN) dataset (**Fig.1**, **Supplementary Fig.1-4**);
- Extension of PLA2G2D detection to the non-metastatic LN patient setting and vice versa by in-depth analysis of the role of PLA2G2D in anti-tumour immunity in a LN-metastatic mouse model (**Fig.2**, **Fig.4-5**, **Supplementary Fig.10-12**);
- Identification of the primary source of PLA2G2D *in vivo* through bone marrow chimera generation, genetic/antibody depletion experiments and single cell transcriptomics (**Fig.4-5**, **Supplementary Fig.10-11**)
- Characterization of the mechanism of action of PLA2G2D-mediated suppression of (tumour-specific) $CD8^+$ T cells (**Fig.3-5**, **Supplementary Fig.10-11)**;
- New *in vivo* experiments demonstrating the non-redundancy of anti-PLA2G2D treatment with PD-1 checkpoint inhibition, emphasizing the therapeutic potential of combination treatment (**Fig.5**, **Supplementary Fig.12)**;
- Experiments using humanized *PLA2G2D*-expressing transgenic mice and our new human-specific PLA2G2D-blocking antibodies provide the groundwork for future clinical trials (**Fig.3**, **Fig.5**, **Supplementary Fig.12**).

Strengthened by these efforts, we are convinced that the TDLN-centered PLA2G2D checkpoint we described here – including the tools developed to therapeutically target it – offers exciting new potential for improving anti-tumor immunity in patients. Please find below our detailed responses to the reviewer's comments **in blue**. All reviewer comments linked to data transferred from the Umetani *et al.* manuscript (and that did not substantially overlap with comments for the Van Krimpen *et al.* manuscript) were also included in this response letter. For your convenience, we have added versions of the new manuscript and supplementary data in which all changes made during the revision period are highlighted in yellow.

Reviewer #1

Authors from this article studied Tumor Draining Lymph Nodes from stage II (non-metastatic) and III (metastatic) melanoma patients using spatial analysis. They concluded that patients who undergo recurrence within 2 years display specific cellular interactions in the paracortex region of the DLN and involving Tregs, DC, macrophages and CD8 T cells. They state that these CD8 T cells are poised for exhaustion and that this state is associated with chronic IFN signaling. Furthermore, they established that DC and macrophages from these areas express high levels of PLA2G2D. Study of tumor growth in PLA2G2D full KO mice or by systemic blocking using an Ab, showed a negative effect on tumor growth. Altogether, there are some interesting data in this study, especially for the effect of the PLA2G2D blocking on B16 melanoma, but also some overstatements, a lack of mechanistic studies to explain the effect of PLA2G2D blocking and the role of the cell clusters observed in TDLN from recurrent patients. This lack of mechanisms weakens the link between the 2 stories from this study with the spatial analysis on one side and the part focusing on PLA2G2D on the other.

Response: We agree with the reviewer that our initial plan of separating the two bodies of work did not allow for making full use of the complementary nature of the two studies. We have now integrated both studies and added substantial additional mechanistic findings to provide a more coherent and solid narrative.

Major concerns:
1. Although the initial study covered the analysis of stage 2 and 3 melanoma patients, it appears that only stage 3 patients display differences between their DC/Mac ratio between DF and R patients. After figure 2e, all the study focuses only on stage 3/metastatic patients but not stage 2 patients. This should be emphasized in the abstract as it restricts the potential interest of this discovery.

Response: The reviewer raises a fair point, which has led to additional experiments and this point being included in the discussion section of the manuscript (**page 5**). Initially, we focused primarily on stage 3 patients because of the more pronounced contextual differences and poorer outcomes/higher clinical unmet need in this group. However, given the efficacy of anti-PLA2G2D in non-metastatic mouse models, we hypothesized that although the DC/Mac ratio in stage 2 patients differs from the metastatic stage 3 setting, induction of PLA2G2D expression in TDLNs may still impact anti-tumor immune responses in stage II patients. To test this, we performed additional experiments on samples from stage 2 melanoma patients and in mouse models of LN-metastatic/non-metastatic melanoma. Indeed, imaging analysis of TDLNs from an independent patient cohort revealed a substantial and similar induction of PLA2G2D levels in dendritic cells and macrophages from both stage 2 and stage 3 patients (**Fig.2g**), with equally striking interaction frequencies observed between PLA2G2D$^+$ myeloid cells and (PD-1$^+$) CD8$^+$ T cells in both patient groups (**Fig.2h**). Moreover, anti-PLA2G2D antibody therapy showed efficacy in both LN-metastatic (LN6-987AL) as well as non-TDLN metastatic (B16F0/B16F10) melanoma tumor models (**Fig.3-5**). Together, these findings support the notion that the TDLN-centered PLA2G2D checkpoint we uncovered is active and therapeutically targetable independently of TDLN metastases.

2. Furthermore, this suggests that the presence of tumor cells in the TDLN is required to induce the phenotype observed, i.e. the presence of complexes of cells composed of macrophages, DCs, Tregs and CD8 T cells. Even though the authors studied the paracortical area away from the tumor, tumor cells are still residing in the same LN than the T cells studied, and it is quite likely that these T cells are in the end quite similar to the ones found in the primary tumor or in the vicinity of the primary tumor. A direct comparison with primary tumor samples would be of major importance to prove that this cluster of cells and their state is specific to the TDLNs from recurrent patients and not just a common feature to cells found in the tumor.

Response: We agree that relating our TDLN findings to the primary tumor is important. To this end, we first conducted imaging analyses of TDLNs, TDLN-metastases and primary tumors from an independent patient cohort. This revealed a substantial and similar induction of PLA2G2D levels in dendritic cells and macrophages from both stage 2 and stage 3 patients (**Fig.2g**). PLA2G2D$^+$ myeloid cell abundance was much higher in TDLNs than in primary tumors, irrespective of the presence of a TDLN metastasis – the latter in fact showing the lowest levels of PLA2G2D$^+$ macrophages and DCs (**Fig.2g**). Moreover, co-localization analysis revealed substantially increased interactions between PLA2G2D$^+$ myeloid cells and (PD-1$^+$) CD8$^+$ T cells in TDLNs as compared to the (primary) tumor, again independently from the presence of TDLN metastasis (**Fig.2h**). Finally, newly generated single-cell transcriptome analyses of TDLNs and primary tumors from mouse models of (LN-metastatic) melanoma revealed that *Pla2g2d* expression was confined to a specific macrophage population. These macrophages were especially frequent in TDLNs but also resided in non-TDLNs, whereas *Pla2g2d*-expressing cells were absent in tumor samples (**Fig.4h-m, Supplementary Fig.S10i**). Moreover, *Pla2g2d*-expressing macrophages appeared just prior to when anti-PLA2G2D therapeutic effects became apparent (**Fig.4b, Fig.4g)**. Furthermore, *Pla2g2d* expression by LN-macrophages was similar in both metastatic (LN6-987AL) and non-metastatic (B16F0) models, suggesting that *Pla2g2d* induction is independent of direct tumor cell proximity (**Supplementary Fig.S10l**). Together, these results – while they do not formally exclude an effect of PLA2G2D in the tumor microenvironment – support the concept of a (TD)LN-centered PLA2G2D immune checkpoint (now discussed on **page 5-6**).

3. The author states that the CXCL13+ CD8 T cells found in these clusters are poised for T cell exhaustion. This is not proven in the paper. First, the authors used tumor free TDLN for the study of PD1+ or naïve CD8 T cells, although they showed that the immunosuppressive myeloid cells that discriminate DF from R are found in stage 3 but are not discriminant in stage 2 patients. The term poised is related to an epigenetic imprinting of the cells, which is not studied here. Furthermore, their conclusions are drawn from 5 days "chronic" stimulation from naïve or PD1+ sorted CD8 T cells. The initial protocol cited by the author used a stimulation other 10 days (changing CD3 coated plates every other day). A 5 days stimulation, even when changing plates every day, is not that different than a classical stimulation. In that case, it is surprising to see that barely any naive stimulated T cell express PD-1 or TIM-3, that are also activation markers. Are the cells activated? Any activation markers are expressed? The slight increase of some markers is not sufficient to conclude to a poised exhausted state.

Response: We agree with the reviewer that the term 'poised' was confusing here, as we did not intend to indicate epigenetic alterations (which we indeed did not study). We have changed the text accordingly (**page 3**).

Regarding the exhaustion assay: the original protocol published by Belk *et al.* (PMID: 35750052) lasts 10 days and starts from a pure population of naive (murine) T cells, leading to ~50% PD-1⁺TIM3⁺ cells at the end of culture, which bear an exhausted T cell transcriptional signature. Since we aimed to assess the induction of exhaustion in already primed (i.e. non-naïve) PD-1⁺ cells as compared to naïve T cells from TDLNs, we chose a shorter stimulation since the PD-1⁺ cell population was already activated and co-expressed inhibitory receptors and TOX, while still lacking TIM-3 (**Supplementary Fig.8c**). The 5-day culture protocol was based on pilot experiments in which we already observed CD69 and EOMES expression on NSCLC TDLN naïve CD8⁺ T cells at day 4 of culture. However, TIM-3 only became upregulated on PD-1⁺ CD8⁺ T cells (see **Reviewer Fig.1a** below). Combined with the strong enrichment of known exhausted T cell transcriptional signatures (now also including the pan-TEX signature recently published by the MacKay lab, see **Supplementary Fig.8a**) and the increased levels of exhausting-related proteins such as TOX determined by IMC (**Supplementary Fig.4e**), we believe it is reasonable to conclude that paracortex CD8⁺ T cells in R patients have activated a transcriptional program linked to a (pre-)exhausted T cell state. Nevertheless, 'proneness' to exhaustion is a poorly characterized concept and we did not perform in-depth molecular and functional characterizations in sufficient numbers of samples to formally conclude this. Hence, we added nuance to our conclusions (**page 3, page 5**).

The reviewer is also correct in stating that percentages shown in the original Fig.3f were rather low. When re-visiting our flow cytometry data, we noticed that PD-1^{dim} cells were not included in our PD-1+ gate. Taking these cells along in a re-analysis revealed substantially higher percentages of positive cells (e.g. from 20% to >50% PD-1+ non-naive cells; see new **Supplementary Fig.8e**), more in line with what one would expect after 5 days of stimulation (new gating is shown in **Reviewer Fig.1b**).

Reviewer Figure 1

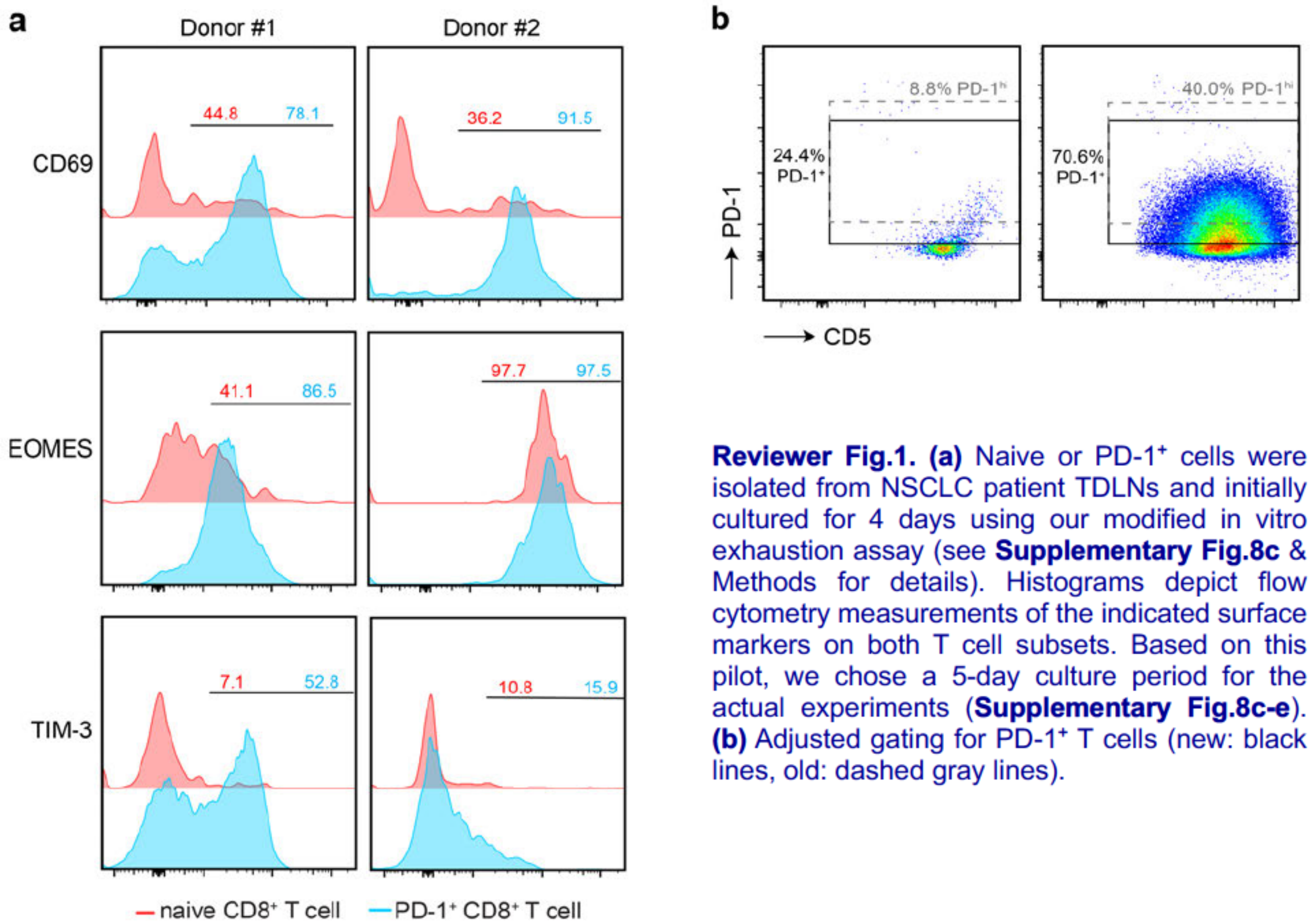

Reviewer Fig.1. (a) Naive or PD-1⁺ cells were isolated from NSCLC patient TDLNs and initially cultured for 4 days using our modified in vitro exhaustion assay (see **Supplementary Fig.8c** & Methods for details). Histograms depict flow cytometry measurements of the indicated surface markers on both T cell subsets. Based on this pilot, we chose a 5-day culture period for the actual experiments (**Supplementary Fig.8c-e**). **(b)** Adjusted gating for PD-1⁺ T cells (new: black lines, old: dashed gray lines).

4. Effect of the KO of pla2g2d on tumor development was already described in (Miki et al JBC 2016) and pan cancer TCGA analysis also pointed out to the potential use of pla2g2d as marker for anti-PD1 resistance (Yang et al, Nat Comm 2021). These 2 articles should be cited in the article.

Response: We thank the reviewer for mentioning these relevant papers, both of which were cited in our initial manuscript (refs. 24 & 35) and again in the revised version (refs. 28 & 37).

5. In figure 4, the authors validated that PLA2G2D protein is more expressed in TDLN from recurrent patients compared to disease free patients from stage 3 patients. The authors also mentioned that intratumor PLA2G2D was not significantly different between DF and R patients. One unanswered question is whether the blocking of PLA2G2D is important in the TDLN or in the tumor.

Response: This is indeed an interesting question. Our combined data, in particular the newly acquired imaging and single cell RNA-Seq analyses, provides several strong arguments in favor of a more dominant – but not necessarily exclusive – effect of PLA2G2D inhibition in the TDLN. First, we have shown consistently higher expression of PLA2G2D mRNA and protein in TDLNs compared to (primary) tumor samples (**Fig.2e**, **Fig.2g**, **Supplementary Fig.9**). Second, single cell transcriptome analyses of B16 melanoma-bearing mice showed that *Pla2g2d* expression is confined to a LN-specific macrophage subset that was expanded in TDLNs and largely absent from tumors (**Fig.4h-m**). Importantly, this (TD)LN-specific expression of *Pla2g2d* at day 12 post tumor cell inoculation (p.i.) closely aligned to the time window (day 10-13 p.i.) when anti-PLA2G2D treatment (initiated at day 8 p.i.) induces tumor-specific $CD8^+$ T cell expansion and starts to inhibit tumor growth (**Fig.4b**, **Fig.4g**, **Fig.5a-c**). Nevertheless, as PLA2G2D can be detected (albeit at lower levels) in patient tumors, we envision that depending on the type of cancer and phase of tumor development PLA2G2D activity may also be induced in the tumor microenvironment – e.g. in response to chronic pro-inflammatory signals. These considerations are now discussed in the revised manuscript (**page 5-6**).

6. Figure 4 C: A co-staining with CD11b/CD11c would help to pinpoint which cell types expresses PLA2G2D.

Response: We agree that this is important, and we have now added co-stainings of PLA2G2D with DC, macrophage and T cell markers (**Fig.2g-h**). These experiments demonstrate that PLA2G2D expression is detected in macrophages as well as DCs but not in $CD8^+$ T cells – in line with our spatial transcriptomics findings (**Fig.2b, Supplementary Fig.7**). Our newly added single cell transcriptome analyses of B16 melanoma-bearing mice confirm the myeloid origin of PLA2G2D, pinpointing *Pla2g2d* transcripts to a LN-specific macrophage subset that is highly abundant in (TD)LNs but largely absent from tumors (**Fig.4h-m, Supplementary Fig.10i**).

7. Figure 4 D-F: In mouse, the authors showed that the KO or the blocking of PLA2G2D favor tumor growth but do not describe further any effect on the immune system. Many questions should be answered concerning the mechanisms in place. Is blocking important within the tumor itself or in the TDLN? Are macrophages (or DC) really the source of PLA2G2D? A targeted KO should be done on both subtypes to demonstrate this point. Are the cellular interactions found in patients' TDLN also found in mice? Are they disrupted when PLA2G2D is blocked? Are tumor specific CD8 T cells more active/numerous within the tumor upon treatment? What is the general effect on immune cells? Answering these questions would help to render the study less descriptive and more impactful.

Response: We agree with the reviewer that more in-depth analyses of the *in vivo* effects of anti-PLA2G2D would yield mechanistic insights and increase the impact of our study. We therefore conducted a new series of *in vivo* experiments, including single cell transcriptomics and detailed analyses of how PLA2G2D inhibition affects anti-tumor immunity in a model of LN-metastatic melanoma (B16F0-derived LN6-987AL line) using flow cytometry (new **Fig.4-5**). Anti-PLA2G2D treatment resulted in (transiently) increased levels of proliferating memory $CD8^+$ T cells in the TDLN, blood and tumor of melanoma-bearing mice – including the improved induction of tumor-specific T cell responses (**Fig.5a-c**). Moreover, transcriptional programs linked to T cell activation were upregulated upon PLA2G2D inhibition (**Fig.4o**). Regulatory T cell proliferation was not substantially affected by PLA2G2D-blocking antibodies (**Fig.5d**). [redacted]

Bone marrow chimera experiments using PLA2G2D-deficient mice revealed a hematopoietic origin of PLA2G2D, excluding a stromal contribution to the observed therapeutic effect (**Fig.4d-f**). As detailed in our responses to comments #5 & #6 above, single cell transcriptomics experiments have identified LN-specific macrophages as the source of PLA2G2D *in vivo*, which are highly abundant in TDLNs but largely absent from tumors (**Fig.4h-m**). This subset of $PLA2G2D^+$ LN macrophages expressed low levels of *CSF1R*, encoding the CSF-1 receptor critical

for most macrophages. [redacted] Importantly, TDLN-specific expression of *Pla2g2d* closely aligned to the time window (day 10-13 post tumor inoculation) when anti-PLA2G2D treatment induces tumor-specific $CD8^+$ T cell expansion and starts to inhibit tumor growth (**Fig.4b, Fig.4g, Fig.5a-c**). PLA2G2D inhibited T cell proliferation and effector cytokine production *in vitro* (**Fig.3c-d**). Together with new data showing consistently higher expression of PLA2G2D mRNA and protein in TDLNs compared to (primary) tumor samples (**Fig.2**), these findings support our model of PLA2G2D as a myeloid TDLN-centered immune checkpoint that can be exploited for cancer immunotherapy.

Minor concerns:
1. After figure 2g, the authors focused on the neighborhood cluster 2. However, in supplementary figure 3, neighborhoods 1, 6 and 8 are also significantly different. Could the author also discuss these clusters briefly?

Response: We have added a brief discussion of neighborhoods 1, 6 and 8 to the revised manuscript (**page 2**).

Comments on data integrated from Umetani et al.

The mouse study to characterize the effect of systemic inactivation of Pla2g2d on the immune system during an anti-tumor response is not detailed enough. In general, numbers (normalized with the volume or the weight of the tumor) should be shown. More specifically, a more detailed analysis is required for all subsets of cells:
- Please include the gating strategy and representative dot plots for the various immune populations

Response: We agree and have conducted a set of new *in vivo* experiments using the LN-metastatic LN6-987AL melanoma model to provide a more detailed characterization of the impact of systemic PLA2G2D inhibition on anti-tumor immunity, consisting of flow cytometry and single cell RNA-sequencing experiments (**Fig.4-5**, **Supplementary Fig.10-11**). Representative plots of flow cytometry gating were added to the revised manuscript (**Fig.5b**, **Fig.5e**).

About figure 1D, the authors wrote "Pla2g2d-/- mice further attenuated the growth of MC38 tumors compared to WT mice in the presence of anti-PD1". Which statistical test has been performed between the 2 conditions? The difference is quite minor for the average tumor volume. Furthermore, there is no difference between the KO with or without anti-PD-1. Could the author discuss this point?

Response: Differences in tumor volume between *Pla2g2d* wildtype and knockout anti-PD-1 treated groups were tested using a two-way ANOVA with Fisher's LSD, showing statistical significance ($P < 0.05$; **Fig.5f**). To further substantiate this finding, we added experiments combining antibody-mediated PLA2G2D and PD-1 inhibition in *PLA2G2D*-humanized mice (**Supplementary Fig.12f**), which also showed a statistically significant reduction in tumour weight in the combination treatment group as compared to both monotherapies ($P < 0.05$; two-way ANOVA with Fisher's LSD). MC38 is an immunogenic tumor type known to respond well to anti-PD-1 monotherapy, and we observed similar outcomes with genetic deletion or antibody-mediated blockade of PLA2G2D (**Fig.3a**, **Fig.3g**, **Supplementary Fig.12f**). We reason that in such a tumour context, relatively little room remains for both modalities to additively boost $CD8^+$ T cell responses against the tumor even further. This explains the modest (yet reproducible) decrease in tumor growth we observed when treating *Pla2g2d* knockout animals with anti-PD-1 antibodies. This is quite different from tumors resistant to anti-PD-1 therapy: in B16F10 tumors for example, we observed a strong synergistic effect of anti-PD-1 and anti-PLA2G2D antibody treatment, with both monotherapies showing little effect (**Fig.5h**). We now discuss this point on **page 6** of the revised manuscript.

Figure 1E: Please show the statistical test between KO with control Fc and KO with PLA2G2D-Fc. In this setting, the analysis of the immune microenvironment would also help to understand how PLA2G2D is acting on the immune system.

Response: We have added the requested statistics to the plot (two-way ANOVA with Fisher's LSD, $P < 0.05$; now **Fig.3b**). While we agree that further analysis of the immune microenvironment in these mice would be interesting, we decided to prioritize our resources towards an in-depth analysis of the impact of PLA2G2D in the

more therapeutically relevant setting of PLA2G2D inhibition (**Fig.4-5**). We believe that following this orthogonal strategy has also provided substantial new insight into how PLA2G2D acts upon the immune system.

It is interesting to see that the effect of PLA2G2D is independent of its enzymatic activity. However, it is frustrating that no potential binders on T cells, but also on monocytes or DCs as the author showed an effect on these cells too, are tested or at least mentioned in the manuscript.

Response: We agree that this is an interesting direction to pursue. When prioritizing our resources, we decided to focus on several other key aspects of the PLA2G2D mode of action: 1) pinpointing the tissue-specific cell subsets that produce PLA2G2D in patients and *in vivo* (revised **Fig.2**, new **Fig.4**); 2) elucidating the impact of PLA2G2D activity on (tumor-specific) T cell responses (new **Fig.3-5**); and 3) investigating the potential of combining PLA2G2D inhibition with PD-1 blockade (new **Fig.5**). Identifying the putative PLA2G2D receptor will require a large body of additional work, which we believe extends beyond the scope of the current paper and its figure space constraints. Important to note is that PLA2G2D strongly binds heparan sulfate proteoglycans (PMID:10383420), which must be considered and will significantly complicate efforts to identify PLA2G2D receptors.

Figure 2B, C: In figure 2A, the authors showed that PLA2G2D decreases proliferation of stimulated T cells. Less proliferation can explain the decrease in IFNg and IL-2 measured in figure 2B and C. Please perform intracellular flow cytometry analysis to answer this point.

Response: The reviewer is correct – this was an important caveat to our initial analysis. We have performed the requested intracellular flow cytometry experiments, which revealed that PLA2G2D exposure reduces IFNγ and IL-2 production by activated T cells on a per cell basis. These data were added to the revised manuscript (**Fig.3d**).

Figure 5: To formally prove that IFNg and/or TNF are inducing PLA2G2D production in the tumor, in vivo blocking experiment (or using KO) should be performed.

Response: Based on our *ex vivo* spatial transcriptomics signatures (**Fig.2c**, **Supplementary Fig.6**), our *in vitro* findings (**Supplementary Fig.10c-d**) and public database mining (**Supplementary Fig.10e**), we hypothesized that IFNγ signaling may drive PLA2G2D induction in tissues. We have conducted the requested *in vivo* experiments and observed that IFNγ-blockade alone was not sufficient to significantly dampen *Pla2g2d* induction *in vivo* in the LN6-987AL melanoma model (**Supplementary Fig.10j-k**). These findings point at possible redundancy with other microenvironmental signals or cues (e.g. oxidative stress; PMID: 26392224). This has been added to the discussion section of the manuscript (**page 5**).

Figure 6: A-C: When are IL-2 and IFNg concentrations measured? If after 48 hours, is there also an effect on proliferation that could account for the difference in cytokine levels? A phenotyping of the cells would also be appreciated.

Response: Please see our response to the previous comment; intracellular flow cytometry shows that PLA2G2D affects cytokine production in individual T cells (**Fig.3d**). Hence, effects on proliferation do not account for the reduced cytokine output measured in these experiments (both measured at 48h; now shown in **Fig.3c**). Additional phenotyping was performed as requested, revealing that in contrast to proliferation and effector cytokine production, known activation markers (e.g. CD69, CD25) and co-inhibitory receptors (e.g. TIGIT, NKG2A, PD-1) were only modestly affected by PLA2G2D (**Reviewer Fig.2**).

Reviewer Figure 2

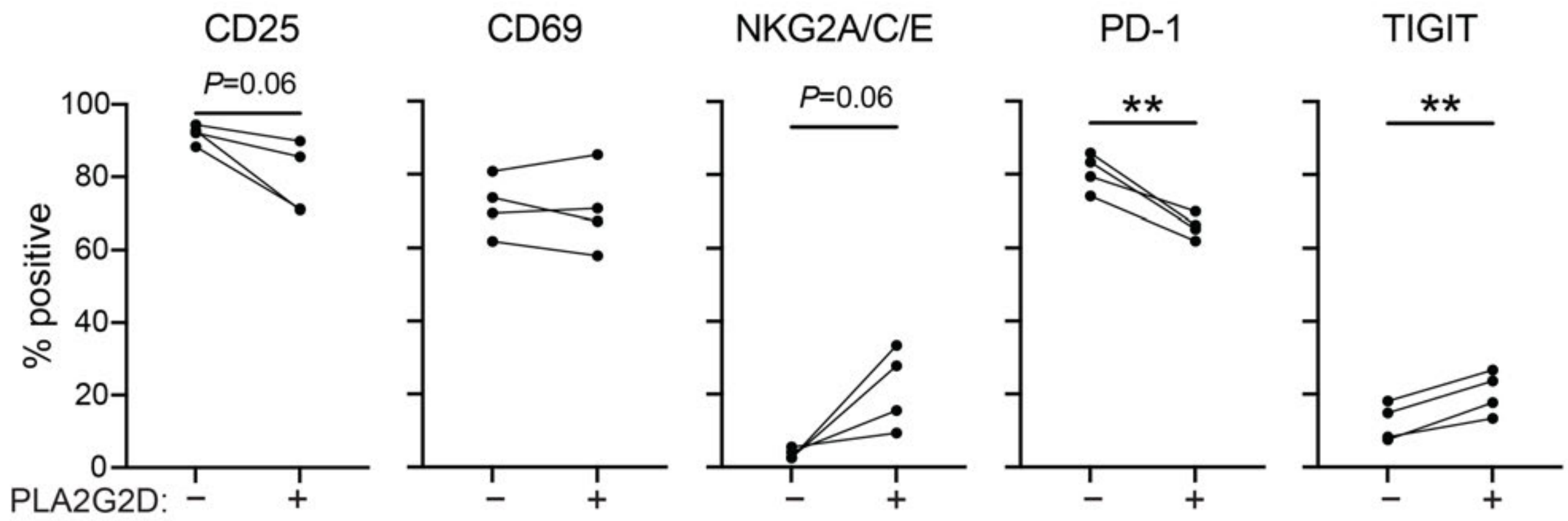

Reviewer Fig.2. Flow cytometry analysis of surface marker levels on CD8+ T cells isolated from healthy donor PBMCs in the presence or absence of PLA2G2D-Fc (10 μg/ml). T cells were activated using anti-CD3/CD28 stimulation at the start of culture, analysis was conducted after 72h of incubation.

<u>*Minor concerns:*</u>

For Q-PCR: what is the housekeeping gene used? What does fold change mean in the figures?

Response: Beta-actin (ACTB) and Hypoxanthine phosphoribosyl transferase (HPRT) were used as dual normalization genes for qPCR, based on their reported stability in human macrophages (PMID: 21122122). The geometric mean of the Ct values for ACTB and HPRT were used as the normalization factor for each sample to calculate relative expression between different samples and treatments. Regarding fold changes: expression of the reference baseline sample (i.e. untreated cells) was set to 1 for calculating fold changes of the other conditions. We have added this information to the Methods section of the revised manuscript (**page 24**).

Figure 5B: does the combined IFNg+TNF blocking increase the inhibition of pla2g2d RNA?

Response: We have not tested this specifically with the blocking antibodies. However, given the observations of additive effects when administrated as cytokines, it seems likely that combined IFNγ/TNFα inhibition would further suppress *PLA2G2D* RNA levels (**Supplementary Fig.10d**).

<u>Reviewer #2</u>

The authors present a technical tour-de-force profiling the organisation of tumor draining lymph nodes from patients with stage II or stage III melanoma who had either progressed or shown regression free survival following treatment. This led to them identifying changes in organisation within the paracortex which correlated with patient outcome. Interestingly this seemed to relate to the presence of interactions between CD8 T cells and specific myeloid subsets. They then identify PLA2G2D as being expressed highly by the myeloid cells present in these neighbourhoods and investigate this showing that high numbers of PLA2G2D+ cells correlate with recurrence. Finally they show that inhibition of PLA2G2D could lead to reduced tumor growth and sensitisation to PD1 blockade in the B16F10 model of melanoma. Given the relatively recent increased appreciation for the importance of the tumor draining lymph node in driving immunotherapeutic responses in vivo this is an important further development and highlights the potential that lies within studying the organisation of the node in defining therapeutic directions. While PLA2G2D has been previously implicated in control of immune responses in other settings, including cancer, this was the first time looking in a tumor draining lymph node and, given the findings, is highly significant. The paper is written clearly and concisely, with appropriate references to previous work and the conclusions appear robust. There are some concerns, however, needing addressing:

Response: We thank the reviewer for the very positive remarks regarding our study's methodology, writing and its conclusions.

Issues:

1. Image preprocessing: The authors state "To ensure data integrity, batch correction was then applied to the data per ablation-run, as well as Ki-67 expression, using the fastMNN method". However, one can still observe some batch effects on the UMAP shown in Supplementary Figure 2.d (e.g. patients 19 & 21). On the other hand, raw images suffer from several sources of technical noise that affect the counts of each channel (protein, DNA), which mask the biological signal and hinder following analysis steps, such as hot pixels and shot noise. Did the authors consider such sources of noise? It will make the analysis more robust if the authors present some quantitative metrics to assess image quality, such the SNR (signal to noise ratio).

Response: These are very relevant questions, and we regret not providing a more detailed description of our computational IMC analysis workflow. Regarding batch correction using fastMNN: we acknowledge that some minor batch effects may still remain, but we are hesitant to over-correct for such effects in fear of at the same time losing part of the biological differences between samples – especially if one employs subsequent orthogonal validation strategies as we have done. Regarding technical noise, we have used the following strategy (as developed by the Bodenmiller laboratory and frequently implemented by others, see Windhager *et al.* PMID: 37816904). Hot pixels were filtered by comparing the intensity of each pixel against the maximum intensity of the 3x3 neighboring pixels; if differences were larger than a threshold of 50 (i.e. default settings) then pixel intensity was clipped to the maximum intensity in the 3x3 neighborhood. Shot noise was (largely) filtered by performing all downstream analyses using median signal intensities per cell, with cells consisting on average of 80-90 pixels/µm² – minimizing the impact of rare random positive pixel artefacts. As a quantitative metric of image quality, we did indeed generate SNR (signal-to-noise ratio) values for all markers in our panel (now added in **Supplementary Fig.1b**). This shows that most hallmark markers for cell type identification (e.g. CD4, CD8, CD20, CD68, CD11c) showed relatively strong signal intensities and SNR (>2) values. As expected, transcription factor and specific cell surface receptor staining showed lower intensities, yet all with relatively high SNRs (>1.5). Finally, we calculated for each image the percentage of area covered by cells and inspected images with <50% cell coverage. We have now expanded the Methods section of the revised manuscript accordingly (**page 17-19**).

2. Cell segmentation: The authors performed the segmentation using Ilastik and CellProfiler, "in accordance with the Bodenmiller IMC segmentation pipeline". However, Ilastik+CellProfiler rely on manual annotations of nuclei, cell membrane and background to generate a probability mask, which is segmented by CellProfiler. This method can be challenging in dense tissues where the cells are packed close together, such as the lymph nodes. Bodenmiller also implements Mesmer, a pretrained deep learning model that achieves superior performance than ilastik (https://pmc.ncbi.nlm.nih.gov/articles/PMC9010346/). In fact, it was previously shown that Ilastik+CellProfiler segmentation will result in more "unassigned cells" than Mesmer (https://www.nature.com/articles/s41467-023-40068-5, supplementary figure 18), as the authors found in page 2 line 10 where they say they annotated almost all TDLN cells. Why did the authors decided to use Ilastik+CellProfiler over Mesmer? Segmentation is key to perform cell type annotation with Phenograph, so it would be very useful for the reader if the authors could provide some quality control of the segmentation, and/or overlay raw images with the segmentation masks.

Response: We fully agree that segmentation is a critical step that needs to be well controlled – particularly in cell-dense tissues such as lymph nodes. We chose the Ilastik+CellProfiler segmentation strategy mostly for practical reasons (i.e. well-documented, used by collaborating research groups). Hence, we appreciate the reviewer's suggestion to try and improve this through Mesmer, which at the same time allows us to validate key findings using an alternative segmentation approach. We re-segmented our IMC dataset using the deep learning approach offered by Deepcell's Mesmer, as implemented by the Steinbock pipeline, and compared key outcomes with our manual Ilastik+CellProfiler segmentation. After cell annotation, we observed only minor differences in cell type composition for both methods (**Supplementary Fig.3c**). The number of undefined cells was indeed lower using Mesmer, although both methods yielded negligible proportions (Ilastik+CellProfiler: 0.07%, Mesmer: 0.0014%). Importantly, the nearly identical outcomes from both methods translated in very similar changes in cellular abundance between both patient groups – including increased DC and reduced macrophage levels in recurrence patients (**Supplementary Fig.3d**). These data indicate that our initial segmentation is reproducible

and reliable, and were added to the manuscript (including detailed description in the Methods, **page 17-18**). To provide further insight to the reader in the performance of our Ilastik+CellProfiler segmentation, we have added a representative overlay image of raw IMC signals with the segmentation mask in **Supplementary Fig.1c**).

3. Cell type annotation: Some annotations of the cell clusters obtained with Phenograph are not entirely clear. For instance, cluster CD8 act1has high expression of CD1c and SOX-10, which are not typical CD8 T cells markers. This is also true of the DC1 and DC2 cells which seem to have a mixture of markers usually associated with cDC1 and cDC2 e.g. CD141 and CD1c – making it hard to know what these assignments correspond to exactly. It will help the reader if the authors provide clarification of those clusters and a table with the markers expected per cell type, similar to the Sankey plot in Supplementary figure 2.f, but with the markers expected. It would also be helpful to see exemplar cells once they have been assigned.

Response: We agree with the reviewer that in our initial phenotyping efforts we were too optimistic about the ability of our 40-marker panel to accurately discriminate between certain myeloid cell subsets and cell activation states. We have therefore revisited our Phenograph-based annotations, this time omitting too narrow state definitions and avoiding generating small subsets of cells with complex marker expression patterns. For these reasons, we refrained from using markers with relatively low signal intensity and SNR values (**Supplementary Fig.1b**) for phenotyping (e.g. BCL6, PD-L1). To increase annotation accuracy, we now limited definitions of key cell types to naive and non-naive cells for T cells (based on CD3, CD4, CD8A, FOXP3, CD45RA, KI-67, CTLA-4, PD-1 & HLA-DR), dendritic cells (CD11c^{hi}CD68^{lo}) separated based on high or low HLA-DR levels and macrophages (CD11c^{lo}CD68^{hi}) separated based on high levels of CD163 or CD169 (revised **Fig.1b-c**). We have updated the accompanying Sankey plot (**Supplementary Fig.2f**) and added the requested expected marker profiles to the revised Methods section (**page 18**). We would like to stress that cell segmentation and as a result annotation remain challenging in very cell-dense tissues such as lymph nodes, which exist by virtue of facilitating dynamic cell-cell interactions. Hence, some signal spillover because of (biased) cellular proximity in (TD)LNs appears unavoidable in our opinion (e.g. low-level T cell marker signals in DCs). We therefore built in several rounds of visual inspection and subsequent parameter optimization (e.g. marker-based gating of individual cell phenotypes using cytomapper) to maximize the accuracy of our final segmentation-annotation outcomes. As requested by the reviewer, we have added annotation examples to **Supplementary Fig.1d** and have thoroughly expanded our Methods section (**page 17-19**).

4. In Supplementary figure 2.c, it seems like CD68 is expressed in most of the cells, which is not biologically accurate. It looks like a staining artifact. The annotation relies on the intensity of the markers, therefore, it will make the analysis more robust if the authors apply a method to normalise the contrast of the channels, such as contrast limited adaptive histogram equalization method https://siril.readthedocs.io/en/latest/processing/clahe.html), or justify why there is no need to apply such adjustment.

Response: We thank the reviewer for bringing this to our attention. The CD68 UMAP projection indeed gives the impression of relatively high background signal, in contrast to visual inspection of CD68 signals in raw images (e.g. **Supplementary Fig.1d**, **Fig.1e**, **Fig.1j**), the SNR value analysis (**Supplementary Fig.1b**; with CD68 showing similar performance to CD11c, CD3 and CD45) and when plotted as relative signal intensities across cell types (**Fig.1c**). Hence, the Z-score scaled signal intensities in the UMAPs are not an entirely accurate reflection of IMC signal distributions. Importantly, because our cell annotation strategy relies on combined positivity/negativity for a combination of markers and on visual confirmation, the low-level CD68 signal arising from macrophage morphology or neighboring cells has minimal impact on the cell annotation. More specifically, our current macrophage annotation relies on combined positivity for CD68 and the macrophage-specific markers CD163 or CD169, which show quite specific staining patterns – also in UMAP representations (added to **Supplementary Fig.2e**). We appreciate the reviewer's suggestion to use contrast-improving strategies, but for the reasons mentioned above, we believe that the considerable time investment of a full re-do of data normalization and downstream analyses would very likely offer only limited benefits at this stage.

5. The findings of dyads of DC and CD8 are important and interesting. They appear to have been determined by looking at cells within 15um of one another. Is this from the centroid of the cells or from their membrane as

determined by the segmentation? Could approaches more commonly used in GIS be used to determine actual contact rather than distance between? This would more robustly show dyads. It would also be good to have representative images of the closer and further spaced dyads to determine whether these represent actual contacts or proximity. This is relevant as the macrophages are <15-30um meaning many of those are as close as the cells within dyads which changes the interpretation of what these neigbourhoods may look like.

Response: Distances reported are indeed measured from the centroid of cells. To further substantiate our findings using orthogonal methodology based on actual contact, we used *imcRtools* (developed by the Bodenmiller group) to perform pairwise interaction analyses (PMID: 28783155). Using spatial interaction graphs constructed within the *steinbock* framework via Delaunay triangulation, we determined nearest neighbours for each cell in the IMC dataset. From these graphs, we computed the average interaction count for each cell phenotype pair. We observed a strong increase (3-4 fold) in CD8$^+$ T cells localizing next to DCs in recurrence patients (compared to disease-free; see **Supplementary Fig.4f**). Conversely, 2-fold more HLA-DR^{hi} DCs were identified as direct neighbours of non-naive CD8$^+$ T cells in recurrence patients (**Supplementary Fig.4f**). Comparing interaction counts against an empirical null distribution derived by permuting all cell labels revealed that contacts between non-naive CD8$^+$ T cells and HLA-DR^{hi} DCs occurred more frequent than expected by random chance in R patients – but not in the DF cohort (**Supplementary Fig.4f**). Hence, this analysis supports the increased formation of CD8$^+$ T cell-DC dyads in the TDLN paracortex in patients that later developed distant tumor metastases. We have also added representative example of such dyads in the revised manuscript (**Fig.1j**) and expanded the Methods section accordingly (**page 18-19**).

6. Could the authors apply some clustering approaches when looking at dyads to determine whether there is increased clustering of DC and CD8 T cells or whether there is an increase simply due to increased numbers of DC within these neighbourhoods? This also applies to the % of macrophages near a dyad. Is this simply that with the increased number of dyads most of the ROI lies within 30um of a dyad? It would, again, be good to see some representative images of this to clarify. It would also be useful to have more clarity about the y-axis as it appears to say % of cells in a dyad with a DC, if this refers to the percentage of CD8 T cells contacting DC then it seems unusual that there are many cells in contact with DC given the usual rarity of DC within a node.

Response: As discussed in our response to comment #5 above, nearest-neighbour analysis combined with random permutation of cell labels indicates that the increased DC numbers in recurrence patients is also accompanied by statistically significant ($P < 0.0001$) interaction frequencies with (non-naive) CD8$^+$ T cells (**Supplementary Fig.4f**). Unfortunately, we were unable to apply this methodology to dyad-macrophage interactions as the software used did not allow us to permute labels consisting of multiple cell types (e.g. dyads). Instead, we conducted a pairwise interaction analysis at the ROI level across CD8$^+$ T cells, DCs and macrophages. For this, every pairwise interaction is tested for a statistically significant interaction or avoidance pattern in every ROI. We observed increased relative interaction frequencies between CD8$^+$ T cells, DCs and macrophages in patients with recurrence (**Fig.1h**). Representative examples of CD8$^+$ T cell-DC dyads with macrophages in close proximity have been included the revised manuscript (**Fig.1j**). Regarding the y-axis of **Fig.1i**: the reviewer Is correct, it refers to the % of CD8$^+$ T cells engaged in a dyad (i.e. within 15 μm from a DC as measured from the cell centroids); we have modified the figure legend to clarify this. While DCs indeed are relatively rare, we see single DCs often engaging with multiple CD8$^+$ T cells (e.g. **Fig.1j**). Combined with the substantial increase in DC numbers in recurrence patients (**Supplementary Fig.3a**) and their enhanced propensity to interact (**Supplementary Fig.4f**), relatively high proportions of CD8$^+$ T cells may engage in crosstalk with DCs.

7. The mouse experiments could do with some additional controls. The Pla2g2d-/- mice have slower growth but it is unclear if this is due to impacts of development etc. in these mice, given that the Pla2g2d is mainly expressed by myeloid cells would it be possible to repeat this in bone marrow chimeras so that the Pla2g2d-/- or wt bone marrow is placed into wildtype mice which will be less influenced by this and could be more robust? This section would also be improved by using a more relevant animal model for the study. Given the focus on metastatic recurrence it would be better to use the B16F10 resection model where the primary tumor is removed and metastasis is monitored. This could also be done using HCMEL or other metastatic lines. This would make this more relevant to the rest of the paper rather than switching to primary tumor growth.

Response: The reviewer raises valid points here. Previous work has shown that *Pla2gd2*$^{-/-}$ mice show normal gross development, survival and behavior (PMID:23690440). Importantly, knockout animals are virtually indistinguishable from their wildtype counterparts in terms of immune cell composition in spleen, various lymph nodes and thymus when examined under normal conditions (PMID:23690440). As suggested by the reviewer, we conducted bone marrow chimera experiments, which demonstrate that the therapeutic effect of *Pla2g2d* ablation is only observed when *Pla2g2d* is deleted in the hematopoietic compartment (**Fig.4d-f**). These findings support the myeloid origin of PLA2G2D and rule out contributions from stromal cells that may express *Pla2g2d*.

The overarching goal of our study was to identify new targetable pathways of immune suppression in the TDLN niche that contribute to impaired anti-tumor immunity. Our melanoma discovery strategy was based on the observation that patients with poor survival outcomes show activation of immune suppressive mechanisms in TDLNs, as shown by our previous work (Dammeijer *et al*. Cancer Cell 2020, van Krimpen et al. Cancer Cell 2022). Hereby, we are not perse focusing on the phenomenon of disease recurrence after surgery, but much more on the broader consequences of impaired anti-tumor immunity and how this licenses aggressive, metastatic disease. We regret not making this clearer in the original manuscript: the melanoma cohort primarily served as a discovery platform, with emerging candidate pathways subsequently investigated in a broad context of anti-tumor immunity – beyond only melanoma or cancer recurrence after surgery. In our revised manuscript, we have now clarified this in the Discussion section (**page 5-6**).

Nevertheless, we agree with the reviewer that our initial manuscript lacked a mouse model for metastatic disease. To better connect our *in vivo* findings to the human patient data, we first conducted additional imaging analyses of patient TDLNs. These show a similar induction of myeloid cell-derived PLA2G2D levels in both stage 2 and stage 3 patients, as well as substantially increased interactions between PLA2G2D^{+} myeloid cells and (PD-1^{+}) CD8^{+} T cells – again independently from the presence of TDLN metastasis (**Fig.2g**). In addition, we repeated our *in vivo* experiments with antibody-mediated PLA2G2D targeting by employing the B16F0-derived LN6-987AL model of LN-metastatic melanoma (PMID:35525247). In this model, melanoma cells metastasize to the TDLN which aligns well with the stage III melanoma TDLN setting used in our initial discovery efforts. Also in this context, including experiments conducted with *PLA2G2D*-humanized mice and human-specific PLA2G2D blocking antibodies, we show anti-PLA2G2D efficacy in the LN6-987AL melanoma model (**Fig.4-5**, **Supplementary Fig.12h)**. Extensive *in vivo* immunophenotyping using flowcytometry revealed expansion of tumor-specific CD8^{+} T cells upon PLA2G2D inhibition, initially in TDLNs and subsequently in tumors (**Fig.5a-c**). Single-cell RNA-sequencing performed in this model demonstrated that *Pla2g2d* expression is confined to a LN-specific macrophage subset that was strongly expanded in TDLNs and largely absent from tumors (**Fig.4h-m, Supplementary Fig.10i**). Importantly, we observed significant transcriptional overlap between macrophages in the TDLN paracortex of stage III melanoma patients with poor outcomes and LN6-987ALmetastatic melanoma-bearing mice (**Fig.4n**), further supporting the use of this mouse model to mimic our initial patient target discovery strategy (**Fig.2**).

8. In Fig 4f, could the authors test whether PD-1 blockade is significantly adding to the growth reduction of the 47.M2 addition? It is unclear whether this test was performed and it would be useful to interpret the conclusion that the 47.M2 with PD1 delayed growth considering that the 47.M2 is borderline significant alone? This would also be helped by displaying in the supplemental the individual growth curves to give an idea of spread and different growth trajectories.

Response: To further substantiate these findings, we have repeated and expanded our original experiments to test for additive or synergistic effects when combining PLA2G2D inhibition with anti-PD-1 blockade. This included testing in additional models, including the LN6-987AL LN-metastatic melanoma model, but also using humanized *PLA2G2D*-expressing mice. In the revised manuscript, **Fig.3f-g** now shows that in PD-1 sensitive (MC38 and LN6-987AL) models, anti-PLA2G2D monotherapy significantly delays tumor growth – which is further enhanced when combined with anti-PD-1 (**Fig.5i**, **Supplementary Fig.12f-h**). Improved efficacy in the combination therapy group was accompanied by an increased presence of tumor-specific and IL-2 producing CD8^{+} T cells (**Fig.5j**). Importantly, combined PLA2G2D/PD-1 inhibition substantially reduced tumor growth in humanized *PLA2G2D*-expressing mice carrying anti-PD-1 resistant B16F10 tumors ($P < 0.0001$) – despite only minor effects observed in both monotherapy groups (**Fig.5h, Supplementary Fig.12g**). Individual tumor growth curves have now been added to the Supplementary material (**Supplementary Fig.12g-h**), and we have revised the Discussion section accordingly (**page 6**).

9. Fig 3c could be discussed more clearly as the assertion that macrophages are more inflammatory seems likely, however, there are both IFN-Mac4(22) and IFN-Mac(07) which are downregulated. What leads to this discrepancy? Is there some more nuance to the interpretation needed?

Response: We thank the reviewer for bringing this to our attention. The pan-cancer atlas we used for this analysis contains 4 interferon-associated macrophage signatures, which show substantial overlap but also sets of unique genes (PMID: 38969631) – explaining why differential enrichment can be observed. While there is clearly a set of interferon-regulated genes – particularly those linked to IFNγ – differentially expressed in macrophages from the two patient groups (**Supplementary Fig.6a-b**), we fully agree that conclusions about the role of interferon signaling in driving TDLN macrophage phenotypes should be nuanced. We have modified the text accordingly (**page 3**, **page 5**). To provide more clarity to the figure (i.e. revised **Fig.2**), we chose to reduce the number of pathways depicted. We now show selected pathways based on the strongest GSEA enrichment scores and clear links to manually curated gene sets (**Fig.2c**). To aid in this selection process and to enhance overall interpretation, we also performed unbiased biological pathway enrichment analysis (using Metascape; see new **Supplementary Fig.6d**).

10. Is there a reason for the lack of PD-1 expression within the PD-1 sorted CD8 T cells in Fig 3f? It seems unusual that sorted PD-1+ T cells would only be 20% PD1+ by this analysis.

Response: The reviewer is correct in stating that percentages shown in the original Fig.3f were rather low. When re-visiting our flowcytometry data, we noticed that PD-1^{dim} cells were not included in our PD-1^{+} gate. Including these cells in a re-analysis revealed substantially higher percentages of positive cells (e.g. from 20% to 50% PD-1+ non-naive cells; see new **Supplementary Fig.8e** and **Reviewer Fig.1b** above), which are more in line with what one would expect after 5 days of stimulation.

11. For Fig 4a it would be helpful to do an influential point analysis for the correlation looking at the extreme outlier. Given the low number of points it seems possible that this is influencing the outcome of the analysis.

Response: We agree with the reviewer: removing the outlier value strongly reduced the observed correlation strength (i.e. from R = 0.8 to R = 0.32). Hence, we decided to remove this plot from the manuscript.

12. The description of the methods is vague. The methods, especially concerning image preprocessing e.g. denoising, could do with expansion.

Response: We agree with the reviewer and have therefore invested considerable effort into expanding our Methods section (**page 17-19**), paying special emphasis on the image processing section (please also see our responses to comments #1-6 above).

13. For Supplementary Table 1 it would be good to clarify what the values for the age correspond to i.e. mean(range) or mean(some other interval), since the range wouldn't cover the subgroups (similar for the Breslow Mean).

Response: We have revised **Supplementary Table 1** accordingly.

14. In Figure 1d, the heatmap label under the neighbours column is cut.

Response: We thank the reviewer for catching this formatting error, which has been amended in the revised manuscript (**Fig.1c**).

15. In Supplementary Fig 2 d, there are duplicated labels for patients 21 and 22.

Response: We apologize for this error: these should have been labeled as 23 and 24. We have modified the figure accordingly (**Supplementary Fig.2c**).

16. Individual datapoints should be shown in 2e, 2i, 2h, 2j and 4a (it appears from 4b that this should be

feasible given the n, however, it may be that this makes the graphs uninterpretable but this is a suggestion to aid in seeing raw data more clearly).

Response: We agree with the reviewer and added the individual datapoints to the corresponding graphs in the revised manuscript (i.e. **Fig.1f**, **Fig.1i**, **Fig.2d**).

17. Some statistical analysis of Sup Figure 8d would be welcome as while it does seem to show that there is reduced proliferation of PD1hi cells there are several individuals where the PD1int seem to show similarly low proliferation. As such some unbiased statistical analysis would help demonstrate the point that these PD1hi persist less well.

Response: We have added the requested statistical analyses.

Comments on data integrated from Umetani et al.

2. There are consistent issues with the statistics used and the interpretation thereof.

Response: We have re-visiting the statistical analyses used in all data integrated from the Umetani *et al.* manuscript. In the revised merged paper, we have added information on which statistical test was used (including checks for normality). Moreover, (whenever possible in terms of maintaining readability) we have added individual datapoints to the graphs (e.g. **Fig.1f**, **Fig.1h**, **Fig.2d**).

4. Several experiments seem to be lacking specific controls which could also strengthen the claims made within the manuscript. Specific examples include: Fig 2A has no 0 drug control (which is present in Fig 2B); Fig 1E doesn't include the PLA2G2D-H74Q-Fc included despite this being critical for the conclusions about non-enzymatic activity; Fig 6 seems to use different antibody clones for each assay without cross comparison between functions; the use of the PLA2G2A treatment is a nice control in Fig 3C and it is unclear why this was not used more broadly throughout; there isn't a characterisation of the baseline differences in PLA2G2D-/- mice which may have differences in the lymph nodes and spleen given the baseline expression – this would be needed to determine whether these effects are due to cells in the tumor microenvironment and not, say, due to widespread issues in lymphoid tissue organisation.

Response: We thank the reviewer for pointing these out. We have now added the accidentally omitted 0 drug control (new **Fig.3c**). Regarding the inclusion of a PLA2G2D-H74Q-Fc group to Fig.1e (the current **Fig.3b**): we have until now only produced the enzymatic mutant at a scale sufficient for *in vitro* assays, and convinced by these results (i.e. catalytically inactive PLA2G2D still shows potent T cell suppression; **Fig.3c**) we decided not to prioritize these experiments. Cross-comparisons of the different antibody clones are based on our PBMC-based T cell functional assay (**Fig.3e**) – the primary assay we employed to screen for function-blocking antibodies. It is important to note that all antibodies, and particularly the lead anti-human 44.H1 and mouse-reactive 47.M2 antibodies, were extensive tested for specificity of our anti-PLA2G2D antibodies to PLA2G2D in cross-reactivity ELISA assays using all the different family isoforms of the sPLA2 family (now shown in **Supplementary Fig.10f-g**). We also tested the relative ability of our anti-PLA2G2D antibodies to impair the enzymatic activities of these sPLA2 family members (now in **Supplementary Fig.10h**; representative data for 44.H1 is shown). These data demonstrate that our antibodies only bind and inhibit the enzymatic activity of PLA2G2D – and none of its other.

Concerning the use of PLA2G2A as a control: we did test the effect of PLA2G2A protein on T cell function in the same *in vitro* functional assay, demonstrating that PLA2G2A does not impair T cell proliferation as seen with PLA2G2D (these data are now included in **Supplementary Fig.10a**). Finally (as discussed above), previous work has thoroughly characterized baseline differences between wildtype and *Pla2gd2*$^{-/-}$ mice (PMID:23690440). The authors showed that *Pla2gd2*$^{-/-}$ mice exhibit normal gross development, survival and behavior. Importantly, knockout animals are virtually indistinguishable from their wildtype counterparts in terms of immune cell composition in spleen, various lymph nodes and thymus when examined under normal conditions (PMID:23690440).

7. While it is true that PLA2G2D is highly expressed by myeloid cells, the staining which is quite broad raises the possibility of other cellular sources. Could the authors address this by showing lack of expression in other

cells in the tumor microenvironment or by making bone marrow chimeras to show that the source of PLA2G2D is from the haematopoietic cells at least?

Response: Accurate identification of the source of PLA2G2D is indeed of key importance. As suggested by the reviewer, we conducted bone marrow chimera experiments, which demonstrate that the therapeutic effect of *Pla2g2d* ablation is only observed when *Pla2g2d* is deleted in the hematopoietic compartment (**Fig.4d-f**) – also ruling out contributions from stromal cells that may express *Pla2g2d*. Moreover, newly generated single-cell transcriptome analyses of TDLNs and primary tumors from mouse models of (LN-metastatic) melanoma revealed that *Pla2g2d* expression was confined to a specific macrophage population in TDLNs that was largely absent from tumor samples (**Fig.4h-m**) – immediately preceding the start of the anti-PLA2G2D therapeutic effects (**Fig.4b, 4g**). These findings support our conclusion that recurrence-associated TDLN myeloid cells produce PLA2G2D to regulate anti-tumor immunity.

Minor concerns:

1. In figure 4B and 4C the graphs for the H74Q data appears to have had the y axis set in such a way that ne of the higher data points is not shown (in the Fc control group there are only 2 data points and there should be 1 above the error bar)

Response: We apologize for this oversight and have corrected the graphs in the revised manuscript (**Supplementary Fig.12**).

Reviewer #3

Krimpen et al claim that spatial analysis of tumor-draining lymph nodes from melanoma patients with and without early recurrence identified PLA2G2D as novel cancer immunotherapy target. In the first three figures they highlight the cell type annotations, their composition differences, cell interactions and transcriptional differences associated with recurrence and disease free status. They identify an immune-suppressive myeloid hub in the TDLN i.e., immunoregulatory DCs, macrophages and pre-exhausted CD8 T cells that is associated with disease recurrence. Further they show that PLA2G2D expressed by myeloid cells is likely to play an immunosuppressive role and claim that PLA2G2D loss-of-function or therapeutic inhibition markedly improved survival in tumor-bearing mice and increased the efficacy of anti-PD-1 immunotherapy.

Overall, this manuscript is largely descriptive with many hypothesis and requires extensive functional studies to support the central conclusions. My major concerns are outlined below:

Response: We appreciate the constructive and elaborate feedback from the reviewer on our manuscript. We have integrated key data from the Umetani *et al*. companion paper and conducted extensive new experiments – including more functional studies. Together, we believe our revised study now provides compelling evidence for PLA2G2D as a targetable immune checkpoint for cancer immunotherapy, and hope that the reviewer agrees.

Major concerns.

1. Lacks mechanistic and functional data: The major goal of their spatial analysis of tumor-draining lymph nodes (TDLN) is to understand the mechanisms that lead to recurrence of melanoma after surgical resection. Although spatial analysis of the TDNL has yielded interesting associations with recurrence, including a potential role of PLA2G2D, these are simply hypothesis generating experiments. These hypotheses need to be tested in relevant models to determine causal mechanisms underlying recurrence. Importantly, the authors fail to show that PLA2G2D expression in TDLN plays an important role in disease recurrence? The same holds true for the role of the immune-suppressive myeloid hub in the TDLN i.e., immunoregulatory DCs, macrophages and pre-exhausted CD8 T cells in disease recurrence. The functional experiments using Pla2g2d-deficienct mice or anti-PLA2G2D blocking antibody neither highlights a role of PLA2G2D in recurrence or assesses its function in modulating immune responses in the TDLNs. The sister paper (Umetani et al) also does not explore the role of PLA2G2D in TDLN or recurrence models. The authors should consider experiments in relevant tumor

recurrence models to assess the role of PLA2G2D in TDLNs. In addition, selective depletion of specific immune subsets in these models is likely to clarify how recurrence is mediated by the interactions between immunoregulatory DCs, macrophages and pre-exhausted CD8 T cells in the TDNL.

Response: The reviewer raises several relevant points here, which we address individually below:

1. The overarching goal of our study was to identify new targetable pathways of immune suppression in the TDLN niche that contribute to impaired anti-tumor immunity. Our melanoma discovery strategy was based on the observation that patients with poor survival outcomes show activation of immune suppressive mechanisms in TDLNs, as shown by our previous work (Dammeijer *et al*. Cancer Cell 2020, van Krimpen et al. Cancer Cell 2022). Hereby, we are not perse focusing on the phenomenon of disease recurrence after surgery, but much more on the broader consequences of impaired anti-tumor immunity and how this licenses aggressive, metastatic disease. We regret not making this clearer in the original manuscript: the melanoma cohort primarily served as a discovery platform, with emerging candidate pathways subsequently investigated in a broad context of anti-tumor immunity – beyond only melanoma or cancer recurrence after surgery. In our revised manuscript, we have now clarified this in the Discussion section (**page 5-6**).

2. Nevertheless, we agree with the reviewer that our initial manuscript lacked a mouse model for metastatic disease. To better connect our *in vivo* findings to the human patient data, we first conducted additional imaging analyses of patient TDLNs. These show a similar induction of myeloid cell-derived PLA2G2D levels in both stage 2 and stage 3 patients, as well as substantially increased interactions between PLA2G2D$^+$ myeloid cells and (PD-1$^+$) CD8$^+$ T cells – again independently from the presence of TDLN metastasis (**Fig.2g**). In addition, we repeated our *in vivo* experiments with antibody-mediated PLA2G2D targeting by employing the B16F0-derived LN6-987AL model of LN-metastatic melanoma (PMID:35525247). In this model, melanoma cells metastasize to the TDLN which aligns well with the stage III melanoma TDLN setting used in our initial discovery efforts. Also in this context, including experiments conducted with *PLA2G2D*-humanized mice and human-specific PLA2G2D blocking antibodies, we show anti-PLA2G2D efficacy in the LN6-987AL melanoma model (**Fig.4-5**, **Supplementary Fig.12h)**. Extensive *in vivo* immunophenotyping using flowcytometry revealed expansion of tumor-specific CD8$^+$ T cells upon PLA2G2D inhibition, initially in TDLNs and subsequently in tumors (**Fig.5a-c**). Single-cell RNA-sequencing performed in this model demonstrated that *Pla2g2d* expression is confined to a LN-specific macrophage subset that was strongly expanded in TDLNs and absent from tumors (**Fig.4h-m, Supplementary Fig.10i**). Importantly, we observed significant transcriptional overlap between macrophages in the TDLN paracortex of stage III melanoma patients with poor outcomes and LN6-987ALmetastatic melanoma-bearing mice (**Fig.4n**), further supporting the use of this mouse model to mimic our initial patient target discovery strategy (**Fig.2**).

3. Following the reviewer's request, we conducted experiments to determine the cellular mediators of PLA2G2D-mediated control of anti-tumor immunity. Bone marrow chimera experiments using PLA2G2D-deficient mice revealed a hematopoietic origin of anti-PLA2G2D's therapeutic effect, excluding stromal contributions (**Fig.4d-f**). Subsequent antibody-based [redacted] CD8$^+$ T cells [redacted] *in vivo* revealed that the anti-PLA2G2D therapeutic efficacy is strictly dependent on [redacted] CD8$^+$ T cell [redacted] (**Fig.4g**). As discussed above, new single cell sequencing experiments identified LN-specific macrophages as the source of PLA2G2D *in vivo* (**Fig.4h-m**). These macrophages expressed low levels of *CSF1R*, encoding the CSF-1 receptor critical for most macrophages and monocytes. [redacted] Altogether, we believe that our revised manuscript provides strong support for PLA2G2D as a myeloid TDLN-centered immune checkpoint that can be broadly exploited for cancer immunotherapy – including but not restricted to metastatic disease.

2. Lack of clarity between disease recurrence and anti-PD1 resistance. In the method section on patient inclusion, the authors state that no patients received neo-adjuvant treatment. Also there is no mention of anti-PD1 therapy, although majority received adjuvant chemotherapy. Thus, the observations in their patients are limited to disease recurrence, presumably in the setting where immunotherapy is not given. If this is not the case the authors should provide details of immunotherapy post-surgery. It is puzzling as to why the authors have taken their results on association of PLA2G2D with disease recurrence to testing whether "PLA2G2D

mediated resistance to immune checkpoint inhibition". Nevertheless, the results of their experiments does not clearly show that combined treatment (anti-PD1 or anti-PLA2GD2 antibody) when compared to monotherapy results in significant difference in tumor control; comparisons are made with isotype control (Fig. 4f). Furthermore, there appears to be no significant difference between anti-PD1 versus anti-PLA2G2D antibody treatment (Fig. 4f). In conclusion, the authors have not clearly demonstrated a mechanistic link between PLA2G2D and anti-PD1 resistance.

Response: We apologize for any confusion created by a lack of patient information. None of the patients in our initial spatial proteogenomics cohort and subsequent validation cohorts received neo-adjuvant treatment or any form of immunotherapy. For the melanoma cohorts, not even adjuvant immunotherapy was given as samples used predated the widespread adoption of immunotherapy in resectable melanoma. We have modified the main text and methods section to accurately reflect this (**page 2**, **page 16-17**).

To strengthen the link between PLA2G2D-mediated immunosuppression and anti-PD-1 therapy as well as to substantiate our hypothesis of improved tumor control in a combination therapy setting, we conducted several new experiments. Antibody-based [redacted] of $CD8^+$ T cells [redacted] *in vivo* revealed that the anti-PLA2G2D therapeutic efficacy is strictly dependent on [redacted] $CD8^+$ T cell [redacted] (**Fig.4g**). Moreover, immuno-monitoring and single cell RNA-Seq experiments revealed that PLA2G2D inhibition provides an early boost to tumor-specific T cell activation and suppression of tumor growth (**Fig.4-5**). Nevertheless, tumor growth was often not fully controlled, and whereas PD-1 expression was relatively low on both total and tumor-specific T cells in TDLN and peripheral blood, PD-1 levels increased throughout time – with tumor-specific $CD8^+$ T cells being already $PD\text{-}1^+$ at the earliest timepoints in the tumor (**Fig.5e**, **Supplementary Fig.11a**). Together, these observations provide a solid rationale for combining PLA2G2D and PD-1 inhibition. We tested this concept in additional models, including the LN6-987AL LN-metastatic melanoma model, but also using humanized *PLA2G2D*-expressing mice. In the revised manuscript, **Fig.5f-i** and **Supplementary Fig.12f-h** now shows that in anti-PD-1-sensitive models (MC38 and LN6-987AL), anti-PLA2G2D monotherapy significantly delays tumor growth – which is further enhanced when combined with anti-PD-1. Improved efficacy in the combination therapy group was accompanied by an increased presence of tumor-specific $CD8^+$ T cells and local IL-2 production (**Fig.5j**). Importantly, combined PLA2G2D/PD-1 inhibition strongly reduced tumor growth and boosted tumor-specific T cell responses in humanized *PLA2G2D*-expressing mice carrying anti-PD-1 resistant B16F10 tumors ($P < 0.05$) – despite only minor effects observed in both monotherapy groups (**Fig.5h**, **Supplementary Fig.12g**). These findings showcase the potential added benefit of combined PLA2G2D/PD-1 blockade as an immunotherapy regime, with potential to revitalize anti-tumor immunity in cancers that poorly or non-durably respond to anti-PD-1 monotherapy. To what extend PLA2G2D is involved in acquired anti-PD-1 resistance, as suggested by the increased *PLA2G2D* expression levels in tumors after PD-(L)1 blockade (**Supplementary Fig.11b**; data from Memom *et al*. PMID:38215748), remains to be further investigated in subsequent studies. We have modified the Discussion section based on these findings (**page 6**).

3. Failure to cross-compare findings in the tumor. If the authors want to highlight the importance of TDLN when compared to tumor per se, it is important to compare their finding in the tumor. Are there features in the tumor that predict recurrence? Are these feature absent in the TDLN or what is the correlation? Are mechanisms that are associated with recurrence different in the tumor and TDLN? I understand that TDLN are poorly studied, and that their analysis yielded interesting insights. Would they have captured these findings by analyzing the tumor by single-cell analysis or spatial analysis? In summary, the rationale to study TDLN without analysis of tumor is not clear.

Response: We agree that relating our TDLN findings to the primary tumor is important. To this end, we first conducted imaging analyses of TDLNs, TDLN-metastases and primary tumors from an independent patient (NSCLC) cohort. This revealed a substantial and similar induction of PLA2G2D levels in DCs and macrophages from both stage 2 and stage 3 patients (**Fig.2g**). $PLA2G2D^+$ myeloid cell abundance was much higher in TDLNs than in primary tumors, irrespective of the presence of a TDLN metastasis – the latter showing the lowest levels of $PLA2G2D^+$ macrophages and DCs (**Fig.2g**). Moreover, co-localization analysis revealed substantially increased interactions between $PLA2G2D^+$ myeloid cells and ($PD\text{-}1^+$) $CD8^+$ T cells in TDLNs as compared to the (primary) tumor, again independently from the presence of a TDLN metastasis (**Fig.2h**). Finally, newly generated single-cell transcriptome analyses of TDLNs and primary tumors from mouse models of (metastatic) melanoma revealed that *Pla2g2d* expression was confined to a specific macrophage population in (TD)LNs that was largely

absent from tumor samples (**Fig.4h-m**) – coinciding with the expansion of tumor-specific CD8+ T cells in TDLNs (**Fig.5a-c**) and the start of the anti-PLA2G2D therapeutic effects (**Fig.4b**, **Fig.4g**). Together, these results – while they do not formally exclude an effect of PLA2G2D in the tumor microenvironment – support the concept of a TDLN-centered PLA2G2D immune checkpoint. We now discuss these points in the Discussion (**page 5-6**).

4. Interpretation of gene expression findings in immune cells is incomplete.
(i) T cells in recurrence patients are called as pre-exhausted based on markers. However, based on enrichment of gene signatures Figure 3C (right panel) it is clear that tissue-resident memory (TRM) (ZNF683+ (Hobit)+) is the most enriched in T cells from recurrence patients. Given the established importance of TRM cells in anti-tumor immunity and responses to anti-PD1 therapy, it is a glaring omission that the authors have failed to assess this population. Recent work from David Masopust's group has shown that TRM cells can recirculate between tissues and draining lymph nodes. TRM cells and exhausted cells share several marker genes including PD1. If the cells increased in recurrence are mainly TRM? Then why are these cells present in the lymph node and interacting with macrophages and DCs in the TDLN? Are they dealing with silent metastasis? Are they tumor-specific or just bystander cells? Many open question are unanswered.

Response: The reviewer is correct in stating that a TRM gene signature is strongly associated with the transcriptional changes we observe in paracortex TDLN CD8+ T cells from recurrence patients, although it should be noted that effector memory (TEM) and exhausted (TEX) T cell signatures show almost identical enrichment strength (**Supplementary Fig.8a**). While we fully agree that TRM cells are important for anti-tumor immunity, we want to stress that TEX cells in tumors often engage a residency program that relies on the same transcriptional regulators also used by TRM cells. As a result, TRM and TEX cells are being conflated in the literature (discussed in PMID:41461987). This is exemplified by another Masopust lab paper (PMID:37267383), showing that the TIL-derived TRM signature we used (since similar TDLN-derived signatures are lacking) is rather TRM-aspecific, prompting the authors to conclude that a TRM-like label would be more appropriate (which we have now added). Hence, it remains unclear whether the TRM annotation from the TIL-atlas we used is specific for bona fide TRM cells, which likely explains the strong enrichment for both TRM and TEX signatures in our analyses. Conveniently, the MacKay lab very recently published curated gene signatures specifically developed to separate human TRM/TEX phenotypes (PMID:41461987). Using these new subset-specific signatures to further annotate our own TDLN CD8+ T cell signature revealed a strong enrichment for genes upregulated in TEX as compared to TRM cells – and no significant enrichment of TRM-specific signatures (**Supplementary Fig.8a; right panel**). These data support the notion that paracortex CD8+ T cells from recurrence patients show phenotypic similarities with exhausted T cells in tumors.

(ii) The macrophage population linked to recurrence is labelled as immunosuppressive? What is the evidence to support this? Several single-cell studies of myeloid populations in the tumor have described the presence of CXCL9 expression macrophages (i.e., interferon response signatures). These macrophages with interferon response signatures are linked to good anti-tumor outcomes as in PMID: 37535729. Thus, it is important to not overstate the functional properties based on a few molecules. Gene expression studies only generate hypotheses, these need to be rigorously tested in functional models before making firm conclusions about mechanisms.

Response: It was not our intention to draw too strong conclusions about macrophage phenotypes solely based on transcriptional differences, and we have added nuance to such qualifications in the revised manuscript (**page 3**; 'immunosuppresive' is now primarily used as a qualification of specific molecules expressed by macrophages). Indeed, TDLN macrophages associated with poor outcomes show clear signs of an activated phenotype and interferon exposure, which for tumor-associated macrophages has been linked to both favorable (as mentioned by the reviewer) but also poor outcomes (e.g. PMID: 38602878, PMID:27912061). However, we were particularly drawn to the striking upregulation of known immunosuppressive molecules by these TDLN macrophages, including IDO1, IL4I1, PD-L1 (CD247) and of course PLA2G2D (both on RNA and protein level: **Fig.2b-d**, **Supplementary Fig.4e**). We have now performed additional experiments that support this conclusion. Our combination of bone marrow chimeras, genetic/antibody-mediated depletion and single cell RNA-sequencing experiments have now demonstrated that lymph node macrophages are the *in vivo* source of PLA2G2D (**Fig.4-5**, **Supplementary Fig.10-11**) – a population of cells that showed strong transcriptional resemblance to the TDLN paracortex macrophages in melanoma patients with poor outcomes (**Fig.4n**). Together with our new evidence

establishing PLA2G2D as an immune checkpoint that restrains anti-tumor immunity *in vivo* (**Fig.3-5**), these new data support our conclusion that PLA2G2D^{+} TDLN macrophages can have an immunosuppressive phenotype linked to the development of metastatic disease.

5. Tumor-specific T cell analysis is lacking. A major downside of the spatial analysis as opposed to single-cell transcriptomic and TCR-seq is the ability to track potential tumor-specific T cells and test their TCR specificities. However, expression of certain marker genes like CD39 have been linked to tumor-antigen-specific T cells. Do the authors find CD39 expression in CD8 T cells? if so these may represent tumor-specific T cells and likely to be functional important. In the absence of information on the tumor-specificity of T cells in the TDLN, it is difficult to draw mechanistic insights as these cells may represent bystander cells and not likely to play roles in anti-tumor immunity.

Response: We agree that insights into how PLA2G2D regulates tumor-specific CD8^{+} T cell responses would be very insightful. To address this point, we used an H-2Kb Tyrosinase-related protein 2 (TRP2) tetramer in our flowcytometry panels to allow for the identification of T cells targeting the endogenous TRP2 neo-antigen on B16-derived melanoma cells. We preferred this over CD39 quantification, as CD39 has been primarily validated to enrich for tumor-specific T cells in *tumors*, while its predictive power in the TDLN has not yet been established (PMID:36574773, PMID:30006565). We found that anti-PLA2G2D therapy induced TRP2-specific CD8^{+} T cell expansion in TDLNs, followed by increased levels in the circulation and subsequently the tumor microenvironment (**Fig.5a-c**). These findings support the concept that myeloid PLA2G2D produced in the TDLN controls the early formation of tumor-specific T cell responses.

6. Annotations of immune cell types is incomplete. In figure 1, the annotations of many immune cell type is not complete. Labeling DCs as DRhigh and low, macrophages as CD163.1, CD163.2, CD8 as act1,act2,act3 is not adequate, there are several large-scale single-cell datasets, especially those from Miriam Merad, that have detailed accurate classifications of myeloid and DC subsets across many tumor types.

Response: The reviewer is correct in stating that in our initial IMC phenotyping efforts needed to be improved. As detailed in our response above to comment #3 by reviewer 2, we have revisited our Phenograph-based annotations (**Fig.1b-c**, **Supplementary Fig.1-2**). With only 40 markers in our IMC panel to characterize a large diversity of immune and stromal cells, we decided to focus on phenotyping accuracy and to refrain from subsetting key cell types beyond core cell states (i.e. naive/non-naive for T lymphocytes, CD163^{+}/CD169^{+} for macrophages & HLA-DR^{hi}/HLA-DR^{lo} for DCs). In this instance, gene signatures from large single cell RNA-Seq datasets would offer no additional benefit; our IMC panel is simply not large enough to superimpose complex gene expression patterns onto. Instead, we used the approach suggested by the reviewer to accurately annotate our myeloid cell clusters in the newly generated single-cell transcriptome analyses of TDLNs and primary tumors from mouse models of (metastatic) melanoma (**Fig.4**). Previously reported gene signatures of DCs and macrophage populations published by the Merad and Akkari groups (PMID:22797772 & 39137777) were instrumental in distinguishing myeloid subsets (**Fig.4h-k**), providing a reliable biological framework in which we can position the PLA2G2D^{+} lymph node macrophage subset we identified here.

7. Sample size. Another weakness is the relatively small sample size 5-6 patients per group. Inter-donor heterogeneity is a problem. Generally hypothesis seeking studies need to have a larger sample size and donor specific data should be shown throughout as opposed to aggregate data/ group for gene expression comparisons etc.

Response: While our initial spatial proteogenomics cohort is indeed relatively small, it consists of very carefully selected and well-stratified patient groups to minimize inter-donor heterogeneity and maximize potential for relevant discoveries. Importantly, we deliberately focused on pursuing a top hit (i.e. PLA2G2D was the most significantly differentially expressed gene in macrophages in our GeoMx DSP analysis; P = 7.61e-06), which we subsequently validated in multiple independent cohorts and using orthogonal assays (**Fig.2**, **Fig.4**). Although we agree with the reviewer that larger sample sizes may in principle be even better for hypothesis generation purposes, our combined experiments demonstrate that high-confidence hits from our initial discovery cohort (e.g. PLA2G2D) replicated well in validation samples and showed strong translational relevance in a set of

comprehensive follow-up experiments. In the revised manuscript, we have paid extra attention to include measurements of individual patients/donors (e.g. **Fig.1f**, **Fig.1i**, **Fig.2d**).

Overall, this study is largely descriptive, preliminary and requires more work.

Response: We hope the reviewer appreciates the substantial number of new experiments that we have added to the study to address the concerns raised. Altogether, we feel that our expanded and combined study provides compelling evidence for PLA2G2D as a TDLN-centered myeloid immune checkpoint that can be targeted for cancer immunotherapy.

Comments on data integrated from Umetani et al.

2. Inconsistency in the tumor models utilized and results. In Figure 1B,D,E, the authors utilized MC38 and E.G7-OVA model to show that Pla2g2d-/- mice display better control of tumor growth. However, in experiments using the anti-mouse PLA2GD2 antibodies (48.M1, 47.M2, 48.M3) they switched to CT26 model. What effects did they observe in the MC38 or E.G7-OVA model with antibodies targeting murine PLA2GD2? Important controls to highlight specificity of their anti-mouse PLA2G2D antibodies are missing: (i) treatment of MC38 and E.G7-OVA tumor model in Pla2g2d-/- mice with anti- PLA2G2D antibody to show that the effect of the antibody is specific to PLA2GD2, (ii) reversal of the anti-tumor effects of anti-PLA2G2D antibody with PLA2G2D fusion protein.

Response: We apologize for any confusion regarding the antibodies we generated. To improve consistency in the revised manuscript, we now tested PLA2G2D inhibition using both mouse-reactive (47.M2) and human-specific (44.H1) antibodies in several B16 melanoma models, also making use of our humanized *PLA2G2D*-expressing mice. This includes the LN-metastatic B16-LN6-987AL model, to better connect the *in vivo* work to the melanoma patient cohort we analyzed using spatial proteogenomics methods (i.e. **Fig.1-2**). We observe consistent reductions in tumor growth after antibody-mediated PLA2G2D inhibition across all melanoma models tested – either as monotherapy or in combination with anti-PD-1 therapy (**Fig.4b-c**, **Fig.4g**, **Fig.5g-i**). To demonstrate specificity of our anti-PLA2G2D antibodies to PLA2G2D, we instead tested their cross-reactivity to all the different family isoforms of the sPLA2 family by ELISA (**Supplementary Fig.10f-g**). We also tested their relative abilities to impact the enzymatic activities of these sPLA2 family members (**Supplementary Fig.10h**; representative data for 44.H1 is shown). These data demonstrate that our antibodies only bind and inhibit the enzymatic activity of PLA2G2D – and none of its other family members. We have added these data to the revised manuscript (**page 4**, **Supplementary Fig.10**).

3. Studies in tumor models are superficial and do not provide mechanistic insights. Detailed unbiased analysis (preferably single-cell analysis) of immune cells in the tumor and tumor-draining lymph nodes in at least two time points will strengthen the causal mechanisms. Since the authors claim that chronic interferon signaling induces PLA2GD2 expression in myeloid cells, do they observe this induction in the mouse tumor models used? if so, what are the kinetics of induction? This is important as the effects of PLA2G2D on anti-tumor responses are likely to be observed only after its induction in the tumor and tumor-draining lymph nodes? If PLA2GD2 fusion protein is administered to Pla2g2d-/- mice bearing MC-38, as in Figure 1E, does it modulate the tumor immune responses, what aspects are perturbed and how does it compare with responses observed in wildtype mice where PLA2GD2 in induced endogenously. Further, selective depletion of specific immune subsets in Pla2g2d-/- mice bearing MC38 tumor is likely to highlight the relative contribution of key cell types driving anti-tumor control.

Response: We agree with the reviewer that obtaining more insight into the mechanisms of action underlying PLA2G2D-mediated control of anti-tumor immunity is important. Considering the combined reviewer comments, we decided to prioritize the in-depth analysis of the impact of PLA2G2D in the more therapeutically relevant setting of PLA2G2D inhibition. To this end, we conducted a new series of *in vivo* experiments, including single cell transcriptomics and longitudinal analyses of how PLA2G2D inhibition affects anti-tumor immunity in a model of LN-metastatic melanoma (B16F0-derived LN6-987AL line) using flow cytometry (new **Fig.4-5**). Anti-PLA2G2D treatment resulted in (transiently) increased levels of proliferating memory $CD8^+$ T cells in the TDLN, blood and tumor of melanoma-bearing mice – including the improved induction of tumor-specific T cell responses (**Fig.5a-**

c). Moreover, transcriptional programs linked to T cell activation were upregulated upon PLA2G2D inhibition (**Fig.4o**). Regulatory T cell proliferation was not substantially affected by PLA2G2D-blocking antibodies (**Fig.5d**). Following the reviewer's request, antibody-based [redacted] of CD8$^+$ T cells [redacted] *in vivo* revealed that the anti-PLA2G2D therapeutic efficacy is strictly dependent on DC-CD8$^+$ T cell crosstalk (**Fig.4g**). Bone marrow chimera experiments using PLA2G2D-deficient mice revealed a hematopoietic origin of PLA2G2D, excluding a stromal contribution to the observed therapeutic effect (**Fig.4d-f**). As also detailed in our responses above, single cell transcriptomics experiments identified LN-specific macrophages as the source of PLA2G2D *in vivo*, which are highly abundant in TDLNs but largely absent from tumors (**Fig.4h-m**). This subset of PLA2G2D$^+$ LN macrophages expressed low levels of *CSF1R*, encoding the CSF-1 receptor critical for most macrophages. [redacted] Importantly, TDLN-specific expression of *Pla2g2d* closely aligned to the time window (day 10-13 post tumor inoculation) when anti-PLA2G2D induces tumor-specific CD8+ T cell expansion and starts to inhibit tumor growth (**Fig.4b, Fig.4g, Fig.5a-c**). Indeed, PLA2G2D inhibited T cell proliferation and effector cytokine production *in vitro* (**Fig.3c-d**). Together with new data showing consistently higher expression of PLA2G2D mRNA and protein in TDLNs compared to (primary) tumor samples (**Fig.2**), these findings support our model of PLA2G2D as a myeloid TDLN-centered immune checkpoint that suppresses systemic anti-tumor CD8$^+$ T cell responses to allow cancer growth.

4. Mechanism of action of PLA2G2D. The cellular receptor for PLA2G2D is not known. Furthermore, the intracellular signaling and transcriptional pathways that are activated by PLA2G2D in T cells, macrophages and DCs is not defined. These studies can be done using the catalytically mutant PLA2G2D.

Response: We agree that this is an interesting direction to pursue. When prioritizing our resources, we decided to focus on several other key aspects of the PLA2G2D mode of action: 1) pinpointing the tissue-specific cell subsets that produce PLA2G2D in patients and *in vivo* (revised **Fig.2**, new **Fig.4**); 2) elucidating the impact of PLA2G2D activity on (tumor-specific) T cell responses (new **Fig.3-5**); and 3) investigating the potential of combining PLA2G2D inhibition with PD-1 blockade (new **Fig.5**). Identifying the putative PLA2G2D receptor will require a large body of additional work, which we believe extends beyond the scope of the current paper and its figure space constraints. Important to note is that PLA2G2D strongly binds heparan sulfate proteoglycans (PMID:10383420), which must be considered and will significantly complicate efforts to identify PLA2G2D receptors.

5. Novelty. The association of PLA2G2D with cancer prognosis and non-response to PD-1 therapy is not new.

Response: The reviewer is correct in stating that elevated PLA2G2D expression has been linked to cancer prognosis and response to checkpoint inhibition. However, these studies only reported associations (e.g. based on transcriptomic analyses of tumors, e.g. in PMID:34446728 showing similar *PLA2G2D* induction inn responding and non-responding patients). Our revised study adds substantial novelty by showing that PLA2G2D acts as a myeloid immune checkpoint that is activated first in the TDLN, where it suppresses tumor-specific CD8$^+$ T cell responses through spatial cellular crosstalk in an immunosuppressive cellular neighborhood. Importantly, we show for the first time that the PLA2G2D immune checkpoint can be therapeutically targeted to improve tumor control in mouse models of cancer, offering therapeutic benefit that extends beyond – and potentially acts synergistically with – PD-1 checkpoint inhibition (**Fig.5h**).

6. Minor points:
(i) Why is the no treatment condition (0ug/ml) not shown in Figure 2A (shown for 2B). No effect is observed at 0.5 ug/ml? Is this a concern? Effects only observed at higher doses?

Response: We regret omitting this condition and have added it to the revised manuscript, showing that an effect is already observed at 0.5 μl/ml (**Fig.3c**).

(vi) Figure 5 F and G – The cellular source of PLA2G2D should be assessed using multiplex IHC or other approaches in both tumor and draining lymph nodes.

Response: This is indeed important to further investigate. We have performed multiplex immunofluorescence stainings on stage 2 and 3 NSCLC patient samples to pinpoint PLA2G2D expression to $CD68^+$ macrophages and $CD11c^+$ dendritic cells (**Fig.2g**). $PLA2G2D^+$ myeloid cell abundance was much higher in TDLNs than in primary tumors, irrespective of the presence of a TDLN metastasis – the latter showing the lowest levels of $PLA2G2D^+$ macrophages and DCs (**Fig.2g**). Moreover, co-localization analysis revealed substantially increased interactions between $PLA2G2D^+$ myeloid cells and ($PD\text{-}1^+$) $CD8^+$ T cells in TDLNs as compared to the (primary) tumor, again independently from the presence of a TDLN metastasis (**Fig.2h**). Finally, single-cell transcriptome analyses of TDLNs and primary tumors from mouse models of (LN-metastatic) melanoma pinpointed *Pla2g2d* expression to a specific macrophage population in TDLNs that was largely absent from tumor samples (**Fig.4h-m**).

(vii) Discussion – many overstatements, for example – "Thus, PLA2G2D represents a new standalone immunotherapy target option, while combining anti-PLA2G2D with anti-PD-1 has the potential to enhance ICI efficacy and overcome resistance."

Response: We have revisited the discussion section of our paper to add the appropriate nuance to our conclusions (**page 5-6**).

Point-by-point response letter – 2024-10-22118C (Van Krimpen *et al.*)

General Statement

We want to thank all referees again for investing their time into (re-)reviewing our manuscript and for providing additional feedback. Please find below our detailed responses to this feedback **in blue**; we have added versions of the revised manuscript and supplementary data with tracked changes (i.e. changes highlighted in yellow).

[redacted]

Referee #1

The combination of the 2 articles greatly improved the quality of the manuscript. It established that PLA2G2D is an immunosuppressive mechanism, quite likely dampening the immune response when inflammation is increased, as previously described in elderly people. During cancer, it clearly constitutes a break to the immune response, that can be released after genetic inactivation of blocking with an Ab. Some points from the first revision were answered but there are still some points that need to be addressed.

Response: We are very pleased to read that the referee feels that our manuscript has greatly improved. Below we address the remaining points raised, which were very helpful in further improving the manuscript.

I am still not convinced by the statement that the CD8 T cells found in the TDLN are pre-exhausted. They are primed and express a panel of activation markers, including TOX that can be expressed in polyfunctional T cells in human (10.1126/sciimmunol.aba7918) or PD1, that is also expressed in a significant portion of Tem for instance. The in vitro assay just compares primed vs non primed T cells and does not inform whether these cells are pre-exhausted or not. Indeed, the transcriptomic analysis shows that the profile can be compared to the one of Tem or exhausted cells, not just to exhausted T cells. Do the authors find similar conclusions in the mouse models they used? It would be easier to study and to formally prove this point there.

Response: We agree with the reviewer that without data on clonal relationships between paracortex Tpex(-like) cells and bona fide Tex cells in the tumour, as well as functional experiments showing impaired effector functions upon chronic antigenic stimulation, one needs to be very careful when describing exhausted T cell phenotypes. Motivated by the referee's justified criticism, we decided to conduct several additional analyses, which yield an improved understanding of T cell exhaustion in our datasets and in relation to the PLA2G2D checkpoint. The results and our interpretation are detailed below and in our responses to subsequent related comments by the referee.

Reviewer Figure 1

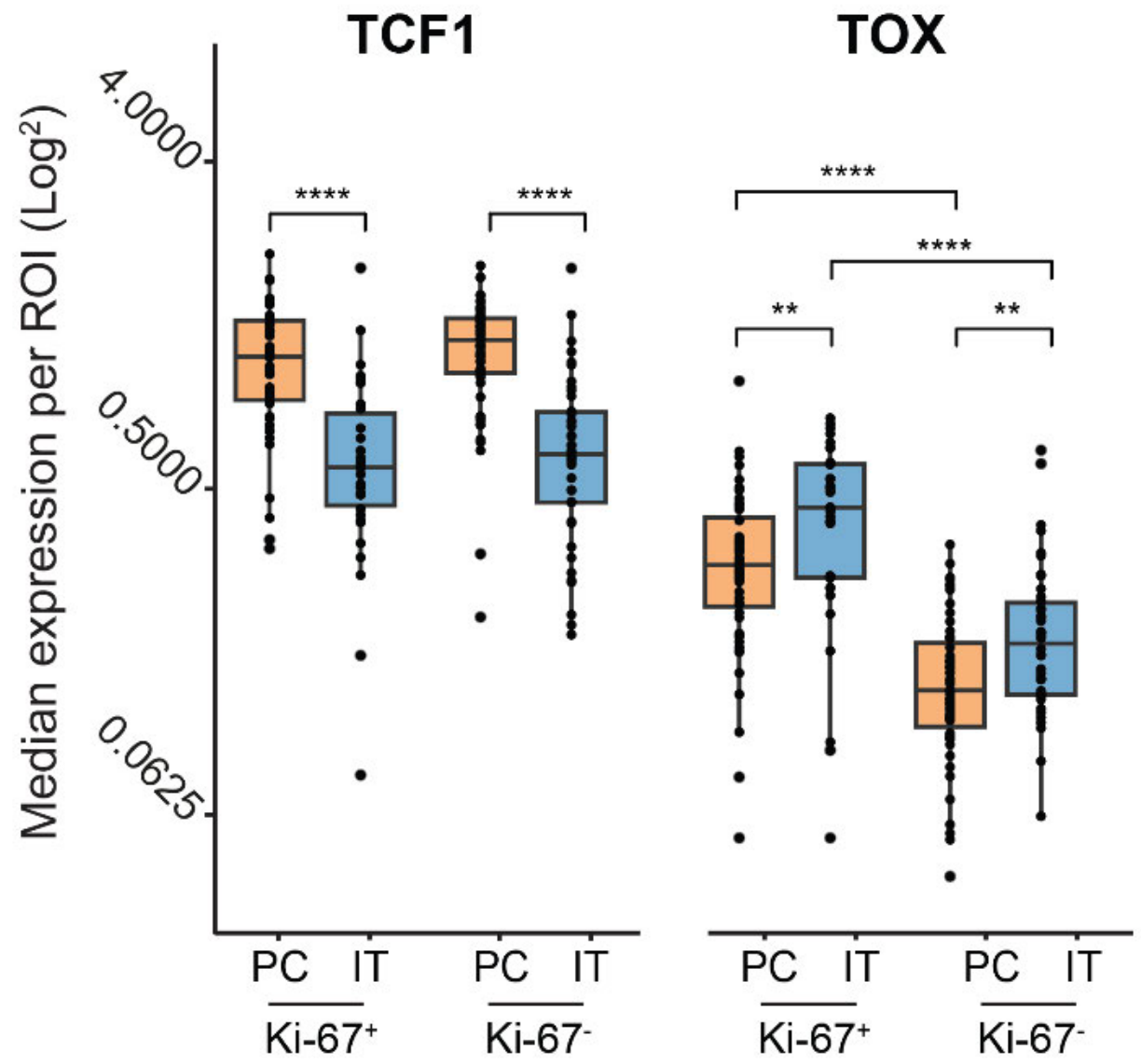

***Reviewer Fig.1**. Box plots displaying marker expression for different cell types, visualized per ROI, within the paracortex. Inverse hyperbolic sine-transformed imaging mass cytometry protein levels for TCF1 and TOX are shown in CD8$^+$ T cells, divided by Ki-67 status. Values are displayed for the paracortex (PC) and intratumoural (IT) zone of the TDLN, on a log_2 transformed y-axis (points represent individual ROIs).*

We agree with the referee that TOX and PD-1 expression are insufficient to properly characterize cells across the T cell differentiation spectrum. In the article mentioned by the reviewer (Sekine *et al.* Science Immunology 2020), the authors show that while TOX expression is not confined to Tex cells, cells co-expressing TOX, TCF-1 and inhibitory receptors are largely devoid of effector memory cells and highly enriched for chronic (EBV, CMV & HIV) rather than acute (Flu) virus-specific T cells. In both mouse (Schenkel *et al.* Immunity 2021, Connolly *et al.* Science Immunology 2021, Huang *et al.* Cell 2022) and human (Rahim *et al.* Cancer Cell 2023, Zheng *et al.* Science 2021, Pai *et al.* Cancer Cell 2023) studies, the TCF-1$^+$ TOX$^+$ CD8$^+$ T cells in the TDLN were found to be progenitors to Tex ('Tpex') cells. In all instances, TCF-1 expression in non-naive CD8$^+$ T cells (in addition to intermediate PD-1 and TOX levels) marked Tpex cells, which is in line with TCF-1 driving CD8$^+$ T-cell fate towards the Tpex-Tex lineage, rather than the (terminal) effector T cell fate (Chen *et al.* Immunity 2019). Re-examining the TOX and TCF-1 signals in our IMC dataset, we noticed – as expected – the highest TOX levels in activated (i.e. Ki-67$^+$) CD8$^+$ T cells from the tumour and lowest TOX levels in resting (i.e. Ki-67$^-$) TDLN paracortex CD8$^+$ T cells, populations enriched in Tex and naive cells, respectively (**Reviewer Fig.1**). TCF-1 levels, however, were highest in TDLN paracortex CD8$^+$ T cells, including Ki-67$^+$ cells that also showed intermediate TOX levels – akin to a Tpex phenotype. In line with the studies mentioned above, these data indeed indicate that TDLNs are enriched for TOX/TCF-1 co-expressing Tpex cells. Nevertheless, as our description of the (paracortex) Tex cell phenotypes in the manuscript is based solely on protein/RNA expression profiles, we have added extra nuance to our conclusions and included references to relevant literature (incl. Sekine *et al.* Sci Immunol 2020) to more accurately reflect on our findings (see **Summary paragraph line 36**, **page 2 line 95**, **page 3 lines 114-127** & **page 6 lines 273-278**). We have also integrated the data shown in **Reviewer Fig.1** in the manuscript (**Extended Data Fig.5g**).

The authors established in Figure 3 that PLA2G2D has a direct immunosuppressive effect on T cells, by dampening T cell proliferation and activation in vitro. Does it increase TOX expression or features associated with exhaustion?

Response: In our previous response letter, we showed additional phenotyping of CD8$^+$ T cells treated with PLA2G2D protein, revealing that in contrast to proliferation and effector cytokine production (see **Fig.3**), known activation markers (e.g. CD69, CD25) and co-inhibitory receptors (e.g. TIGIT, NKG2A, PD-1) were only modestly affected by PLA2G2D (**Reviewer Fig.2a**). Gene expression analysis revealed very low *TOX* mRNA levels in both control and PLA2G2D-treated CD8+ T cells (**Reviewer Fig.2b**). Overall, these data show that short-term exposure to PLA2G2D does not induce features associated with exhaustion *in vitro*.

Reviewer Figure 2

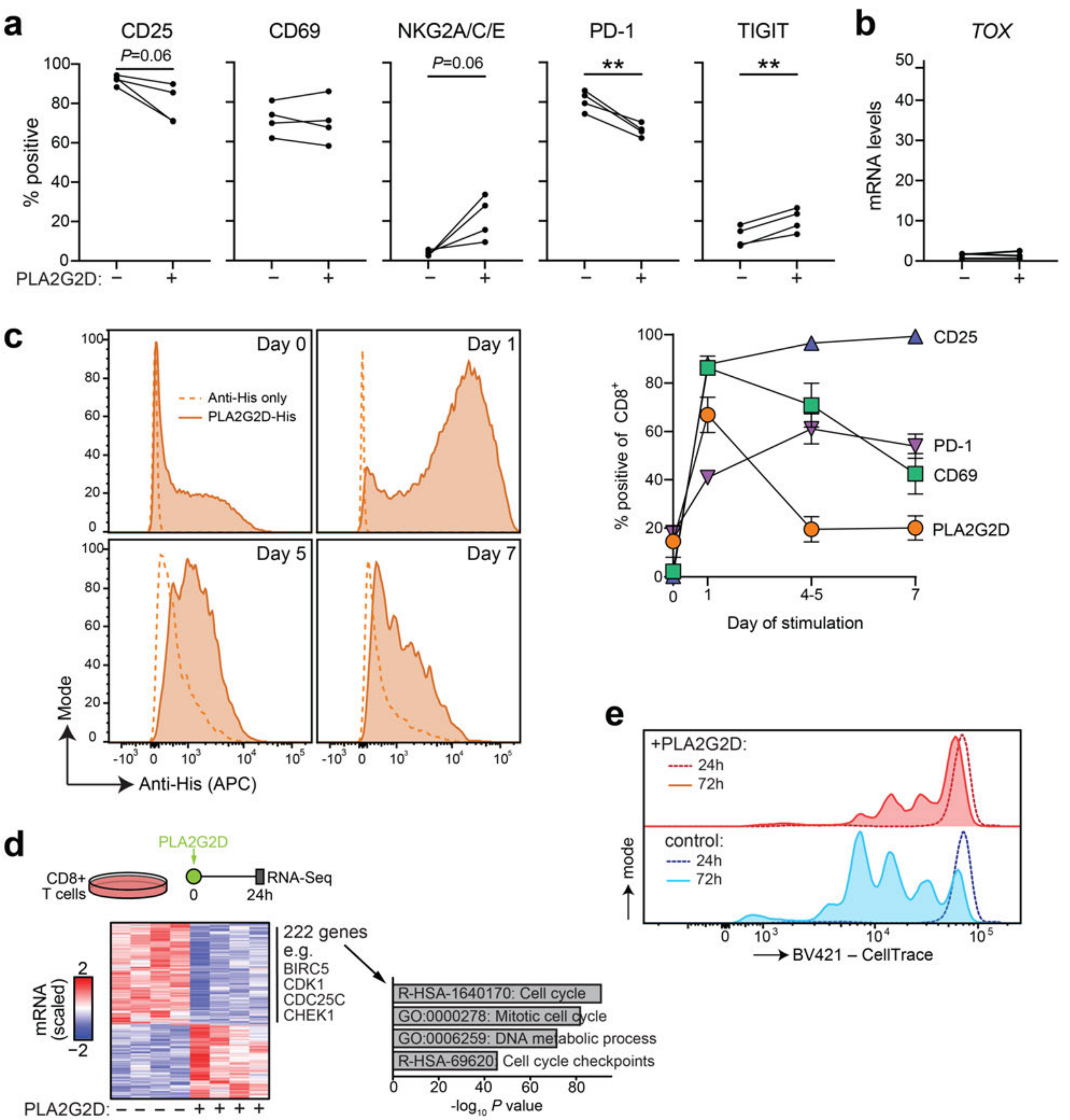

***Reviewer Fig.2**. (**a**) Flow cytometry analysis of surface marker levels on CD8⁺ T cells isolated from healthy donor PBMCs in the presence of absence of PLA2G2D protein (10 µg/ml). T cells were activated using anti-CD3/CD28 stimulation at the start of culture, analysis was conducted after 72h of incubation. (**b**) Normalized TOX mRNA levels in CD8⁺ T cells in the presence or absence of PLA2G2D (as in panel a). (**c**) Left: Flow cytometry histograms of PLA2G2D binding (visualized using an APC-coupled anti-His antibody that binds His-tagged PLA2G2D protein) on CD8⁺ T cells isolated from healthy donor PBMCs. Cells were activated using anti-CD3/CD28 stimulation. PLA2G2D binding to the T cell surface was assessed at baseline (day 0) and 1, 5 and 7 days after stimulation. Right: Line graph shows quantified percentages of CD8⁺ T cells expressing the indicated surface markers or surface-bound PLA2G2D throughout the time-course (n=2-3 donors, +/- SEM). (**d**) Healthy donor CD8⁺ T cells (n=4) were exposed for 24h to PLA2G2D protein or left untreated and analysed using RNA-Seq. Heatmap shows differentially expressed genes (absolute log_2 FC > 0.5, adj. P < 0.05) upon PLA2G2D treatment, consisting of 222 upregulated genes strongly enriched in biological pathways linked to cell cycle control (bar graph). (**e**) Proliferation dye dilution assay of CD8⁺ T cells exposed to PLA2G2D (red) or left untreated (blue). CellTrace staining was measured after 24h and 72h.*

But perhaps this is not too surprising, as short-term cultures of TCR-stimulated naive T cells are generally unable to induce an exhausted phenotype (e.g. Belk *et al.* Cancer Cell 2022). Intriguingly, when we monitored the capacity of T cells to bind PLA2G2D throughout time-course stimulation experiments (with anti-CD3/CD28 and cells cultured in the absence of PLA2G2D protein in the medium) we found a very rapid induction (i.e. within 24h) of PLA2G2D binding capacity on early activated $CD8^+$ T cells – in parallel to the upregulation of well-known early activation markers CD69 and CD25 (**Reviewer Fig.2c**). However, in contrast to CD25 and PD-1, PLA2G2D binding capacity very rapidly returned to baseline levels at later timepoints, suggesting that the PLA2G2D checkpoint specifically suppresses very early T cell activation. RNA-Sequencing of primary human $CD8^+$ T cells exposed to PLA2G2D revealed a specific and rapid (i.e. within 24h) suppression of cell cycle-related pathways (**Reviewer Fig.2d**), which resulted in strongly reduced subsequent T cell proliferation (**Reviewer Fig.2e**). This is in strong agreement with our findings showing anti-PLA2G2D to improve tumour-specific T-cell priming and proliferation *in vivo* (**Fig.5**). Altogether, our observations further support a direct effect of PLA2G2D inhibition on T cells. We have integrated part of the data shown in **Reviewer Fig.2** in the manuscript (**Fig.3e-g**, **Summary paragraph line 41**, **page 4 lines 161-166** & **page 6 lines 270-278**).

In mouse model in vivo, the authors studied the effect of blocking Ab in various tumor models, with a focus on tumor specific T cells. The scRNAseq data on T cells (Fig 4O) shows that indeed the blocking of PLA2G2D leads to higher activation, and so by higher levels of exhaustion associated genes that are also expressed transiently upon activation (entpd1, pdcd1, Bhlhe40, Runx2,…). It is not clear to me whether this analysis was performed only on TDLN or combined both TDLN and tumor CD8 T cells. A focus on TDLN would clarify whether in absence of blocking, PLA2G2D promotes "pre-exhaustion" or not. Flow cytometry study on tumor specific CD8 T cells could also be performed.

Response: Fig.4o (now **Fig.4q**) depicts the outcome of a differential gene expression analysis on the combined set of $CD8^+$ T cells from both TDLN and tumour, which was required to enhance statistical power in our relatively small scRNA-Seq dataset. We have adapted the figure legend to make this clearer. To better address the reviewer's point – also catering to the previous comments above – we used flow cytometry as suggested by the reviewer to further characterize the tumour-antigen (TRP2) specific T cells induced by anti-PLA2G2D treatment in the LN6-987AL melanoma model. Although the tumour-specific T cell population that infiltrated the tumour at day 13 showed a uniformly activated $CD44^+Ki\text{-}67^+CD25^+PD\text{-}1^+$ phenotype, these cells remained TOX negative (**Reviewer Fig.3**). These results support that effects of PLA2G2D on T cell exhaustion may be limited, although the specific effects of PLA2G2D on T cell dysfunction will require dedicated in-depth follow-up studies. Considering the above evidence, we have rephrased our statements regarding the relationship between PLA2G2D and T cell exhaustion to better reflect these new findings (also see our response to comments above). We have integrated the data shown in **Reviewer Fig.3** and its interpretation in the manuscript (**Extended Data Fig.9b**, **Summary paragraph line 36**, **page 2 line 95, page 5 line 220, page 6 lines 273-278**).

Reviewer Figure 3

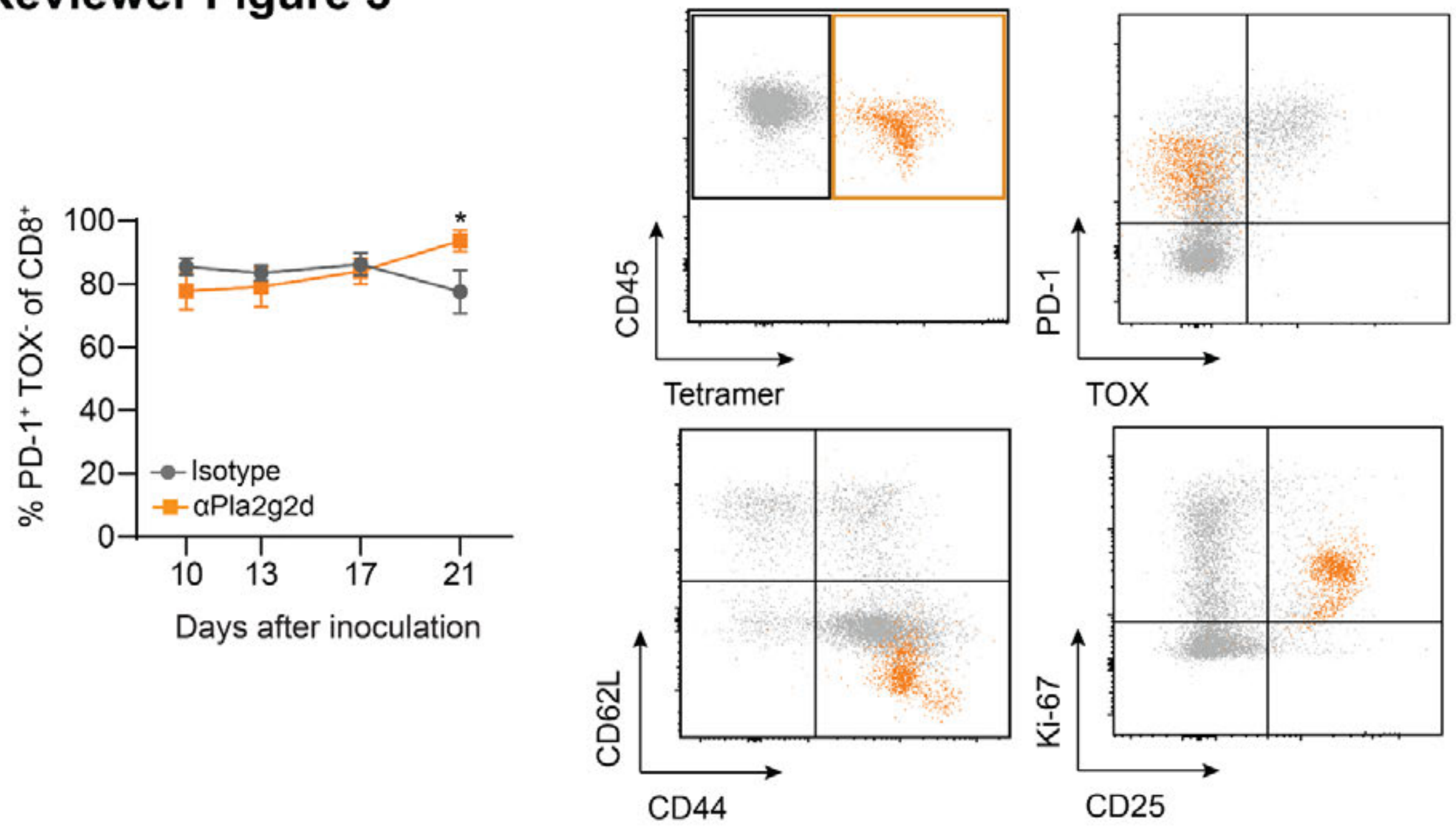

Reviewer Fig.3. *(**a**) Longitudinal immune-monitoring in isotype or anti-PLA2G2D antibody-treated animals inoculated with LN6-987AL LN-metastatic melanoma (experimental setup as in **Fig.4a**). Left: Line graph depicting percentages of PD-1^{+}TOX^{-} CD8^{+} T cells in the tumour, x-axis denotes days after tumour inoculation. Right: scatterplots showing protein levels of the indicated markers using flowcytometry. TRP2 tetramers recognize tumour-specific T cells, which are highlighted in orange across the plots.*

The gating for PD-1 in Reviewer figure 1 is not appropriate. The gate should be slightly higher.

Response: We adjusted the gate as requested, reanalysed the data and modified the manuscript accordingly (**Extended Data Fig.6c**).

The authors used anti-CSF1R to deplete "tissue resident macrophages". However, they did not show whether it indeed depletes these TAM from the tumors. Could they please confirm the efficiency of the treatment and the lack of effect on LNM? Most of the analysis was performed by checking RNA levels. It sometimes does not correlate with the protein expression.

Response: We agree that these data are very relevant to interpret anti-CSF1R depletion experiments. We therefore performed a new independent analysis of tumours and TDLNs upon CSF1R-depletion to quantify the abundance of different macrophage populations. We expanded our flow cytometry panel to more extensively characterize LNM subsets. CSF1R-receptor depletion using 100 µg injected twice-weekly strongly decreased tumour-associated macrophage (TAM) frequencies. In contrast, effects on LNM-subsets varied, with reductions observed in subcapsular and medullary sinus macrophages but only limited effects on medullary cord and T cell zone macrophages (**Reviewer Fig.4a**). Of note, using a 5-fold higher dose of anti-CSF1R antibody did not improve depletion efficiencies. Inspired by work from Baratin *et al.* (Immunity, 2017), we noted a striking enrichment of T cell zone macrophage (TZM) signature markers among genes upregulated in Pla2g2d^{pos} vs. Pla2g2d^{neg} LNMs. These included signature marker Timd4, Pla2g2d itself but also the broader TZM-specific transcriptional signature (**Reviewer Fig.4b-e**). Thus, anti-CSF1R antibodies cannot deplete Pla2g2d-expressing LNMs/TZMs, and the Baratin *et al.* study highlights that unfortunately no tools currently exist to efficiently target TZMs – identifying an important goal for future research. [redacted] e have integrated these observations into the revised manuscript

(**Extended Data Fig.8c-d** & **Fig.4n-p**), including the Results (**page 4 lines 196-204**) and Discussion sections (**page 6 lines 269-278**).

Reviewer Figure 4

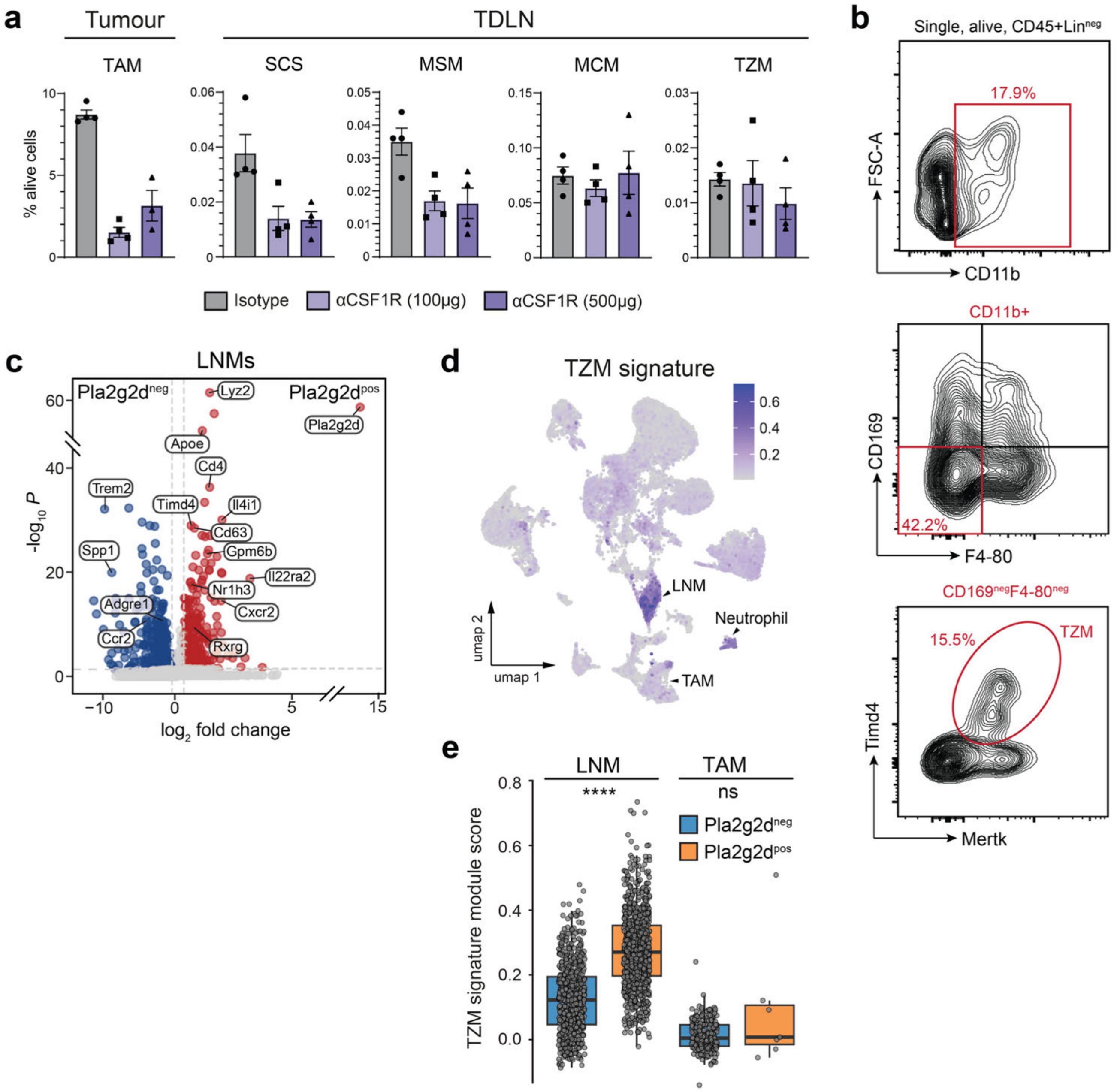

Reviewer Fig.4. *(**a**) Flow cytometry-based quantification of tumour-associated macrophages (TAM) and 4 TDLN macrophage subsets: subcapsular sinus (SCS), medullary sinus (MSM), medullary cord (MCM) and T cell zone (TZM) macrophages in mice (n=4) bearing LN6-987AL LN-metastatic melanoma. Animals were injected with isotype control or the indicated dose of anti-CSF1R depleting antibodies. Cells were pre-gated on single, alive, CD45^{+}, lineage-negative (CD19, CD3, CD49b) and CD11b^{+}, and further defined as Ly6G^{neg}F4/80^{+}CD64^{+} (TAM), CD169^{+}F4-80^{neg} (SCS), CD169^{+}F4/80^{+} (MSM), CD169^{neg}F4/80^{+} (MCM) and CD169^{neg}F4/80^{neg}Mertk^{+}Timd4^{+} (TZM). (**b**) Representative gating strategy of TZM from the TDLN of LN6-987AL melanoma-bearing mice. (**c**) Volcano plot of genes differentially expressed ($P < 0.05$, pseudo-bulk analysis) between lymph node macrophages (LNMs) with detectable (Pla2g2d^{pos}) or undetectable (Pla2g2d^{neg}) Pla2g2d RNA. Selected genes linked to LNM subset identity are highlighted. Note the many TZM markers (e.g. Timd4, Apoe, Cd4, Il22ra2) upregulated in Pla2g2d^{pos} LNMs. (**d**) UMAP representation of single cell transcriptomes from of tumours, TDLNs and non-draining LNs from mice 12 days after subcutaneous inoculation of B16F0 or LN6-987AL melanoma cells. Expression levels of the T cell zone macrophage (TZM) gene signature defined by Baratin et al. (Immunity, 2017)*

*are projected onto the UMAP. (**e**) Macrophages from the lymph node (LNM) or tumour (TAM) were subdivided into those with detectable Pla2g2d RNA (Pla2g2d^{pos}) or not (Pla2g2d^{neg}). Expression levels of the TZM gene signature in these macrophage subgroups are shown.*

I agree with the authors that in mice, the LMN population is quite likely the source of PLA2G2D, even if the authors did not perform a specific genetic inactivation in this population. However, in patients, it seems that the pattern of expression is a bit different with this expression in DCs. Additionally, one aspect that is not covered by the authors is the possible role of PLA2G2D in tertiary lymphoid structures. Could they find some expression of PLA2G2D in these structures, together with the formation of triads as described in TDLN in patients or is it really specific to TDLN.

Response: The referee is correct that whereas LNMs are the dominant source of PLA2G2D in our mouse model of LN-metastatic melanoma, in patient TDLNs we also see recurrence-associated upregulation in CD11c$^+$ DCs. We believe a plausible explanation for this is the previously described age-dependent increase in Pla2g2d synthesis by CD11c$^+$ DCs (Vijay *et al.* J Exp Med 2015). In mice, the Perlman group showed pronounced induction of Pla2g2d in DCs obtained from middle-aged (i.e. 10-13 months old) animals. In contrast, Pla2g2d levels were very low in DCs from young mice, which were of similar age as the animals we used for our *in vivo* tumour model experiments (8-12 weeks). Extrapolating these findings to humans, the middle-aged melanoma patients analysed in our study (avg. 50-55 years) may exhibit age-dependent increased levels of PLA2G2D$^+$ CD11c$^+$ DCs, which is then likely further exacerbated by chronic immune activation and/or oxidative stress. We have integrated this hypothesis in the revised manuscript (**page 6 lines 282-283**). Of note, when examining *PLA2G2D* expression in publicly available patient (TD)LN single cell RNA-Seq datasets, we again observed that the PLA2G2D signal predominantly originates from LNMs (please see our response to referee #3 & **Reviewer Fig.6**).

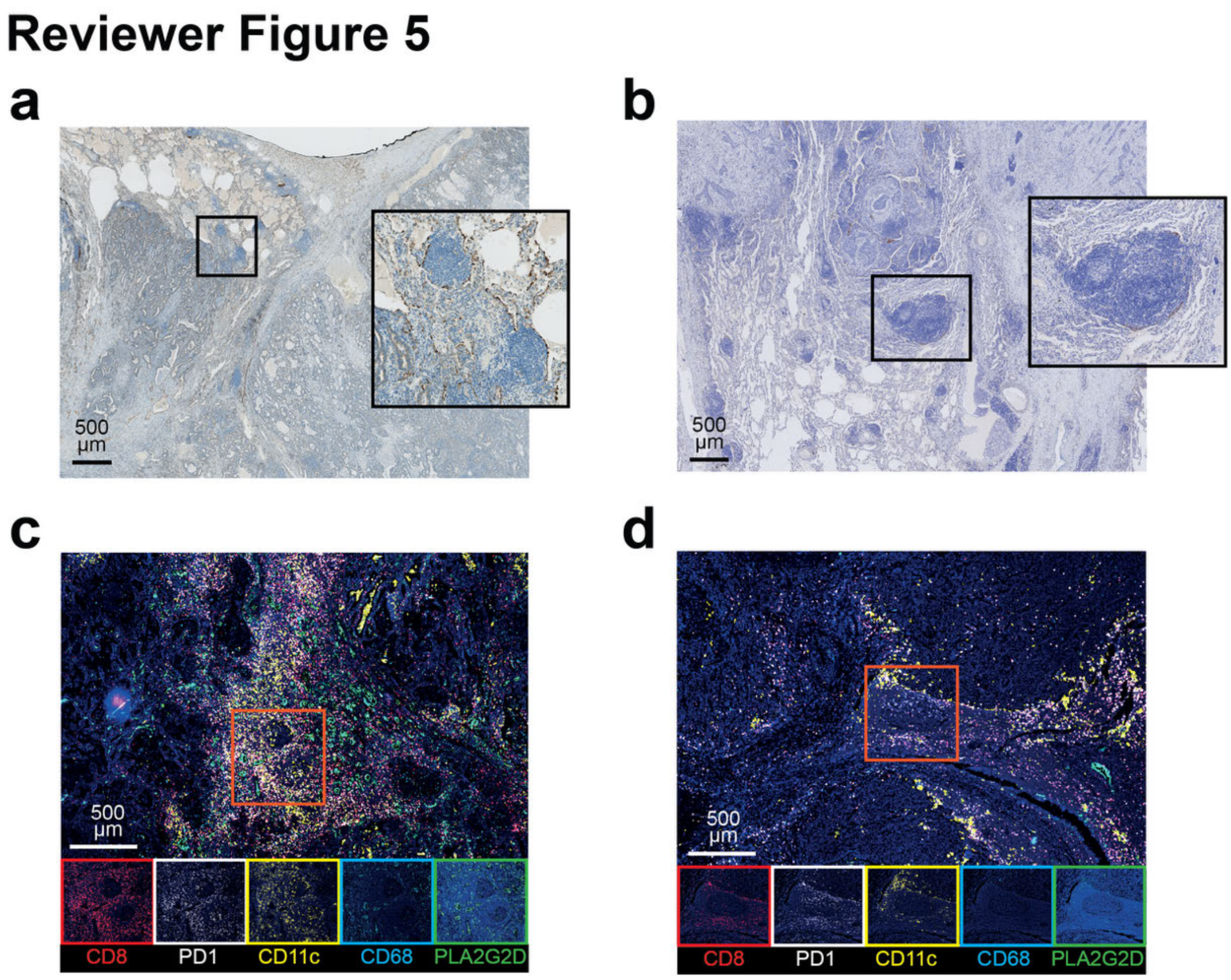

***Reviewer Fig.5**. (**a,b**) Immunohistochemistry of NSCLC tumour tissue stained for PLA2G2D (monochrome DAB stain). Zoom-ins depict areas in which TLS-like structures can be observed. (**c,d**) Multiplex immunofluorescence staining for the indicated proteins on NSCLC tumour tissue from non-metastasized (panel c) or metastasized (panel d) lymph nodes. Boxed areas harbour TLS-like structures.*

We appreciate the suggestion regarding PLA2G2D in tertiary lymphoid structures (TLSs). We reanalysed our chromogenic and immunofluorescence stainings (see **Fig.2f-h**) to try and identify TLSs in the images. Based on typical morphological characteristics, we did observe TLS(-like) regions in several of our tumour samples. Closer inspection of these candidate TLSs revealed limited expression of PLA2G2D in comparison to TDLNs (**Reviewer Figure 5**). However, our current stainings are not compatible with formal TLS identification and accurate staging of TLS maturity (e.g. lack of $CD4^+$ T cell, B cell and high endothelial venule markers). Further study of PLA2G2D function in TLSs will be interesting to pursue in dedicated follow-up experiments.

The author used pla2gd2 -/- mice injected with various tumor cell lines (as previously done by another group). They do not discuss how this KO could affect the immune system in untreated mice. Does this KO have a general effect or does not display any phenotype?

Response: Extensive previous work by the group of co-author Dr. Makoto Murakami has shown that *Pla2gd2*$^{-/-}$ mice show normal gross development, survival and behaviour (Miki *et al.* J Exp Med 2013). Importantly, knockout animals are virtually indistinguishable from their wildtype counterparts in terms of immune cell composition in spleen, various lymph nodes and thymus when examined under normal conditions (Miki *et al.* J Exp Med 2013).

If I understood well, PLA2G2D is also expressed in LN at steady states. Can the author make this point clearer. It is higher in elderly related to increased inflammation and was shown to be involved in tumor progression. Do they know whether it is also more expressed after infection? Is it a natural mechanism to dampen the immune system such as PD-1 expression?

Response: Apart from the highest levels of PLA2G2D detected in TDLNs, we also detected *Pla2g2d*$^+$ LNMs in non-draining LNs in a mouse model of melanoma (see **Fig.4k-l**). However, non-draining LNs in tumour models may still be subjected to systemic alterations evoked by a tumour, e.g. elevated inflammatory mediators in the circulation, and therefore do not necessarily resemble steady-state LNs. Alternatively, *Pla2g2d* gene expression may not only be induced by draining tumour cells but also by apoptotic cells in general, functioning as a potential mechanism that limits priming of T cells to auto-antigens (Baratin *et al.* Immunity 2017). We ourselves have not investigated Pla2g2d levels in LNs from naive animals not bearing tumours, although previous work has reported Pla2g2d-expressing myeloid cells in LNs from untreated, naive mice (Miki *et al.* JEM 2013, Baratin *et al.* Immunity 2017).

Referee #2

The study as presented represents a huge corpus of work. The key results have not changed, however the additional work has strengthened the conclusions. The spatial analysis of patient tumor draining lymph nodes is technically impressive and the analysis is enlightening. The data is analysed appropriately throughout. Concerns re. statistics have been addressed and the conclusions are robust and valid. I would not require any further revision to view this as a complete body of work ready for publication. The authors have done an impressive amount of work to address the concerns which were raised in the first round of review. As before, the paper is well written and clear.

Response: We are very pleased to read that the referee is very positive of our revised manuscript.

Referee #3

Krimpen et al have conducted a wide-range of experiments and analysis, including merging data from Umetani et al manuscript. All my major concerns have been satisfactorily addressed, and the new combined manuscript brings much needed mechanistic insights into the immunomodulatory role of myeloid-derived PLA2G2D in the tumour-draining lymph nodes (TDLN).

To summarize the authors have:

• Clarified why the recurrence models were not used.
• Included data from larger validation cohorts and a different cancer type (NSCLC).
• Compared findings in the TDLNs and tumor (Fig. 2), and showed that expression of PLA2G2D was highest in the CD11c+ DC and CD68+ Macrophages present in the TDLN, thus supporting a TDLN-specific checkpoint.
• Highlight interactions between PLA2G2D+CD11c+ DCs and CD8 T cells in TDLN from melanoma patients (Fig. 2).
• Included extensive mouse model data and functional studies from the Umetani et al manuscript to strength the mechanistic aspects (Fig.3-4).
• Added supporting data from a LN-metastatic melanoma model - highlighting concordant features with stage III melanoma.
• Identified a specific myeloid cell type with low CSF1R expression as the key cellular source of PLA2G2D.
• Defined the cell types mediating the immunosuppressive effects using bone marrow chimer and cell-depletions.
• Demonstrated effects of PLA2G2D-deficiency on tumor-specific T cells in vivo.
• Confirmed synergistic effects with anti-PD1 treatment.
• Included humanized mouse model data and human-specific antibodies to support translational relevance.

Although the mechanisms of PLA2G2D induction in myeloid cells, the nature of its cognate receptor, and down-stream signaling on T cells are not fully characterized, there is sufficient novelty and significance in the current body work presented in the revised manuscript.

Response: We appreciate the referee's positive assessment of the quality of our revisions and the relevance of our findings for the field.

Few minor points to consider:
1. Do the authors find the Pla2g2d-enriched macrophage population identified in mice TDLN (Fig. 4i,j) in human TDLN or tumors? Are they transcriptionally similar or different. A brief statement about this will be sufficient.

Response: We performed GSEA analyses to test whether the Pla2g2d-enriched LNM population we identified in mouse models shows transcriptional resemblance to the TDLN paracortex macrophage population associated with disease recurrence in melanoma patients. Indeed, a statistically significant enrichment was detected (normalized enrichment score [NES] = 1.35, adjusted *P* value = 0.007), as shown in **Fig.4m**. We discuss this finding in the text on **page 4 lines 194-196**.

2. Most data in humans is derived from melanoma and NSCLC, do the authors speculate that this mechanism is generic to many human cancer types? if they have any data or even re-analysis of published data, it will be helpful to highlight this in the manuscript.

Response: Given the similar association between PLA2G2D^{+} myeloid cell abundance in TDLNs and disease recurrence in both melanoma and NSCLC patients (see **Fig.2**), as well as the tumour type-overarching therapeutic effect of anti-Pla2g2d therapy in the various *in vivo* models we tested (see **Fig.3** & **Fig.5**), we indeed believe – as stated by the reviewer – that the immunosuppressive action of PLA2G2D in TDLNs is likely relevant for many human cancer types. To further substantiate this hypothesis, we mined published transcriptomics datasets in which TDLNs (and tumours) were analysed. These analyses revealed that *PLA2G2D* expression was indeed largely confined to a subgroup of macrophages in a lung adenocarcinoma (NSCLC) and in a breast cancer patient TDLN dataset (**Reviewer Fig.6**). These observations strengthen the concept of PLA2G2D as a tumour type-agnostic TDLN-centred immune checkpoint. We have added these data to the revised manuscript (**Extended Data Fig.8a-b**, **page 4 lines 196-198**).

Reviewer Figure 6

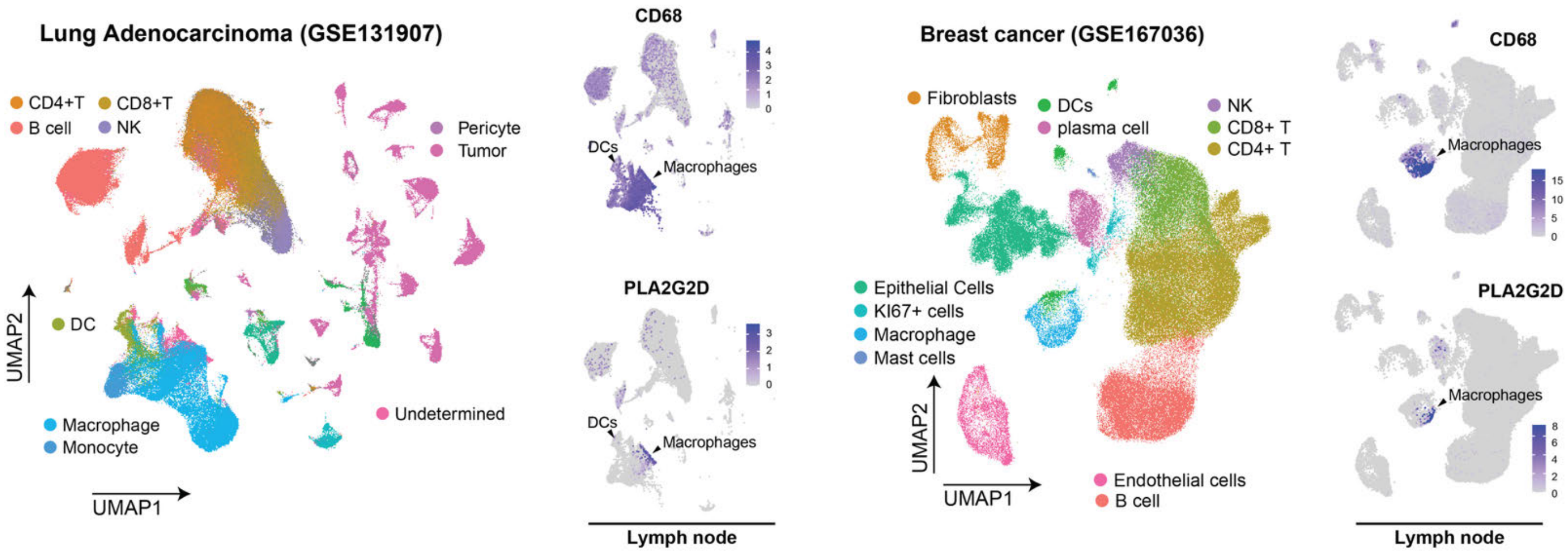

***Reviewer Fig.6**. Analysis of published single cell transcriptome datasets from lung adenocarcinoma (panel **a**) and breast cancer (panel **b**) tumour-draining lymph nodes, with UMAP showing cellular diversity within the full datasets. CD68 expression is depicted to highlight the LNM population, in which the bulk of the PLA2G2D-expressing cells reside.*

Recommend publication.

Response: We very much appreciate the referee's supportive statement.

Point-by-point response letter – 2024-10-22118D (Van Krimpen, Huang *et al.*)

Referee #1

The authors answered all the points raised during the revision. I congratulate them for their work. I am satisfied with their answers.

Response: We thank the reviewer for the constructive feedback throughout the review process.
